# Role of Hydration in Uncovering the OER Activity of Amorphous Iridium Oxide Electrocatalysts

**DOI:** 10.1021/acscatal.5c05765

**Published:** 2025-12-19

**Authors:** Connor Sherwin, Veronica Celorrio, Alessandro Difilippo, Katie Rigg, Mark Clapp, Armando Ibraliu, Luke Luisman, Thomas Wakelin, Amber Watson, Nikolay Zhelev, Lucy McLeod, Christopher M. Zalitis, Andrea E. Russell

**Affiliations:** † Department of Chemistry, 7423 University of Southampton, Southampton SO17 1BJ, United Kingdom; ‡ 120796 Diamond Light Source Ltd, Didcot OX11 0DE, United Kingdom; § Johnson Matthey Technology Centre, Reading RG4 9NH, United Kingdom

**Keywords:** OER, IrO_2_, catalysts, electrochemistry, in situ XAS, water electrolysis

## Abstract

Understanding the structural properties of iridium oxide electrocatalysts under operational conditions is critical for elucidating the structure–property relationships that enhance the catalytic activity for the oxygen evolution reaction. In this study, *in situ* X-ray absorption spectroscopy under realistic conditions was employed to investigate the potentiodynamic and time-resolved structural evolution of a commercial iridium oxide, alongside its fully hydrated and crystalline counterparts. Our findings reveal two distinct electrochemical regimes, a low potential plateau associated with a nonconductive Ir^3+^ state and a linear region where small potential variations induce reversible oxidation state and structural transformations. The structural changes were found to occur reversibly on the commercial material even after prolonged exposure to OER potentials. Notably, the hydrated IrO_*x*_ exhibits extremely high OER activity, surpassing the commercial material by nearly an order of magnitude, yet it suffers from significant instability. In contrast, the crystalline IrO_2_ demonstrates poor activity as its catalytic performance appears to be confined to the surface. These findings highlight the critical role of hydration in modulating both activity and stability, offering valuable insights for the rational design of next generation iridium based OER catalysts.

## Graphical abstract

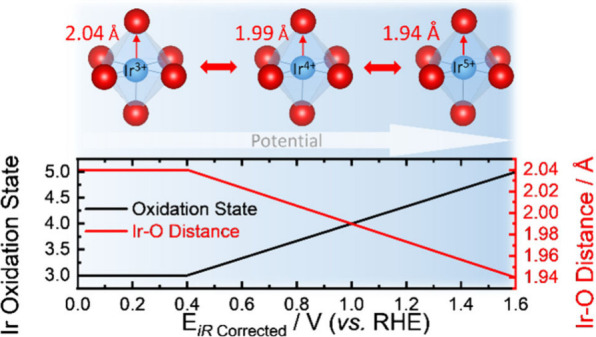

## Introduction

Water electrolysis powered with renewable electricity sources for hydrogen production is an essential technology for decarbonizing the chemical and transport industries both as a sustainable feedstock and as an energy vector. Proton exchange membrane water electrolysis (PEMWE) has emerged as a particularly promising technology due to its high power density and rapid load capability and is, therefore, projected to play a key role in the future green hydrogen market.^1,2^ Currently, iridium-based electrocatalysts are considered the most effective anode catalyst for PEMWE, demonstrating both high activity and stability during the acidic oxygen evolution reaction (OER). However, due to the scarcity of iridium, it is essential to thoroughly understand the factors contributing to its high activity and stability to optimize its performance.

A spectrum of iridium oxides can form, exhibiting different structural and electrochemical properties depending on the thermal treatment that they undergo. These can be categorized into three main types of iridium oxide: hydrated, amorphous, and crystalline.^3^ Hydrous iridium oxide is formed at low temperatures, allowing for the retention of physisorbed and chemisorbed water within the structure. These materials tend to be very disordered and are synthesized through the potentiostatic cycling of iridium metal electrodes, resulting in an open hydrated structure.^4–6^ Various structural representations of the hydrated oxide exist, including IrO_*x*_, IrO_2_·4H_2_O, Ir­(OH)_4_·2H_2_O, and [IrO_2_(OH)_2_·2H_2_O]^2–^·2H^+^.^7^ The hydrated form is generally understood to adopt an octahedral coordination within an open polymeric network.^4,5^ Amorphous iridium oxide is formed following thermal treatments between 100–300 °C. During this process, water is removed from the structure, resulting in an amorphous material characterized by low electronic conductivity with some possible microcrystalline domains. As the calcination temperature increases further, crystalline domains with a rutile structure start to be formed, leading to improved conductivity. However, annealing at temperatures above 500 °C results in a loss of porosity due to sintering.

It is generally acknowledged that electrochemically formed hydrated iridium oxides exhibit significantly greater activity than amorphous or crystalline iridium oxides.^3^ This enhanced activity is attributed to the bulk redox properties, which facilitate the transport of water and ions deeper into the bulk structure, in contrast to the behaviors observed in the high-temperature rutile structure. This large effective interface between electrode and electrolyte results in substantial pseudocapacity, making these materials particularly interesting for supercapacitors.^8^ Using a variety of synthetic techniques, the activity of thermally treated iridium oxides is found to reach a balance between conductivity and surface area at temperatures ranging from 400–550 °C.^9–13^ Research has shown that the dehydration of hydrous iridium oxide through thermal treatment can be reversed by an activation procedure involving square-wave polarizations.^14^ This process can be viewed as the unravelling of the amorphous or crystalline structure, resulting in the formation of free, highly hydrated, edge-sharing [IrO_6_]^*n*^ chains.^15^ However, it is important to note that fully hydrated materials exhibit significant instability during prolonged OER.^3^

A promising strategy for enhancing the stability of iridium while preserving its high activity involves the use of partially hydrated materials. Therefore, a comprehensive understanding of the relationship between the degree of hydration, crystallinity, and the catalytic activity of iridium oxide is required.

In this work, we investigate the structural transformations of various hydrated, partially hydrated, amorphous, and crystalline iridium oxides using *in situ* X-ray absorption spectroscopy (XAS). Through potentiodynamic measurements and time-resolved XAS studies, two distinct regions were identified in the potential versus oxidation state plots, corresponding to the transition of hydrated catalysts from low-conductivity Ir^3+^ species to highly conductive Ir^4+^ species. The variations observed in these plots for the different catalysts highlight key structural differences that influence their activity as an OER catalysts.

## Experimental Section

### Catalysts

Four different Ir oxide electrocatalysts were used in this study. The first is a commercially available iridium oxide from Alfa Aesar (Premion, 99.99% purity, found to contain trace Ir^0^), termed Premion in this text. The second is a research sample of metal-free amorphous iridium oxide provided by the Johnson Matthey Technology Centre, denoted as IrO_*x*_ (Ir^0^ free). The third is rutile IrO_2_ obtained by calcining Premion at 800 °C, denoted as Premion (800 °C). The final catalyst was synthesized via base hydrolysis of a hydrogen hexachloroiridate salt and is denoted as Hydrated IrO_*x*_.

### Synthesis of the Hydrated IrO_*x*_

To prepare the Hydrated IrO_*x*_, 60 g of hydrogen hexachloroiridate salt (22.43% wt Ir aqueous solution, Johnson Matthey, Product code: 161001 Batch Number: EJ0012) was heated to 90 °C under constant stirring. 1315.5 g of 0.4 M NaOH solution was added over 1 h to the iridium solution and left for 8 h at 90 °C. The reactor was left to cool to ambient temperature and the solid collected by vacuum filtration. The precipitate was never left to completely dry and was washed several times until the filtrate conductivity was below 50 μS cm^–2^, after which a sample of the “gel-like” iridium was prepared into an ink.

### Preparation of Floating Electrodes

All floating electrodes were prepared by dispersing the catalyst (1 wt %) and Nafion ionomer (0.12 wt %) in 22% 1-propanol/water mixture by ultrasonication for 5 min. The particle size distribution of the ink was measured (MasterSizer3000) to ensure that the DW(90) was below 10 μm. The ink was then diluted with 22% 1-propanol/water to a catalyst concentration of 5 μg mL^–1^. A polycarbonate track-etched (PCTE) membrane (Cytiva, 10417712) was sputter coated (Quorum Q150TS) with 100 nm of Au and subsequently cleaned in a Soxhlet extractor by first refluxing in water and second with isopropanol, both for 8 h. Au is used as a stable and conductive support material with minimal contribution to the OER activity. The membrane was subdivided and the catalyst ink vacuum-deposited onto the membrane to a geometric area of 0.314 cm^2^. Electrodes were prepared with two different target loadings, a lower loading (10 μg_cat_ cm^–2^_geo_) for activity tests, and a higher loading (50 μg_cat_ cm^–2^_geo_) for voltammetry tests. The metal loadings were determined by XRF mapping (Fisherscope X-ray XDV). A 0.1% Teflon AF-2400 (Sigma-Aldrich, 469629) in Flourinert FC-40 (Sigma-Aldrich, F9755) was coated on the underside of the electrode to a loading of 6 μg_cat_ cm^–2^_geo_ and dried in a vacuum oven overnight at 80 °C.

### Electrochemical Measurements Using the Floating Electrode (FE) Setup

All floating electrode tests were conducted in N_2_ saturated 1 M H_2_SO_4_ (Sigma-Aldrich Auprapur, 1.00714) under constant N_2_ purging at a flow rate of 25 mL min^–1^ at 25 °C. A Biologic VSP potentiostat was used in a three-electrode configuration with a platinum mesh counter electrode and an in-house-prepared reversible hydrogen electrode (RHE) as the reference electrode. All glassware, counter and reference electrodes were cleaned in Nochromix (Sigma-Aldrich, Al-Nochromix sachets, 328693) solution overnight, rinsed and boiled in ultrapure water (18.2 MΩ cm^–2^) six times. All electrodes were conditioned by cycling 35 times between 1.300 and 1.705 V_RHE_ at 50 mV s^–1^. The activity measurements were conducted on the low-loading electrodes, consisting of 3 cyclic voltammograms (CVs) between 1.300 and 1.705 V_RHE_ at 10 mV s^–1^. The potential is automatically corrected for ohmic drop at 85% during testing (single point Potentiostatic Electrochemical Impedance Spectroscopy (PEIS) at 250 kHz, 1.3 V_RHE_) and manually corrected post processing using the high-frequency intercept of the PEIS (1 MHz to 100 mHz frequency range at 1.6 V_RHE_). The high-loading electrodes were used to test the scan rate dependence of the CVs between 0.05 and 1.30 V_RHE_. Three cycles were taken at each scan rate (300, 200, 100, 75, 50, 25, 10, 5, and 1 mV s^–1^).

The exact metal loadings measured by XRF for the low- and high-loading electrodes are presented in Table 1.

**Table 1:** XRF Iridium Metal Loadings of the Low- and High-Loading Floating Electrodes

	XRF Loading/μg_Ir_ cm^–2^	
Catalyst	Low	High
Premion	10.3	42.3
IrO_*x*_ (Ir^0^ Free)	10.3	58.5
Premion (800 °C)	12.5	53.7
Hydrated IrO_*x*_	13.0	49.4

### *Ex Situ* Characterization

X-ray diffraction (XRD) of the catalyst powders was carried out on a Bruker D8 Advance with a Bragg Brantano geometry, a Cu Kα (λ = 1.5406 + 1.54439 Å) radiation source, and a step size of 0.02°. X-ray diffraction data were analyzed in the Bruker-AXS TOPAS 5 software and crystallite sizes determined by Pawley refinement.

Thermogravimetric analysis (TGA) was collected on a TGA 5500 (Discovery TGA 5500 – TA Instruments Thermal Analysers) with a 5 °C min^–1^ ramp rate from ambient temperature up to 850 °C under a N_2_ flow (100 mL min^–1^)

Scanning transmission electron microscopy (STEM) measurements were made on a JEM 2800 (scanning) transmission electron microscope at 200 kV and a C2 aperture of 70 and 40 μm. Samples were prepared by grinding between two glass slides and dusted onto a holey carbon-coated Cu TEM grid.

### Preparation of *in Situ* XAS Electrodes

The working electrodes for the *in situ* XAS measurements were prepared using the same method as described in a previous publication.^16^ A polytetrafluorethylene (PTFE) membrane (Fisherbrand, 0.45 μm, 47 mm) was sputter coated (Quorum Q150TS) with 100 nm of Au, which acts as the current collector. The ink is then spray coated on top of the Au layer to a geometric area of 3.14 cm^2^ using a stencil. The iridium ink suspension consisted of the catalyst, Milli-Q H_2_O, IPA and 5 wt % Nafion ionomer solution which was homogenized by milling then by ultrasonicating for 5 min. The mass ratio of catalyst to Nafion ionomer was kept consistent throughout all electrodes to avoid the influence of the ionomer on hydration and ion transport. The iridium metal loadings were measured by X-ray fluorescence (XRF) mapping (Fisherscope X-ray XDV) and the results are presented in Table 2.

**Table 2:** XRF Iridium Metal Loadings of the *in Situ* XAS Electrodes

Catalyst	XRF Loading/μg_Ir_ cm^–2^
Premion	190
IrO_*x*_ (Ir^0^ Free)	170
Premion (800 °C)	410
Hydrated IrO_*x*_	170

### *In Situ* Electrochemical XAS Setup

All *in situ* experiments were conducted using the previously published SPEC-XAS cell,^16^ which features an improved three phase interface for efficient bubble management during the OER. Prior to experiments, the cell, platinum mesh counter electrode and electrolyte reservoir were cleaned in Nochromix (Sigma-Aldrich, Al-Nochromix, 328693) solution overnight, rinsed and boiled in ultrapure water three times. An Ivium OctoStat200 potentiostat connected to an OctoBoost16000 booster for high current experiments was used for all *in situ* testing. A Gaskatel RHE (Hydroflex) was used as the reference electrode, and a platinum mesh (50 × 50 mm) was used as the counter electrode. A continuous flow syringe pump (ChemYX Fusion 4000) was used to supply a pulse free electrolyte from a central reservoir to the cell. A 1 or 0.1 M H_2_SO_4_ electrolyte was used for all measurements at a flow rate of 5 mL min^–1^. N_2_ gas was continuously flowing through the gas side of the cell and into the electrolyte reservoir at 50 mL min^–1^ throughout the experiment to purge out any oxygen. Prior to any XAS measurements, the electrodes were conditioned by cycling between 0 and 1.35 V_RHE_ at 200 mV s^–1^ for 150 cycles.

### X-ray Absorption Spectroscopy

*Ex situ* and *in situ* XAS spectra were collected at the B18 beamline at the Diamond Light Source. A monochromatic beam is provided by a 1.4 T bending magnet and collimated by a water-cooled Si mirror. The fixed exit double crystal monochromator is water cooled and consists of a Si(111) crystal. The optics are split into two branches where a Cr coated double toroidal focusing mirror was used for the Ir and Pt L_III_ edges. For measurements at the Ir L_III_ edge, the X-ray energy was calibrated with a Pt reference foil at the Pt L_III_ edge. For *ex situ* measurements, the samples were made into pellets using cellulose and measured in the transmission mode. *In situ* measurements were conducted in fluorescence mode using a 36 element Ge fluorescence detector with the monochromator in quick extended X-ray absorption fine structure mode (QEXAFS) with each scan taking approximately 30 s, excluding the dead time for the monochromator to reposition. Overall, the time resolution of the XAS measurements was approximately 45 s. The cell was positioned so that the sample was orientated at a 45° angle to the beam and the detector.

### XAS Data Analysis

X-ray Larch was used for the XAS data alignment, normalization, background subtraction and EXAFS fitting.^17^ For the *ex situ* pellet data, the Fourier transform *k*-range was kept between 3 and 15.3 Å^–1^ while for *ex situ* electrode data and *in situ* data the *k*-range was kept at 3–12.0 Å^–1^ during fitting and a *k*-weight of 3 was used to optimize the fit. An amplitude reduction factor of *S*_0_^2^ = 0.78 was calculated from the fitting of a crystalline IrO_2_. This was then fixed during the fitting of all experimental data while the coordination number, bond distance, Debye–Waller factor, and energy parameters were allowed to vary. The model used for fitting all the EXAFS data is shown in Table S1.

## Results and Discussion

### *Ex Situ* Characterization

A range of techniques were employed to characterize the differences among the various iridium oxides and to understand how the presence of iridium metal, hydration, and crystallinity affect their electrocatalytic performance. Four iridium oxide materials were studied: a commercial material from Alfa Aesar (Premion), an amorphous iridium oxide supplied by Johnson Matthey Technology Centre without any iridium metal in the sample (IrO_*x*_ (Ir^0^ Free)), the Premion sample after calcination at 800 °C (Premion (800 °C)), and a hydrated iridium oxide synthesized for comparison (Hydrated IrO_*x*_).

The powder X-ray diffractograms (XRD) of Premion, IrO_*x*_ (Ir^0^ Free), and Premion (800 °C) are presented in Figure 1. The XRD demonstrates that both Premion and IrO_*x*_ (Ir^0^ Free) are amorphous oxides, with the former exhibiting contributions from metallic iridium, while the calcined sample (Premion (800 °C)) forms a crystalline rutile phase. The refinement of the Premion (800 °C) sample yielded an approximate IrO_2_/Ir ratio of 99/1, which aligns with estimates by Pfeifer et al.^18^ who reported a metal loading of around 2–3%. The effective removal of the metallic phase from amorphous IrO_*x*_ is demonstrated by the absence of iridium metal reflections in the IrO_*x*_ (Ir^0^ Free) sample. Notably, some metallic iridium remains in the Premion (800 °C) sample, even after calcination at 800 °C, which may be attributed to the incomplete oxidation or the reduction of IrO_2_ at high temperatures. Crystallite sizes were estimated from the refinement of the crystal structures and are summarized in Table 3. Both the amorphous Premion and IrO_*x*_ (Ir^0^ Free) samples were found to have crystallite sizes of less than 2 nm; a more precise determination is not possible due to their amorphous nature. In contrast, the crystallite size of the Premion (800 °C) sample increased significantly due to the sintering of crystallites at elevated temperatures. Note, limited data were collected for the Hydrated IrO_*x*_ due to difficulties in drying the sample for characterization without changing its structure.

**Figure 1:**
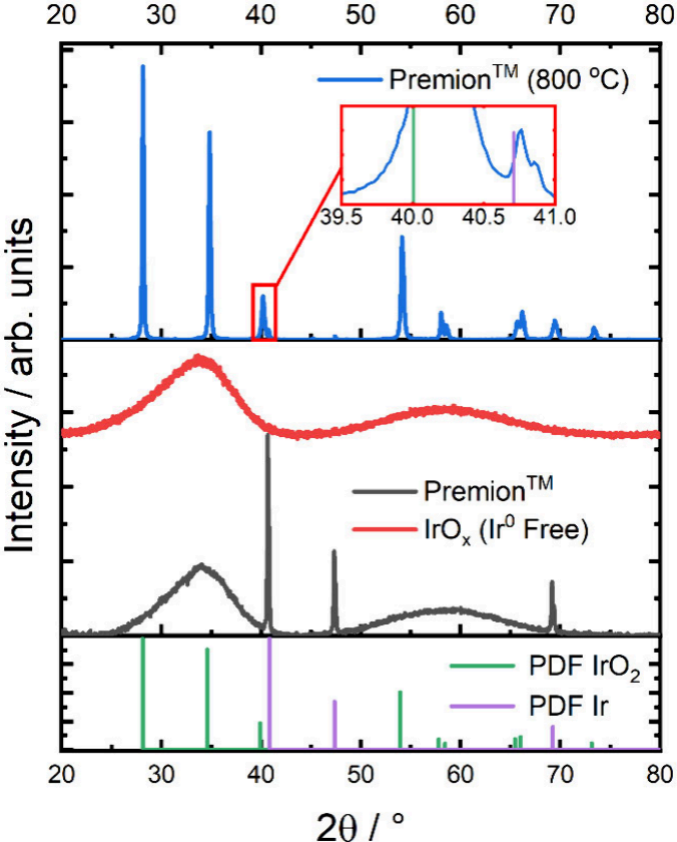
X-ray diffraction patterns of the commercial Premion, the iridium metal free sample (IrO_*x*_ (Ir^0^ Free)) and the Premion calcined to 800 °C (Premion (800 °C)). The inset shows the phase matched iridium metal that is retained after calcination. The reference diffraction patterns for IrO_2_ and metallic iridium used for phase matching are shown.

**Table 3:** Crystallite Size of IrO_*x*_ Catalysts Determined from the XRD, Particle Sizes Determined by TEM, and Surface Area Determined by BET

	Phase	Premion	IrO_*x*_ (Ir^0^ Free)	Premion (800 °C)
Crystallite Size/nm (XRD)	IrO_2_	<2	<2	42.4 ± 0.5
	Ir			130 ± 40
Particle Size/nm (TEM)	IrO_2_	12 ± 3	16 ± 4	33 ± 7
	Ir	83 ± 17		173 ± 43
Surface Area/m^2^ g^–1^ (BET)		25.06 ± 0.04	39.38 ± 0.04	1.98 ± 0.02

Thermogravimetric analysis (TGA) was conducted on the Premion sample, revealing that the amorphous structure consists of approximately 10% physisorbed and chemisorbed water (Figure S1). This value is in line with the 7.5 wt % previously suggested.^19^ It is therefore reasonable to assume that the Premion sample contains both hydrated and anhydrous domains within the structure, especially when electrochemically activated. This water is eliminated at calcination temperatures beyond 600 °C, which is consistent with the findings for the Premion (800 °C) sample.

Scanning transmission electron microscopy (STEM) images of the Premion, shown in Figure S2, indicate that large clusters of iridium metal, with an average diameter of 83 ± 17 nm, are present. In contrast, much smaller agglomerates of amorphous IrO_*x*_ particles are present in the Premion and IrO_*x*_ (Ir^0^ Free), ranging from 12–16 nm. In the Premion (800 °C) sample, the crystallite size and particle size are comparable within measurement error.

Analysis of the X-ray absorption near edge structure (XANES) data enables the determination of a material’s d-hole count by applying a linear fit to reference standards of known oxidation states. To accurately determine the d-hole count, six different standards were used: metallic Ir, Ir­(acac)_3_, IrO_2_, Sr_3_ZnIrO_9_, Sr_3_MgIr_2_O_9_ and Ba_2_SrIrO_6_ which have formal iridium d-hole counts of 3, 4, 5, 6 and 7, respectively. A more accurate determination of the d-hole count was achieved when the edge position was defined as the minimum of the second derivative. The Ir L_III_ edge arises from a 2p to 5d e_g_ transition, where the white line broadens due to changes in the crystal field splitting.^20,21^ In a perfectly octahedral environment, two transitions can occur corresponding to the t_2g_ and e_g_ states. By selection of the second minimum in the second derivative (when two transitions are occurring), only the difference in the 2p to 5d e_g_ transition is probed. This approach allows for a more precise calibration of the number of d-hole electrons (Figure 2). The normalized XANES data along with the second derivatives are presented in Figure S3. Using the method described above, a slope of 1.142 eV per d-band hole is extracted (as shown in Figure S4). This is comparable to several other studies, which have reported values in the range of 0.925–1.655 eV per d-band hole.^22–26^ These studies typically use IrCl_3_ as an Ir^3+^ standard; however, we find that it yields inconsistent results, likely due to the reduced covalency of the Ir–Cl bonds. More reliable data fits are obtained when Ir­(acac)_3_ is used as the Ir^3+^ standard (see Figure S5).

**Figure 2:**
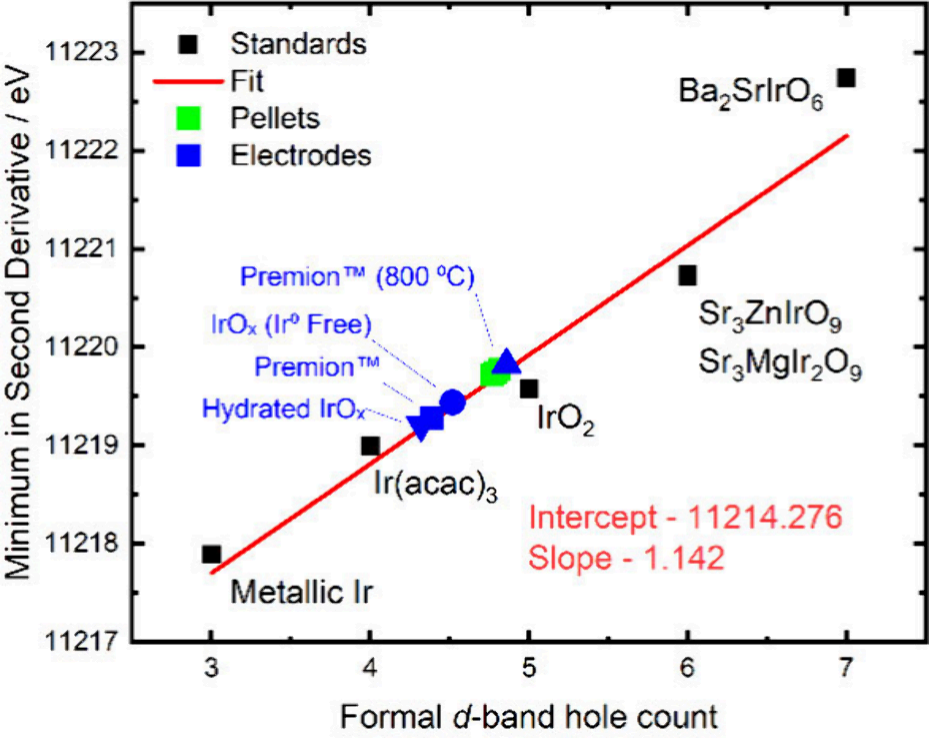
Calibration of the d-band hole count to the minimum in the second derivative for standards of iridium L_III_ edge XANES spectra. The edge positions for the pellets and electrodes, analyzed *ex situ*, are also indicated at their calculated d-band hole counts. The normalized XANES and second derivatives of each standard are shown in Figure S3. A pristine version of this plot, without the samples, is shown in Figure S4.

The Ir L_III_ edge XANES spectra for the materials, both as pristine pellets and as pristine electrodes, are illustrated in Figure 3. The corresponding calculated d-band hole counts and oxidation states are presented in Figure 2 and Table 4, respectively. The fitted XANES are shown in Figure S6 and Figure S7 and fitted parameters in Table S2 and Table S3. The oxidation state of the Premion has been reported in the literature using various different techniques, including XAS, EELS, and TPR, yielding values in the range 3.6–3.9.^19,27,28^ This range is corroborated by the current findings, which indicate that the pristine pellet of Premion has an average oxidation state of 3.77, suggesting the presence of iridium species with formal oxidation states of Ir^3+^ and Ir^4+^ in the amorphous powder. Furthermore, titration experiments conducted by Nahor et al.^29^ established the oxidation state of hydrated iridium oxide to be 3.2. This result supports the notion that Premion contains regions of hydrated iridium oxide characterized by lower valence iridium species. Nahor et al.’s findings are also consistent with the oxidation state of the Hydrated IrO_*x*_, measured at 3.32, indicating a highly hydrated starting structure which contains more hydrated and hydroxylated sites than the Premion or IrO_*x*_ (Ir^0^ Free) materials. There is a general reduction of approximately 0.34 unit in the average oxidation state for both Premion and IrO_*x*_ (Ir^0^ Free) when prepared as electrodes compared to their dry powder forms. This may be attributed to a surface hydration, hydroxylation process that occurs when the iridium is incorporated into the ink. Here, an equilibrium is established with the solution ions on the surface of the iridium and possibly extending into the bulk for the hydrated materials. In contrast, the oxidation state of the Premion (800 °C) sample remains approximately constant. The crystallite size of the Premion (800 °C) material is significantly larger, which may explain its reduced sensitivity to surface changes compared to the other materials

**Figure 3:**
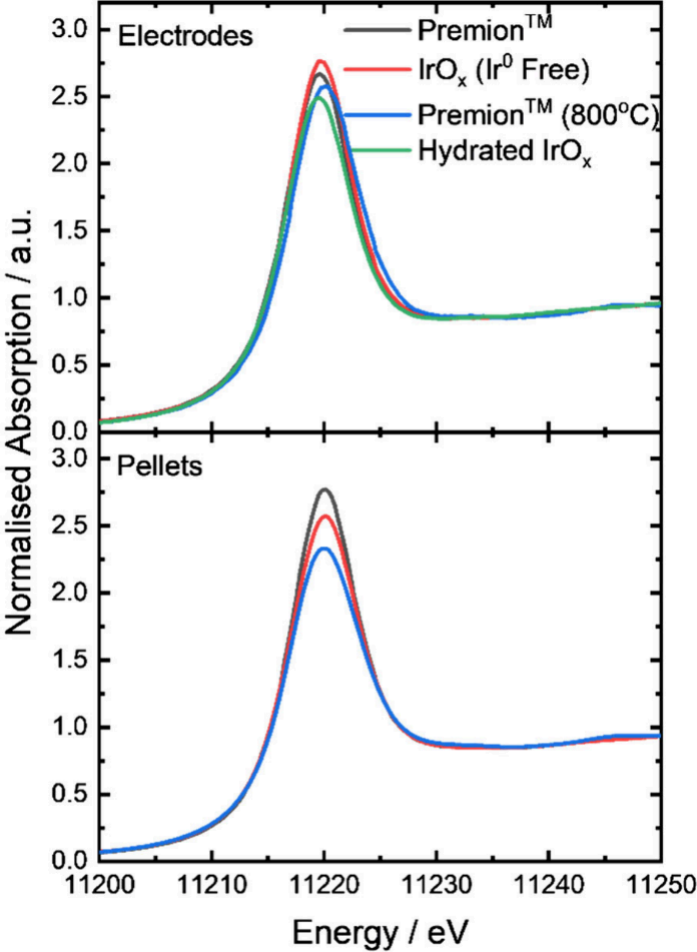
*Ex situ* Ir L_III_ edge XANES for the IrO_*x*_ materials for the dry powders as pressed pellets and for the when made into electrodes prior to any electrochemical treatment. The Hydrated IrO_*x*_ sample could not be prepared as a pellet and is therefore only shown after being prepared as an electrode.

**Table 4:** Average Oxidation State Comparison of the *ex Situ* Materials as Pellets of the Dry Powders and after Being Made into Electrodes Prior to Any Electrochemical Treatment

	Premion	IrO_*x*_ (Ir^0^ Free)	Premion (800 °C)	Hydrated IrO_*x*_
Pellet	3.77	3.81	3.79	
Electrode	3.39	3.52	3.88	3.32

The Fourier transform EXAFS of the pellets and electrodes is shown in Figure 4. As would be expected for the large crystallite size of the Premion (800 °C), peaks in the Fourier transforms are observed at a higher radial distance, indicating greater long-range order in the Premion (800 °C) sample compared to the amorphous Premion, IrO_*x*_ (Ir^0^ Free) or Hydrated IrO_*x*_ materials. The fitted EXAFS data for the pellets and electrodes are provided in the Supporting Information, Figures S8–S11 and Tables S4 and S5. The first shell Ir–O distances are detailed in Table 5. Consistent Ir–O distance values are shown for the pellets, in agreement with the minimal changes in the observed oxidation state. It is noted that the Premion and IrO_*x*_ (Ir^0^ Free) are reduced during the electrode fabrication process, and this reduction appears to correlate with the trend of increasing Ir–O bond lengths. This relationship is illustrated in Figure S12, which depicts the Ir–O distance plotted as a function of the oxidation state.

**Figure 4:**
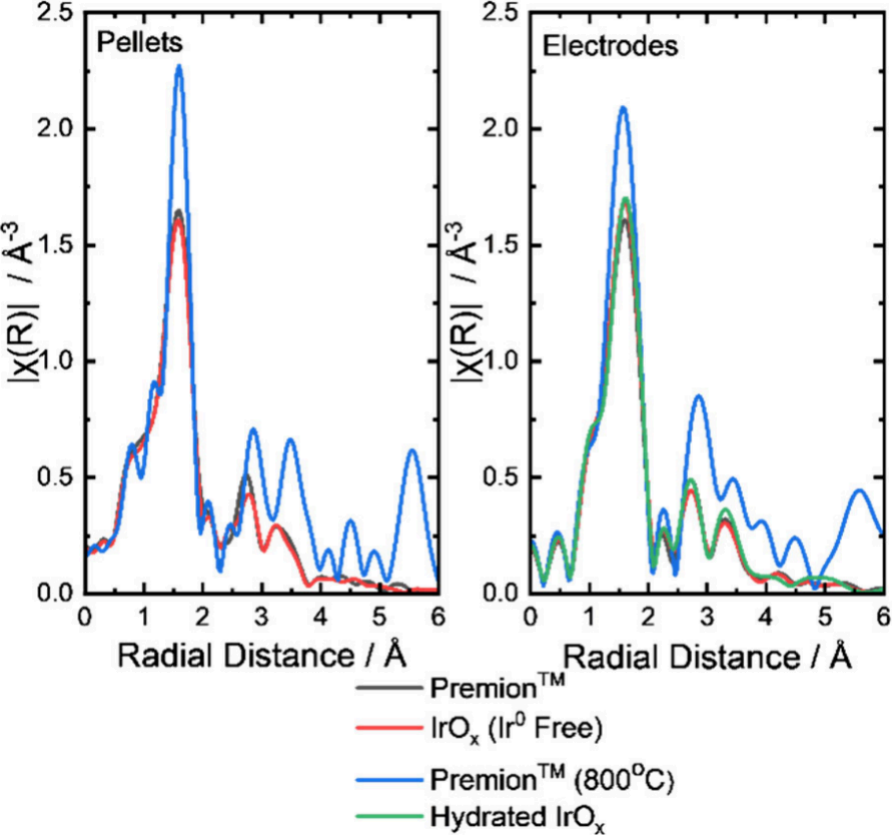
Fourier transform of the *k* ^2^-weighted Ir L_III_ EXAFS (not phase corrected) of the IrO_*x*_ catalysts as dry powders pressed into a pellet and when the sample is made into an electrode prior to any electrochemical treatment.

**Table 5:** First Shell Ir–O Distance (Å) from the Fitted EXAFS Data^a^

	Premion	IrO_*x*_ (Ir^0^ Free)	Premion (800 °C)	Hydrated IrO_*x*_
Pellet	1.982 ± 0.008	1.981 ± 0.006	1.975 ± 0.004	
Electrode	2.00 ± 0.01	2.002 ± 0.009	1.970 ± 0.008	2.014 ± 0.009

a Full fits are shown in Figure S8 and Figure S11 and fitted parameters are shown in Table S4 and Table S5. Pellet data are from the dry powder pressed into a pellet and the electrode is after the catalyst is made into an electrode prior to any electrochemical treatment.

### Electrochemical Performance

Electrochemical testing was completed by using the floating electrode (FE) technique. The floating electrode, which employs a gas diffusion electrode with very low catalyst loading (5–15 μg_Ir_ cm^–2^), allows the efficient gas removal from the electrode and minimizes mass transport limitations in oxygen evolution reaction measurements.

The mass normalized cyclic voltammetry data are presented in Figure 5. The voltammetry of the Hydrated IrO_*x*_ reflects the typical shape of hydrated iridium oxide, similar to those published for anodic iridium oxide films (AIROFs) and hydrated iridium oxide films (HIROFs), which have been the subject of extensive fundamental IrO_*x*_ studies.^6,7,30–35^ A characteristic Ir^3+^/Ir^4+^ redox couple is observed around 0.9–1.0 V_RHE_, resulting from the facile protonation of hydrous Ir^3+^ oxide (typically Ir­(OH)_3_ or HIrO_2_) forming IrO­(OH)_2_ or IrO_2_·H_2_O.^6,31,36^ This hydrous Ir^3+^ oxide exhibits low conductivity, behaving like a p-type semiconductor in depletion and resulting in a plateau in the current below 0.6 V_RHE_.^37–41^ The switching conductivity has been described by Gottesfeld, where the t_2g_ band is filled for the Ir^3+^ species, shifting the Fermi level to the band gap between the t_2g_ and e_g_ bands (“turned off”) while the Fermi level is in the t_2g_ band (“turned on”) for the Ir^4+^, which achieves metal like conductivity.^39,42^ This switching on and off in conductivity is accompanied by an electrochromic transition from a “bleached” state (turned off) to “colored” state (turned on).^30,41^ The lack of conductivity at low potentials accounts for the featureless appearance of the hydrated iridium oxide below the Ir^3+^/Ir^4+^ redox peak. electrochemical quartz crystal microbalance (EQCM) measurements of hydrated iridium oxide in this region also reveal a plateau in the frequency response below the main redox couple.^43,44^ Furthermore, a plateau has also been observed by Papakonstantinou et al.^45^ on Premion in EQCM measurements below 0.4 V_RHE_. The authors attribute this plateau to ORR on the gold crystal; however, it is plausible that this plateau may also arise from the formation of the low-conductivity Ir^3+^ state.

**Figure 5:**
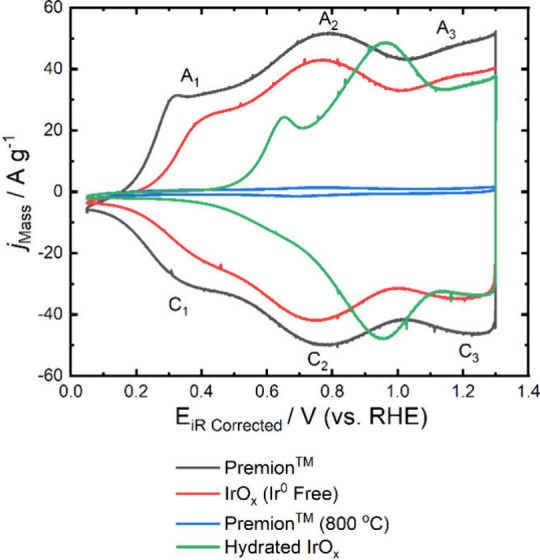
Mass-normalized cyclic voltammetry of the high-loading electrodes recorded at 0.05 V s^–1^. The CVs of the Premion, IrO_*x*_ (Ir^0^ Free) and Premion (800 °C) are all measured postconditioning while the Hydrated IrO_*x*_ is measured preconditioning. The iridium metal loading for all of electrodes is around 50 μg_Ir_ cm^–2^ with the exact loadings of each electrode shown in the Experimental Section.

The Premion and IrO_*x*_ (Ir^0^ Free) samples have the characteristic CV shape which has been the subject of many previous investigations.^18,19,27,28,45–52^ Three distinct redox peaks are identified in the CVs, labeled A_1_/C_1_, A_2_/C_2_, and A_3_/C_3_ in Figure 5. The A_2_/C_2_ and A_3_/C_3_ peaks are generally believed to arise from the redox transitions of Ir^3+^/Ir^4+^ and Ir^4+^/Ir^5+^ species, respectively.^50^ The A_1_/C_1_ peaks, however, remain poorly understood and will be discussed in more detail below. The primary difference between the Premion and IrO_*x*_ (Ir^0^ Free) CVs is the first redox peak, which is broader and has an onset around 60 mV lower than that in the metal-free sample. The CV of the Premion (800 °C) is featureless and has a very small capacitive charge. The capacitive charge of the Premion (800 °C) is sufficiently small that the current associated with the gold current collector is a significant contributor. The charge between 1.15 and 1.30 V_RHE_ of a 10 mV s^–1^ CV can be used to estimate the electrochemical area of an iridium oxide catalyst (Figure S13).^19^ The capacity of the Premion drops by 98% after calcination at 800 °C which is in good agreement with the drop in the BET surface area, dropping by 92% from 25 to 1.98 m^2^ g^–1^. The small difference between the capacitive charge and BET area drop can be attributed to the additional charge of the Au current collector contributing to the Premion (800 °C).

The Hydrated IrO_*x*_ sample also displays a prominent anodic prepeak, observed experimentally in thin films of hydrous IrO_*x*_, which has been shown to shift to higher potentials as film thickness increases.^44^ To investigate the prepeak further, the scan rate dependence of the Premion, IrO_*x*_ (Ir^0^ Free), and Hydrated IrO_*x*_ were investigated and are shown in Figures S14–S19. It is observed that the anodic prepeak for all catalysts shifts by around 0.09–0.11 V between 5 and 300 mV s^–1^. Furthermore, the peak separation was found to increase by around 55 mV for the Premion and 30 mV for the IrO_*x*_ (Ir^0^ Free), note that no cathodic peak could be observed for the Hydrated IrO_*x*_. The prepeak of hydrated iridium oxide is known to be dependent on both scan rate and electrode history.^44,53–55^ Specifically, it shifts positively after being held at low potentials,^53^ exhibits slow kinetics,^40,41^ and disappears entirely in alkaline electrolyte.^53^ Consequently, the prepeak has been associated with the coordination of anions within the oxide film.^40,41^ Based on the prepeak’s pH dependence and EQCM studies, it has been suggested by Birss et al. that coordination occurs on more hydrated sites, with more solution anions serving as charge-compensating species.^44^ During the cathodic sweep, anions and H^+^/H_2_O species are injected, leading to the formation of low-conductivity Ir^3+^ species. These anions and hydronium ions are subsequently ejected during the following anodic sweep. The analogous behavior observed in the Hydrated IrO_*x*_ prepeak and the Premion or IrO_*x*_ (Ir^0^ Free) prepeaks (A_1_/C_1_) indicate that a similar process occurs in both materials; however, it is noted that the onset potential is significantly lower in the Premion. This lower onset of the redox peaks is also noted in the A_2_/C_2_ peak compared to that of the Hydrated IrO_*x*_; it appears that all of the redox peaks are shifted to higher potentials in the hydrated material compared to the Premion.

It has been noted by Papakonstantinou et al.^45^ that an extra cathodic charge is present in the CV of the Premion after going to OER potentials which is associated with the deactivation of the catalyst. These same extra cathodic reductions were also observed in the CV (Figure S20) for the Premion, IrO_*x*_ (Ir^0^ Free) and possibly even the Hydrated IrO_*x*_ but at higher potentials. The authors attribute this extra cathodic charge to the removal of protons from bridging oxygens that form during the preceding OER. The shifting in potential of this extra cathodic reduction in the Hydrated iridium oxide compared with the Premion would suggest it is coupled to the redox peaks. The origin of this extra charge is investigated further through potentiodynamic XAS.

Prior to conducting activity measurements, the catalysts underwent conditioning by cycling the electrodes between 1.3 and 1.7 V_RHE_ at a rate of 50 mV s^–1^ for 35 cycles. The conditioning process resulted in a mass activity increase of 120% and 170% at 1.60 V_RHE_ for the Premion and IrO_*x*_ (Ir^0^ Free) catalysts, respectively (as shown in Figure S21 and Table S6). In contrast, both Hydrated IrO_*x*_ and Premion (800 °C) showed a loss in activity following the conditioning cycles. In the case of Hydrated IrO_*x*_, a substantial loss of catalyst during activation was evidenced by a reduction in CV area by 77% (Figure S22 and Table S7). The main redox peak in the CV remains the unchanged before and after conditioning; therefore, the loss in FE CV charge is presumed to be from dissolution of the catalyst rather than any restructuring. As a result, the preconditioned activity is used for subsequent activity comparisons, while the Premion (800 °C) shows a 13% activity loss at 1.7 V_RHE_ after conditioning. This minor loss may be due to the oxidation of contaminants during the first scan. A comparison of the geometric activities for the Premion, IrO_*x*_ (Ir^0^ Free), and Hydrated IrO_*x*_ with that of a bare Au current collector shows minimal contribution to the overall activity or CV (estimated to be around 0.2% of the geometric current of the Premion at 1.6 V_RHE_ Figure S23). Although, the Premion (800 °C) exhibited a significantly higher geometric activity than the bare Au, as mentioned above, distinguishable features in the CV for both the low- and high-loading electrodes attributed to iridium were masked by the Au current collector. The main changes that occur to the Premion and IrO_*x*_ (Ir^0^ Free) CVs during conditioning include an overall increase in the CV area, increasing by 30 and 59 C g^–1^, respectively. Similar to fuel cell testing,^56^ this activation occurs as protons and water are transported into the pores of the catalyst layer and embedded air expelled ensuring all iridium particles are in electrochemical contact. Additionally, a shift in the redox peak at around 0.3 V_RHE_ (designated A_1_ and C_1_ in Figure 6) was noted during conditioning. The peak shifts by around 20–50 mV to higher potentials on both the anodic and cathodic scans reflecting the equilibration of water and ions within the structure and the electrolyte.^44^

**Figure 6:**
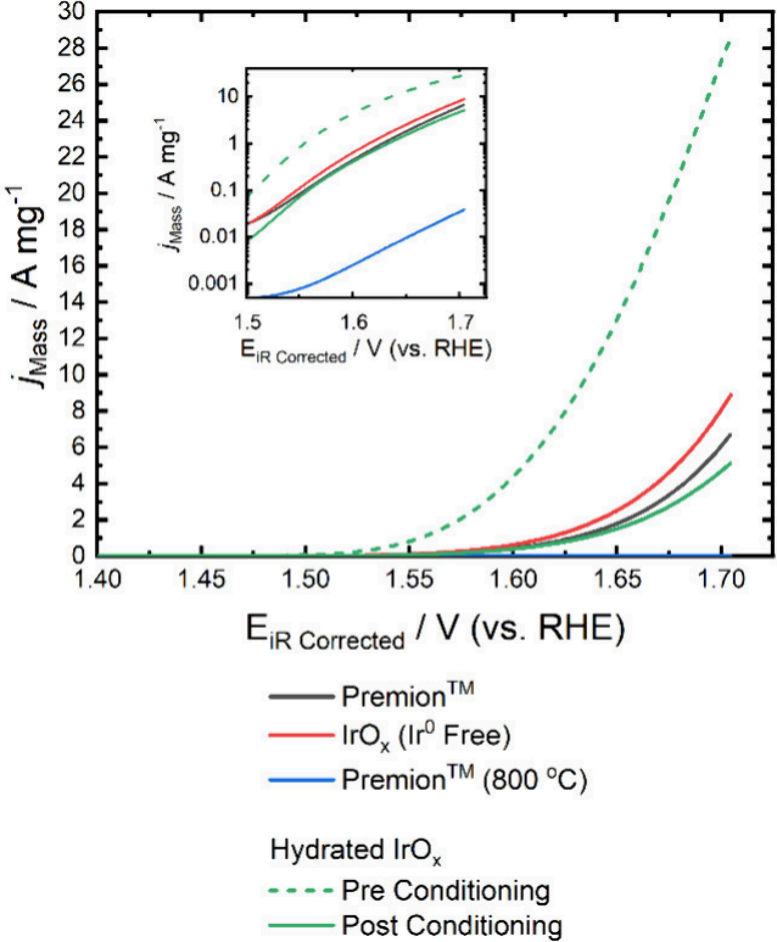
Mass-normalized linear sweep voltammetry of the low-loaded electrodes recoded at 0.01 V s^–1^ with an inset showing the Tafel plot. The linear sweep voltammograms (LSVs) of the Premion, IrO_*x*_ (Ir^0^ Free) and Premion (800 °C) are all measured postconditioning while the Hydrated IrO_*x*_ is measured preconditioning. The iridium metal loading for all of the LSVs is around 10 μg_Ir_ cm^–2^. The exact loadings of each electrode are in the Experimental Section.

The mass normalized activity curves for the four different materials are presented in Figure 6, with an inset showing the Tafel plot. It is observed that the activity of Hydrated IrO_*x*_ far exceeds that of the other materials, surpassing the activity of Premion by almost 10 times at 1.60 V_RHE_. However, as previously mentioned, Hydrated IrO_*x*_ shows a considerable loss of catalyst during conditioning. Between the first and second conditioning scans, Hydrated IrO_*x*_ lost 17.5% of its activity, indicating that approximately 8.75% of the current is due to dissolution of the catalyst, which corresponds to around 1.14 μg_Ir_ cm^–2^. This highlights the significant instability of the catalyst, rendering hydrous iridium oxides unsustainable for industrial use. To provide context, the maximum OER activity achieved was 4.03 A mg^–1^ at 1.60 V_RHE_. If this mass activity was maintained at technologically relevant Ir loadings (∼1600 μg_Ir_ cm^–2^),^57^ this would correspond to a geometric current density of ∼6.37 A cm^–2^_Geo_. We note, however, that this assumes linear scaling of activity with loading, which ignores mass-transport limitations (such as gas blinding or water flooding) and resistive losses (such as those caused by catalyst ionomer interactions, proton resistances, and electronic resistances). Moreover, our measurements were performed at room temperature, whereas Bernt et al.^57^ reported an activity of 5.5 A cm^–2^ at 1.6 V_RHE_ at 80 °C in an MEA configuration. This comparison, therefore, should be taken as a qualitative illustration of the unusually high intrinsic activity of hydrous IrO_*x*_ under ideal conditions, while in the other materials, IrO_*x*_ (Ir^0^ Free) shows a marginally higher activity than the standard Premion. This reflects the similar structures of the two materials and demonstrates that the removal of metallic iridium from the catalyst only accounts for negligible activity improvements as it only accounts for a small fraction of the material. In contrast, the Premion (800 °C) demonstrates significantly poorer performance, exhibiting only 1/177th of the mass activity of the Premion at 1.60 V_RHE_.

The Tafel plots for the materials are displayed in the inset of Figure 6 and slope values are shown in Table 6 (corresponding Tafel slope fits are in Figure S24). Two distinct regions are identifiable in the Premion, IrO_*x*_ (Ir^0^ Free), and Hydrated IrO_*x*_ materials. The first region is linear and occurs at low potentials (1.50 to 1.60 V_RHE_) while the second region is observed at higher potentials (>1.65 V_RHE_). However, the elevated potential region lacks a linear section that extends over one decade, complicating the accurate quantification of the Tafel slope, as mass transport effects cannot be ruled out. This is particularly evident for the Hydrated IrO_*x*_, which exhibits the highest activity and also shows significant nonlinearity at high potential. As a result, the Tafel slope is taken from the low-potential region. The Hydrated IrO_*x*_ shows the lowest Tafel slope at 48 mV dec^–1^, followed by IrO_*x*_ (Ir^0^ Free) at 63 mV dec^–1^ and Premion at 69 mV dec^–1^. The Premion (800 °C) appears to have one Tafel region extending the full potential range with a slope of 90 mV dec^–1^. It has a mass activity of around 0.0026 A mg^–1^ at 1.60 V_RHE_ which is relatively low compared with the other materials, indicating that it is unlikely to suffer from bulk mass transport effects. The Tafel slope appears to follow a trend of increasing as the materials transition from hydrated to rutile-type IrO_2_. This relationship between hydration and Tafel slope has been suggested previously, with values around 40 mV dec^–1^ typically associated with hydrous IrO_x,_ ^4,58–62^ and increases to over 60 mV dec^–1^ for samples with a higher degree of crystallinity.^62–66^ To investigate the kinetics in greater detail and disentangle the kinetic and bulk diffusion contributions, a loading study is required.

**Table 6:** Activity and Tafel Data Were Determined for Each of the Catalysts Tested Using the FE Technique

	Premion	IrO_*x*_ (Ir^0^ Free)	Premion (800 °C)	Hydrated IrO_*x*_ (preconditioning)
Mass Activity at 1.6_RHE_/A mg^–1^	0.46	0.64	0.0026	4.41 (4.03 OER Current)
Capacitance_1.15–1.30 VRHE_ Activity at 1.6 V_RHE_/A C^−1^	1.60	2.93	0.61	22.61
Tafel Slope/mV dec^–1^	68.5 ± 0.1	62.8 ± 0.2	89.5 ± 0.3	48.2 ± 0.2

The capacitance normalized activity curves are shown in Figure S25. The capacitance of rutile IrO_2_ is indicative of only the surface charging, as the bulk is inactive, while hydrous iridium oxides exhibit electrochemical redox behavior in both the bulk and surface. After normalizing the activity of the Premion (800 °C) by capacitance, a significant performance improvement is observed in comparison to the standard Premion, being only 2.6 times lower at 1.60 V_RHE_. As previously mentioned, there is substantial contribution from the Au current collector in the CV of the Premion (800 °C); thus, it is expected that the capacitance normalized activity may be even higher than depicted. Therefore, although there is a definite surface area effect, the activity of the hydrated materials is intrinsically higher.

### Potentiodynamic XAS

Dynamic changes in the catalyst were evaluated through the collection of potentiodynamic XAS data. This analysis was conducted with an approximate time resolution of 45 s during a 1 mV s^–1^ cyclic voltammogram, resulting in a potential resolution of 45 mV. The CV limits were selected to observe the formation of the low-conductivity Ir^3+^ species and the active species associated with the OER. Additionally, two upper potential limits (UPLs) were chosen to investigate the origin of the extra cathodic charge observed after going to the OER potentials.

High catalyst utilization was verified by comparing mass-normalized cyclic voltammograms obtained in the SPEC-XAS cell with those from the floating electrode, with both CVs demonstrating good agreement (refer to Figure S26). The electrodes were initially conditioned by cycling between 0 and 1.35 V_RHE_ at a rate of 200 mV s^–1^ for approximately 100 cycles, after which no further changes in the voltammogram were observed (final 200 mV s^–1^ and activity curves are shown in Figure S27). Confirmation of minimal lateral resistance of the electrode was achieved by periodically repositioning the spot where the XAS measurements were conducted, with no discrepancy observed between the different measurement positions.

The results obtained from the potentiodynamic XAS measurements are displayed in Figure 7 (with XANES and FT-EXAFS shown in Figures S28–S31), illustrating the mass normalized activity, d-hole count/oxidation state, and Ir–O distances as a function of potential. Full XANES and EXAFS fits are shown in Figures S32–S39 and Tables S8–S11 for each material. The responses of the Premion, IrO_*x*_ (Ir^0^ Free), and Hydrated IrO_*x*_ exhibit notably similar changes. Two primary regions are identified: a “plateau” region at low potentials, where there is no observable change in the XAS, and a “linear” region, where variations in the d-hole count/oxidation state and Ir–O distances occur in response to changes in the applied potential. In contrast, for the Premion (800 °C), no changes in the XAS signal are observed even during oxygen evolution. This behavior is attributed to the confinement of the reaction on rutile-type iridium oxides to the particle surface, resulting in a diminished surface signal due to the dominant contribution from the inactive bulk, which remains in the Ir^4+^ state. This is confirmed by calculating the surface percentage from the BET surface area and is found to only be around 1% surface (SI Note 1); therefore, even if all surface atoms are oxidized to Ir^5+^ during the OER, this would only cause an oxidation state increase of around 0.04, too low to be determined by XAS.

**Figure 7:**
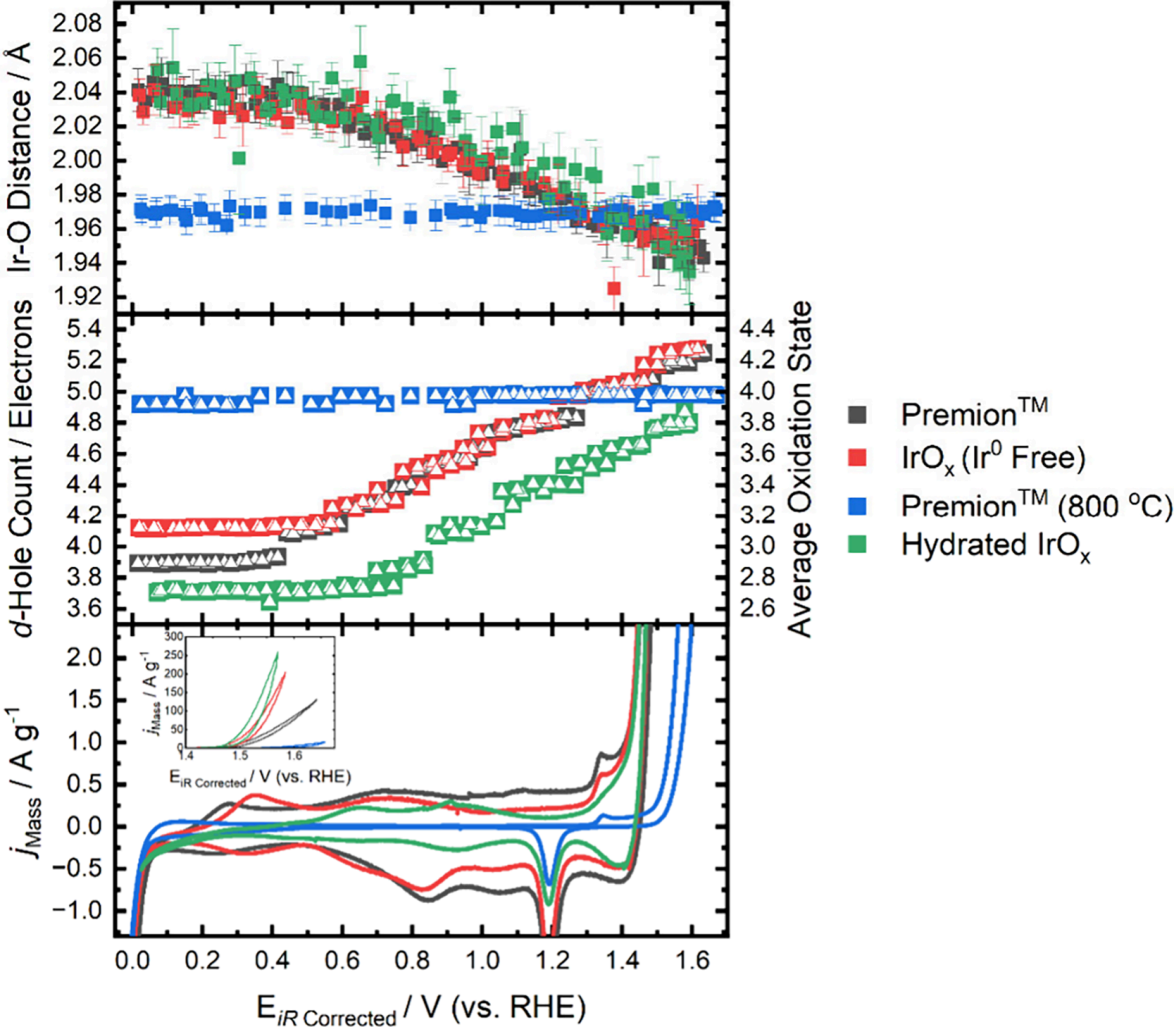
Oxidation states with the corresponding mass normalized cyclic voltammetry (0.001 V s^–1^) and the fitted iridium–oxygen distance during the CV. The iridium metal loadings determined by XRF of the electrodes prior to testing are 190 μg_Ir_ cm^–2^ for the Premion, 170 μg_Ir_ cm^–2^ for the IrO_*x*_ (Ir^0^ Free), 410 μg_Ir_ cm^–2^ for the Premion (800 °C) and 170 μg_Ir_ cm^–2^ for the Hydrated IrO_*x*_.

Similar trends to those shown in Figure 7 are also observed in the white line area and the full width at half-maximum (FWHM) (Figure S40). A strong correlation between the oxidation state and white line area of the Ir L_III_ edge has been documented in previous studies of iridium perovskites; however, factors such as spin-exchange interactions and covalent bonding character may contribute to an increase in the white line area.^21^ The FWHM indicates changes in the shape of the white line as a function of potential, revealing that the white line broadens as the potential becomes more positive. The intrinsic bandwidth is the primary determining factor behind the width of the white line, although it is also affected by core-hole lifetimes, final state lifetime effects, and instrument resolution.^67–70^ Furthermore, no significant drop in the white line area is observed at high potentials, which has previously been attributed to the loss of catalyst from the electrode.^71^ This observation reflects the use of low catalyst loadings (around 200 μg_Ir_ cm^–2^), resulting in thin catalyst layers that are not susceptible to beam-induced losses or delamination caused by high rates of bubble formation.

A plateau region is observed at low potentials and is accompanied by the CV charge in this region approaching zero. As previously noted, the voltammetry of the Hydrated IrO_*x*_ is characteristic of a hydrated iridium oxide, with the formation of a low-conductivity Ir^3+^ species resulting in a current decline toward zero. These observations are supported by findings indicating that the oxidation state of iridium is around 3. Plateau regions are also observed in the Premion and IrO_*x*_ (Ir^0^ Free) samples, where the current similarly approaches zero at low potentials, suggesting that these materials also form the low-conductivity Ir^3+^ species in sufficient quantities to effectively “switch off” the electrode. Differences in the extent and onset of Ir^3+^ coverage among the materials are evident, with Hydrated IrO_*x*_ exhibiting the highest proportion of Ir^3+^, followed by Premion and then IrO_*x*_ (Ir^0^ Free). The onset of the plateau aligns with the positions of the peaks observed in the voltammetry, where the Hydrated IrO_*x*_ begins at the highest potential, followed by IrO_*x*_ (Ir^0^ Free) and last, Premion. The relationship between the plateau onset and the voltammetry is shown clearly in Figure S41, where the plateau is observed to occur either before or during the prepeak (A_1_/C_1_). The shift of the plateau region to higher potentials indicates an improved stabilization of the Ir^3+^ species in hydrated iridium oxide, which is further evidenced by the shift in the A_2_/C_2_ peaks in the Hydrated IrO_*x*_ compared to the Premion and IrO_*x*_ (Ir^0^ Free) materials. As discussed above, the prepeak is proposed to arise from the oxidation of Ir^3+^ to Ir^4+^ species on more hydrated sites with increased adsorbed ions.^44^ However, this study found that the prepeak is not accompanied by a significant change in oxidation state detectable by XAS. Note, previous studies by both Hüppauff and Lengeler as well as Mo et al. also found no evidence of the prepeak in the potential vs oxidation state plot derived from *in situ* XAS of hydrated iridium oxide.^25,72^ Consequently, if the prepeak results from the oxidation of specific Ir^3+^ sites, it is suggested that it is confined to a small number of atoms with the signal masked by the contributions from overall response of the catalyst layer. It is possible that through more surface sensitive spectroscopies or by using higher resolution techniques, changes during this prepeak may be observed.

In the linear region, an increase in potential is associated with an increase in the oxidation state, while the Ir–O distance decreases correspondingly. Both the Premion and IrO_*x*_ (Ir^0^ Free) materials reach a maximum oxidation state of around 4.3, with an Ir–O distance of ∼1.94 Å as Ir^5+^ species are formed during the OER. In contrast, the Hydrated IrO_*x*_ reaches a maximum oxidation state of approximately 3.8 but exhibits a comparable Ir–O distance to that of the Premion and IrO_*x*_ (Ir^0^ Free) materials (∼1.94 Å). Note, that no plateau was observed at high potentials suggesting the oxidation state would continue to increase with increasing potential. Previous studies have demonstrated that Ir^5+^ species are formed at the onset of the OER; however, the average oxidation state would suggest this Ir^5+^ is not observed in the Hydrated IrO_*x*_. Two possible explanations can be used to elucidate the differences in the oxidizability between the Premion and Hydrated IrO_*x*_ materials. First, while fully hydrated materials, such as the Hydrated IrO_*x*_, exhibit different Tafel slopes, this should not be interpreted as direct evidence for a distinct rate-determining step alone. Changes in surface coverage of intermediates, potential dependent pathway switching, proton/electron transport limitations or mass transport and *iR* artifacts are all known to influence the apparent slopes.^73^ Within this broader context, one possibility is that the rate of Ir^4+^ turnover is slower than the Ir^5+^, resulting in an average oxidation state during the OER that lies closer to the Ir^4+^. Alternatively, it is possible that regions within Hydrated IrO_*x*_ remain in a reduced state throughout the measurements, leading to an overall average oxidation state that is lower and not representative of the active species. Given that the shape of the potential vs oxidation state curve of the Premion and Hydrated IrO_*x*_ are remarkably similar, albeit offset from each other, the latter explanation is considered more plausible. However, this observation does not clarify why the potential-dependent Ir–O distance of the Hydrated IrO_*x*_ displays variations similar to those of the Premion.

To gain a more detailed understanding of the linear region, the Ir–O distance can be plotted as a function of the average oxidation state (Figure 8). Previous studies have reported that the contraction of the Ir–O distance occurs in a variety of iridium perovskites materials, with a contraction of approximately 0.039 Å per oxidation state between Ir^4+^–Ir^6+^.^21^ In the present measurements, however, a greater contraction of around 0.070 Å per oxidation state is observed between Ir^3+^ and Ir^5+^ for Premion, IrO_*x*_ (Ir^0^ Free) and Hydrated IrO_*x*_. Note, the oxidation state of Premion (800 °C) does not change enough to have a strong correlation, but the region collected does follow the trend of the others, within error. A comparison of the data in Figure 8 to the iridium perovskites published by Choy et al. is shown in Figure S42.^21^ This divergence in slope is likely indicative of structural and bonding differences between the perovskites and amorphous iridium oxides, with the latter demonstrating a wider range of structural mobility. The consistent change in Ir–O bond length with the oxidation state across the materials confirms the structural similarities and suggests a universality in structural flexibility.

**Figure 8:**
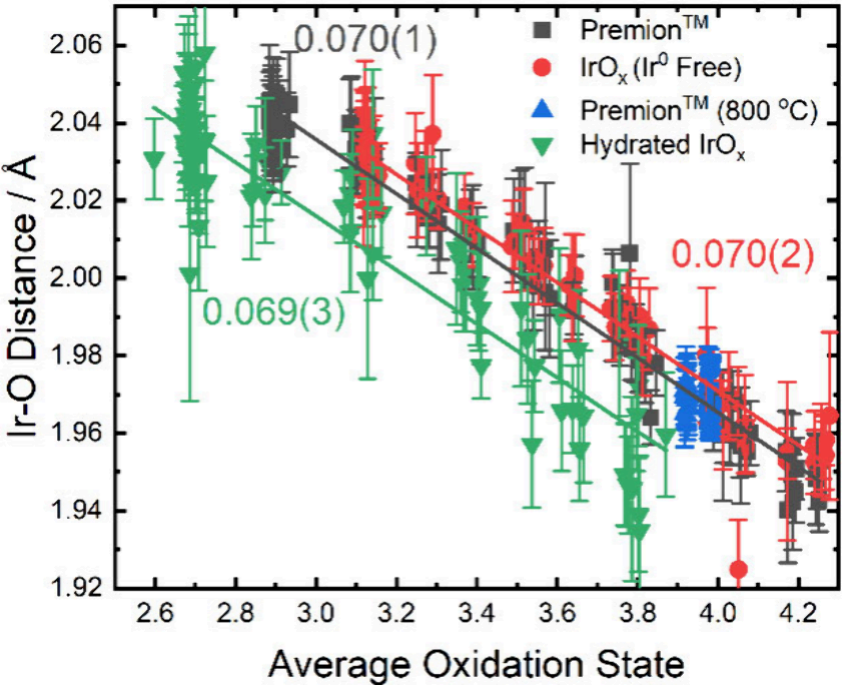
Correlation between the oxidation state and the Ir–O distance measured over a 0–1.65 V_RHE(*iR* Corrected)_ range for the Premion, IrO_*x*_ (Ir^0^ Free), Premion (800 °C) and Hydrated IrO_*x*_ catalysts. Gradients from the trendlines are provided for all but the Premion (800 °C).

In addition to the contraction of the Ir–O bond, the Ir–Ir bonds are also found to contract at higher potentials, as illustrated in Figures S43–S44. This observation is further corroborated by other studies using XAS and Pair Distribution Function (PDF) analysis of iridium oxide materials.^58,74^ The second shell Ir–Ir_1_ distance represents the edge-sharing iridium distance of the [IrO_6_] octahedra, while the third shell Ir–Ir_2_ distance corresponds to the length of corner sharing [IrO_6_] octahedra. A significant contraction of both corner- and edge-sharing Ir–Ir bonds occurs during the OER for the Premion and IrO_*x*_ (Ir^0^ Free). A plateau region is also discernible in the Ir–Ir distances for both the Premion and IrO_*x*_ (Ir^0^ Free) materials. The Premion (800 °C) remains constant at all potentials, close to the nominal values for the edge-sharing and corner-sharing distances in rutile iridium oxide, 3.16 Å and 3.56 Å, respectively. Interestingly, the amorphous samples show marginally shorter edge-sharing but longer corner-sharing distances.

Pittkoski et al.^74^ observed a peak broadening in their PDF data during the OER, which was attributed to the increased atomic mobility within the structure at elevated OER potentials. This observation is reflected in the Debye–Waller factors extracted from the EXAFS fits of the IrO_*x*_ materials, as shown in Figure S45. Under conditions of high oxygen evolution reaction, the structural disorder increases, emphasizing the flexibility of these materials. This change in structural order is reversible and is seen to plateau at potentials below around 0.8 V_RHE_. Above this potential, an increase in the disorder of the Ir–O path is noted. Interestingly, the disorder increases during the Ir^3+^/Ir^4+^ redox transition and reaches a plateau prior to the onset of the OER during the Ir^4+^/Ir^5+^ redox transitions. This suggests a structural reordering into the active OER material, which is characterized by a more disordered structure.

To investigate the additional cathodic charge observed in the CV after going to the OER potentials, two CVs were measured concurrently with a scan rate of 1 mV s^–1^. The first measurement was performed with the UPL set to 1.35 V_RHE_, just prior to the onset of the OER. The second measurement was conducted with the UPL set within the OER region, which has been the focus of the discussion so far. A comparison of the two UPLs is shown in Figures S46–S48 for the Premion, IrO_*x*_ (Ir^0^ Free), and Hydrated IrO_*x*_ catalysts. Previous studies utilizing EQCM techniques have indicated this additional cathodic charge is associated with an increase in weight, attributed to the protonation of surface bridging oxygens that undergo oxidation during the OER.^15,45^ It is suggested that one hydronium per electron is also injected into the iridium oxide. Oxidation of the bridging sites should be accompanied by an oxidation state change; however, no observable differences in the XAS have been detected between the two UPLs for any of the materials. It is thus proposed that the majority of the additional charge arises from non-Faradaic proton injection facilitated by the reduction of surface Ir^5+^ species. The corresponding Faradaic processes to the additional charge are sufficiently small as to be undetectable within the overall XAS signal. Notably, the Hydrated IrO_*x*_ catalyst has a positively shifted Ir^3+^/Ir^4+^ redox peak relative to the Premion and shows additional cathodic charge after reaching OER potentials. This cathodic feature is notably shifted compared to the Premion and does not overlap with the Ir^3+^/Ir^4+^ peak.

### Time-Resolved XAS During Potential Holds

To investigate the stability and reversibility of the Premion iridium oxide, time-resolved XAS measurements were conducted during various potential holds, as illustrated in Figure 9. The potential was held in the following sequence: OCP, 1.00 V_RHE_, 1.40 V_RHE_, 1.69 V_RHE_, 1.00 V_RHE_, 0.50 V_RHE_, and 0.00 V_RHE_. This sequence was designed to observe the stability during both the OER and the HER while comparing the reversibility post-OER.

**Figure 9:**
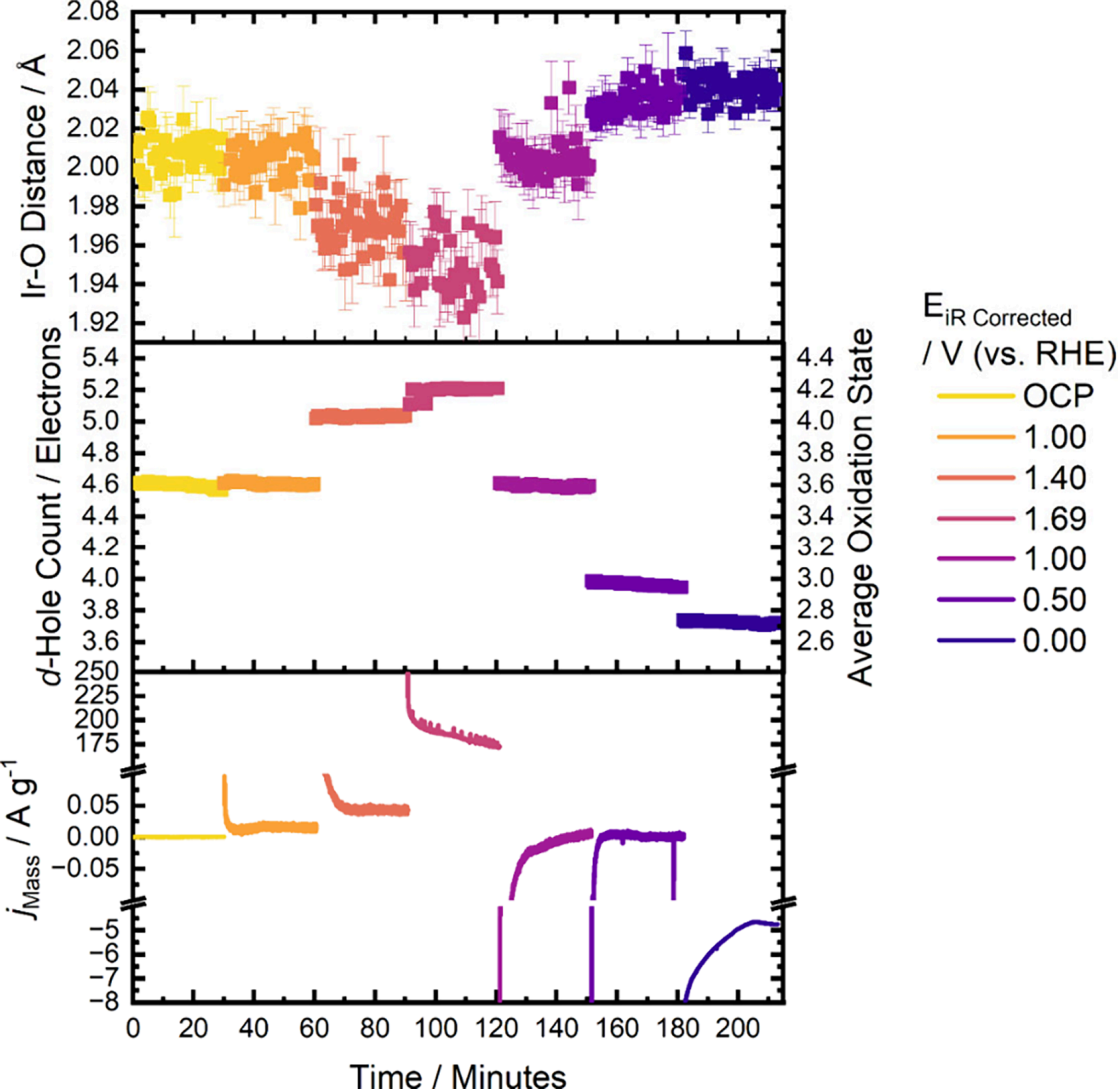
Time resolved oxidation state and Ir–O distance change for the Premion catalyst over the 30 min potential holds with corresponding mass normalized activity.

The kinetics of the iridium oxidation and reduction are beyond the measurement capabilities of the B18 beamline at Diamond Light Source, which can collect a full XAS spectrum in the time frame of tens of seconds; therefore, no intermediate species are observed in any of the XANES data, as a steady state was reached before the end of a single scan. Furthermore, a comparison between the averaged potential hold spectrum and the potentiodynamic data indicated excellent agreement (see Figure S49), suggesting that the iridium had achieved a steady state at the applied potential during potentiodynamic measurements.

There is some deviation at low potentials (∼0 V_RHE_) between the potential hold and potentiodynamic measurements, which was consistent for both the 1 and 0.1 M H_2_SO_4_ solutions (refer to Figure S50). Examination of the time-resolved oxidation state changes during the 30 min hold at 0 V_RHE_ demonstrated that this lower oxidation state was immediately stable and not the result of continual reduction of the iridium. It appears only to affect the oxidation state without affecting the Ir–O distance, giving evidence to the formation of metallic iridium. It can be estimated that the decrease in oxidation state could account for the formation of approximately 5.6% iridium metal. The increased formation of metallic iridium when stepping from 0.5 to 0.0 V_RHE_ in comparison to scanning down during a CV, may be attributed to the formation of sufficient Ir^3+^ during the CV. This accumulation of Ir^3+^ likely rendered the sample nonconductive, thereby protecting the iridium oxide from further reduction to metallic Ir^0^.

During the 1.69 V_RHE_ potential hold, a decay in current is observed over time, potentially due to deactivation of the Premion catalyst. However, no significant changes in the iridium oxidation state or the Ir–O distance are detected. Based on these XAS measurements, the catalyst appears to be considerably stable, with the observed deactivation likely resulting from catalyst reconstruction or loss from the electrode possibly due to substrate effects.^75^

The remarkable reversibility of the Premion catalyst is demonstrated by measurements at 1.0 V_RHE_ taken before and after exposure to the OER potentials, where the Ir–O distance and oxidation state show excellent agreement in both cases. The reversibility of the catalysts is further validated through the potentiodynamic measurements divided into the individual forward and reverse scans (Figures S51–S53). No hysteresis is observed in any of the iridium oxide samples, which is indicative of a high degree of reversibility of the materials.

A comparison between the XAS of the Premion in 1 and 0.1 M H_2_SO_4_ (Figure S50) reveals no significant deviations, indicating that the iridium oxide performance is not limited by proton transport at any of the concentrations tested. This finding is important as all of the protonation steps occurring during the cathodic sweep are accompanied by changes in the iridium oxidation state.

The Premion catalyst demonstrates high stability, despite containing partially hydrated sites. This hydration contributes to its catalytic effectiveness, enabling participation in a bulk OER pathway, in contrast to rutile catalysts, which only display surface activity. Furthermore, the Premion contains regions of crystallinity that may enhance its stability. As a result, it serves as a model catalyst, effectively balancing the activity and stability.

## Conclusions

We investigated a range of amorphous and crystalline iridium oxides to observe the electrochemical and structural changes that lead to improved activity and stability. By employing XAS techniques along with floating electrode electrochemical tests, we gained fundamental insights into these materials that will aid in future catalyst design. A hydrated iridium oxide was synthesized and demonstrates one of the highest activities reported for any OER catalyst, achieving an activity of 4.41 A mg^–1^ at 1.6 V_RHE_. However, this material is unstable, exhibiting a 17.5% activity loss after a single activity cycle. In contrast, the Premion and IrO_*x*_ (Ir^0^ Free) materials show lower activity compared to the Hydrated IrO_*x*_ but have significantly higher stability.

The catalysts were characterized by potentiodynamic and time-resolved *in situ* XAS. Significant changes to the catalyst structure were observed during the OER compared to *ex situ* measurements. All three materials, Premion, IrO_*x*_ (Ir^0^ Free) and Hydrated IrO_*x*_ exhibited similar potential vs oxidation state and Ir–O distance profiles. These profiles show a plateau region at low potentials where the catalyst becomes nonconductive, and a linear region where the oxidation state and Ir–O distance scale linearly with the applied potential. Fitting the EXAFS data revealed that both the Ir–O and Ir–Ir distances are contracting throughout the linear region as the potential is increased, accompanied by an increase in oxygen disorder prior to the catalyst reaching OER potentials.

The structural flexibility observed across all amorphous samples suggests a universal mechanism of redox-driven lattice contraction that can be exploited to enhance catalytic turnover. The position of the anodic prepeak was investigated, finding no significant features in the XAS data, where in the Premion, IrO_*x*_ (Ir^0^ Free) and Hydrated IrO_*x*_ catalysts, the plateau region started either prior to or during the anodic prepeak. The extra cathodic charge occurring during the reverse scan of the Premion after going to OER potentials was investigated. However, no changes in the XAS signal attributed to this extra charge are observed. It is thus proposed that the majority of the additional charge arises from non-Faradaic proton injection facilitated by the reduction of surface Ir^5+^ species.

The stability of the Premion was evaluated using time-resolved XAS. Both after being held at the OER potentials for 30 min and after being cycled up to the OER potentials, the Premion exhibited remarkable stability. This reversibility, even under prolonged electrochemical stress, exemplifies the robustness of partially hydrated amorphous IrO_*x*_ as a practical OER catalyst. In conclusion, our findings established the Premion as a model catalyst for the OER as it combines characteristics of both hydrated and rutile iridium oxide, achieving an impressive balance between activity and stability. These insights provide a framework for the rational design of next-generation iridium-based catalysts that optimize hydration, crystallinity, and structural adaptability to meet demands of scalable water electrolysis.

Future work should aim to evaluate the stability of Premion iridium oxide under industrially relevant conditions by employing truly *operando* XAS within a membrane electrode assembly (MEA) setup at elevated temperatures and pressures. Additionally, exploration of “Premion-like” catalysts and mixed metal iridium systems could offer pathways to further enhance the unique activity/stability balance observed in Premion, while potentially reducing iridium content and improving scalability. Finally, new experimental approaches are needed to directly measure the Ir^5+^ species during the OER, as this could yield critical insights into the electronic structure and mechanistic role of the active sites.

## Supplementary Material



## Data Availability

The experimental data collected for this manuscript are accessible through Diamond for proposals, NT35671-1, SP10915-1, SP30590-1, SP33009-1.

## Notes

EXAFS data, TGA , STEM, XANES data, calibration of d-band hole count, comparison of the minimum in the second derivative, calculating surface percentage, normalized Ir L_III_ edge, variation of the first shell Ir–O distance with oxidation state, CVs, electrochemical analysis, mass activity, Tafel plots, XAS, FWHM of IR L_III_ edge, Ir–O bond length contraction, oxidation state data, and operando experimental data along with all of the fitting data (PDF)

## ∇

Department of Materials, University of Oxford, Parks Road, OX1 3PH Oxford, U.K
