# Supplementary material for: Role of Hydration in Uncovering the OER Activity of Amorphous Iridium Oxide Electrocatalysts
Source: ACS Catal. 2025 Dec 19;16(1):270–84. doi: 10.1021/acscatal.5c05765 (PMC12771689; doi:10.1021/acscatal.5c05765)
Supplement: Supplementary file 1 [file cs5c05765_si_001.pdf]

Supporting Information

The role of hydration in uncovering the OER activity of amorphous iridium oxide electrocatalysts.

Connor Sherwin[a, b, c], Veronica Celorrio[b] *, Alessandro Difilippo[c], Katie Rigg[c], Mark Clapp[c], Armando Ibraliu[b], Luke Luisman[c], Thomas Wakelin[a,b], Amber Watson[a], Nikolay Zhelev[a], Lucy Mcleod[c], Christopher M. Zalitis[c] *, Andrea E. Russell[a] *

[a] Department of Chemistry, University of Southampton, Southampton, SO17 1BJ (United Kingdom)

[b] Diamond Light Source Ltd, Didcot, OX11 0DE (United Kingdom)

[c] Johnson Matthey Technology Centre, Reading, RG4 9NH (United Kingdom)

*Corresponding authors: Veronica.Celorrio@diamond.ac.uk, Chris.Zalitis@matthey.com, A.E.Russell@soton.ac.uk.

Supporting Figures and Tables

Table of Contents

EXPERIMENTAL 2

EX SITU CHARACTERISATION 3

ELECTROCHEMICAL PERFORMANCE 18

POTENTIODYNAMIC XAS 29

REFERENCES 65

EXPERIMENTAL

Table S1 EXAFS model used for fitting iridium oxide.

Scattering Path	N	σ^2	ΔR	R_{eff} / Å
Ir_{abs}-O_1-Ir_{abs}	N_{O1}	SS_{O1}	ΔR_{O1}	1.9604
Ir_{abs}-Ir_1-Ir_{abs}	2	SS_{Ir1}	ΔR_{Ir1}	3.1586
Ir_{abs}-Ir_2-Ir_{abs}	8	SS_{Ir2}	ΔR_{Ir2}	3.5556
Ir_{abs}-Ir_2-O_1_Ir_{abs}	8	SS_{O1}+(0.5*SS_{Ir2})	ΔR_{O1}+(0.5*ΔR_{Ir2})	3.7574
Ir_{abs}-O_1-Ir_{abs}- O_1-Ir_{abs}	2	SS_{O1}*2	ΔR_{O1}*2	3.9208

EX SITU CHARACTERISATION

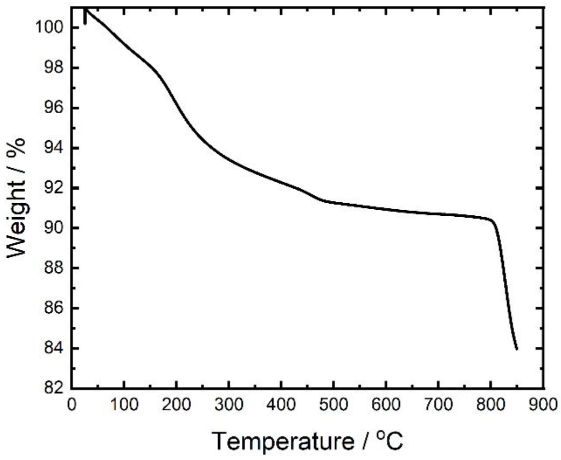

Figure S1 Thermogravimetric analysis of the Premion™ IrO_x measured with a 5 °C ramp rate from 25 °C up to 850 °C to determine the water content of the material. Collected on a TGA 5500 (Discovery TGA 5500 – TA Instruments Thermal Analyzers) under a N_2 flow at 100 ml min^{-1}.

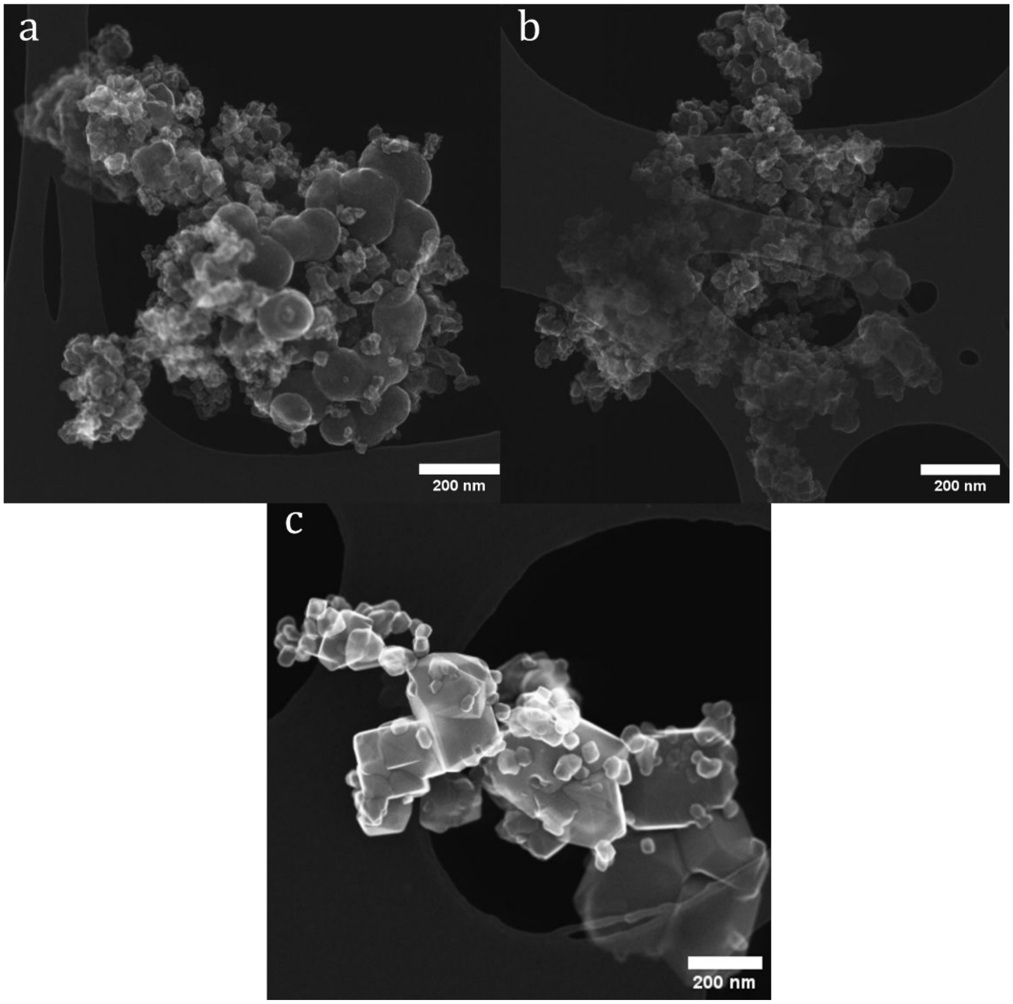

Figure S2 Scanning transmission electron microscopy images of a) Premion™ iridium oxide, b) the IrO_x (Ir^0 Free) and c) Premion™ (800 °C). The Premion™ shows the large iridium metal particles (approximately 83 ± 17 nm) surrounded by clusters of the amorphous IrO_x (approximately 11 ± 3 nm particles) while the IrO_x (Ir^0 Free) shows only the presence of the IrO_x. Collected on a JEM 2800 at a voltage of 200 kV, C2 apertured (um) 70 and 40.

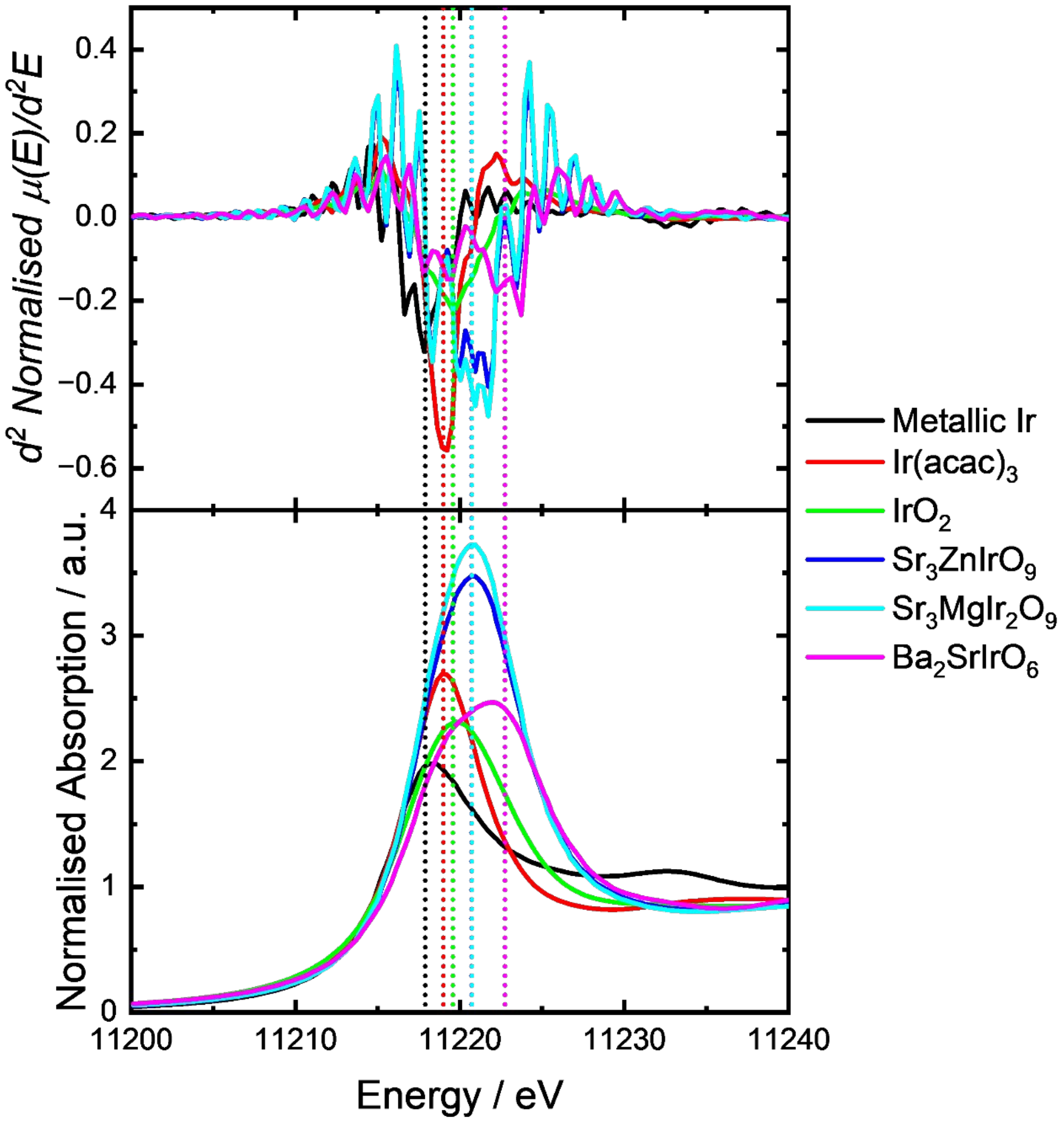

Figure S3 Normalized Ir L_{III} edge XANES spectra of six standards with oxidation states ranging from Ir^{3+} to Ir^{6+} along with the second derivates of the XANES. Using the minimum in the second derivative gives a more accurate determination of the edge position by probing the 2p – 5d e_g transition selectively. How the determined edge position correlated between the normalized XANES and second derivative spectra is shown by the dotted lines.

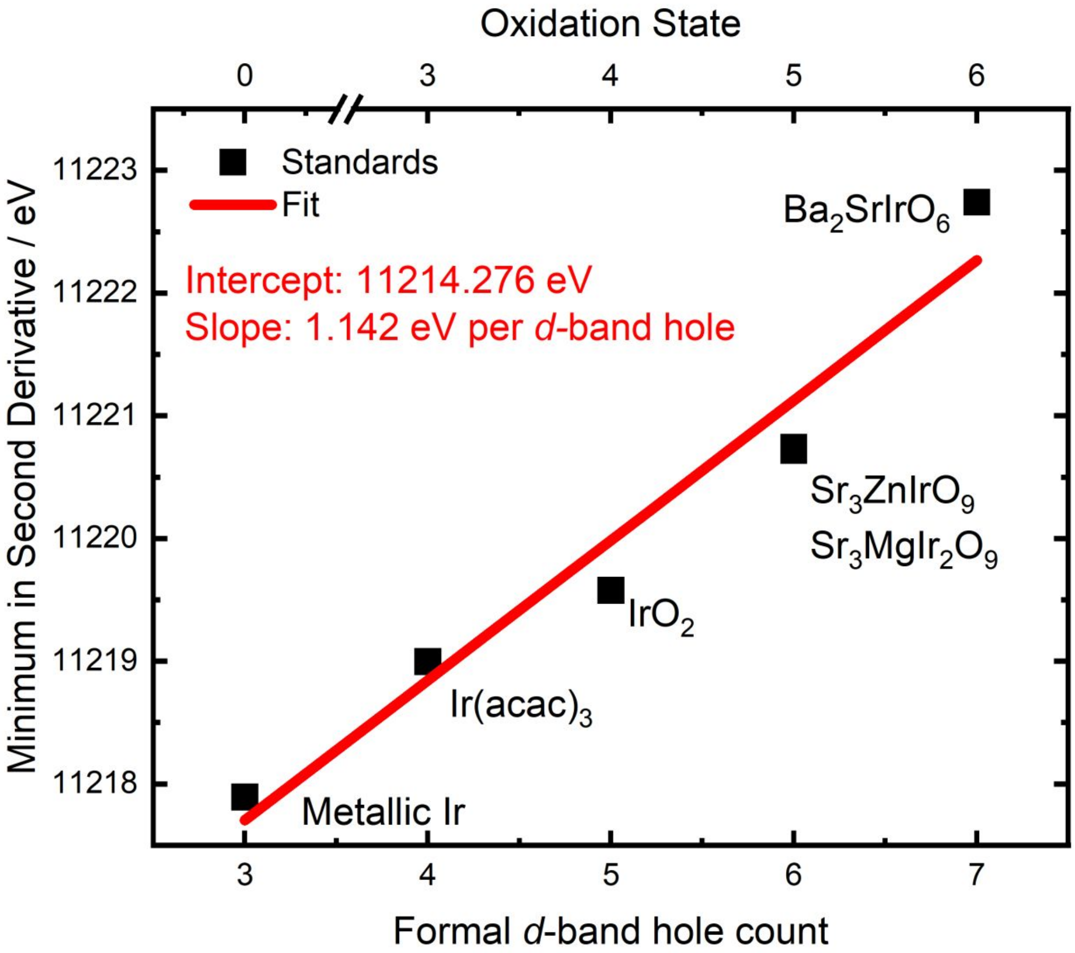

Figure S4 Calibration of d-band hole count to the minimum in the second derivative for standards of iridium L_{III} edge XANES spectra.

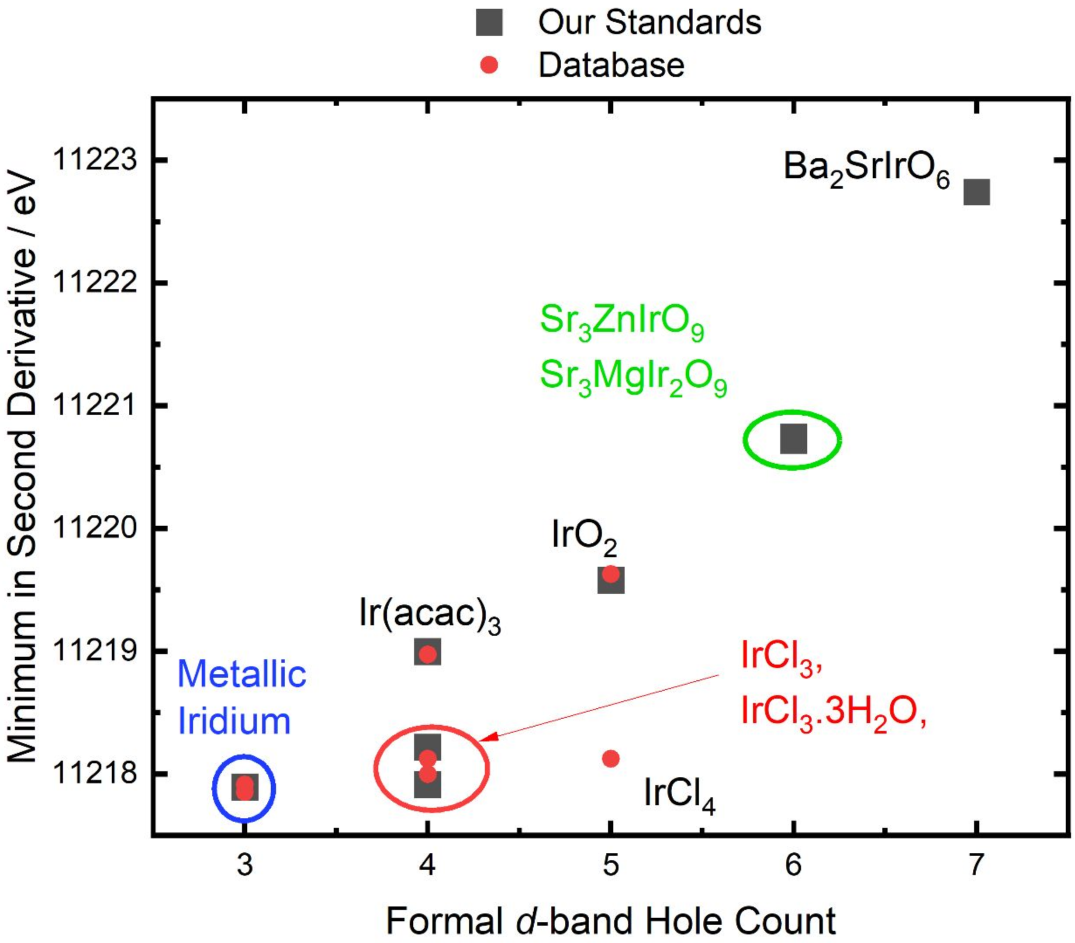

Figure S5 Comparison of the minimum in the second derivative for various standards, including standards from the Materials Data Repository XAFS Database.[1-8] Iridium chloride standards were included in the plot to demonstrate the discrepancy in the edge calibration when they are included, therefore Ir(acac)$_3$ was used as the Ir^{3+} standard in all edge calibrations instead of $IrCl_3$. It is believed that the reduced covalency of the Ir-Cl bond causes the edge position to be shifted. This has been found previously by Tensi *et al*.[9]

Note 1 Calculating surface percentage from BET surface area

The Fraction of iridium atoms at the surface of the Premion™ (800 °C) was estimated by degerming the total number of unit cells of iridium oxide assuming a density, ρ, of 11.66 g cm^{-3}, unit cell values of α=4.51, b=4.51, c=3.16 Å and thus calculating the unit cell volume, $V_{Unit\ Cell}$.[10]

$$\boldsymbol{Total\ No.\ of\ Unit\ Cells = \frac{1}{\rho * V_{Unit\ Cell}}}$$

Then the total number of surface unit cells are calculated using the BET surface area, $SA_{BET,}$ in Table 1 and the unit cell values states above.

$$\boldsymbol{Total\ No.\ of\ Surface\ Unit\ Cells = \left(\frac{SA_{BET}}{\alpha * b} + \frac{SA_{BET}}{\alpha * c}\right) * 0.5}$$

The percentage of surface cells is then calculated using:

$$\%\ \boldsymbol{Surface\ Cells = \frac{Total\ No.\ of\ Surface\ Unit\ Cells}{Total\ No.\ of\ Unit\ Cells} * 100}$$

This calculation assumes the density, unit cell parameters and a stoichiometric amount of IrO_2. This is most true for the Premion™ (800 °C) and is therefore the only time where we have applied it. To demonstrate the inactivity of bulk IrO_2 it's reflection in the XAS data. Using a BET surface of 1.98 m^2 g^{-1} gives a percentage of surface of around 1% for the Premion™ (800 °C).

Table S2 Fitted XANES parameters for the IrO_x Pellets

Parameter	Premion™	IrO_x (Ir^0 Free)	Premion™ (800 °C)
Area	18.3	17.6	16.2
FWHM	5.8	6.1	6.3
Minimum in Second Derivative	1129.72	11219.77	11219.74
Oxidation State	3.77	3.81	3.79
d hole count	4.77	4.81	4.79

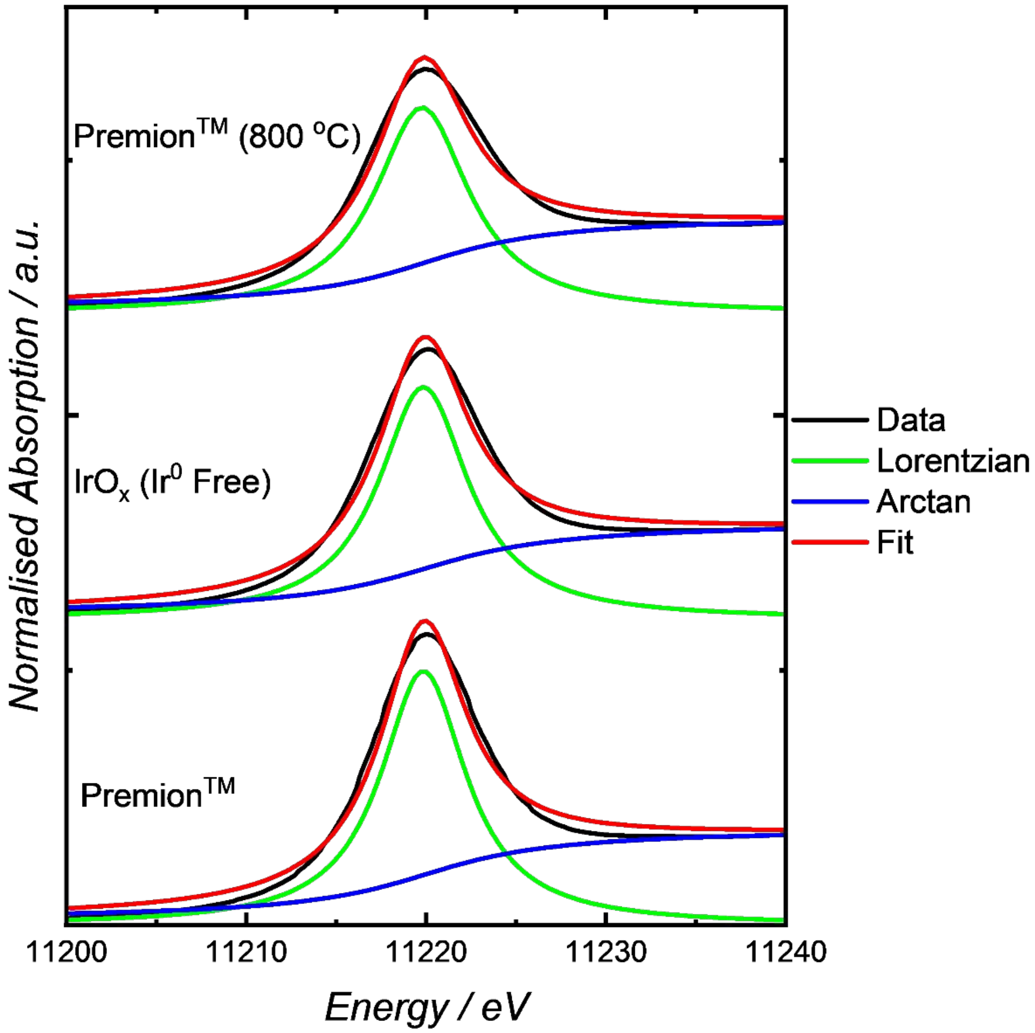

Figure S6 Normalized Ir L_{III} edge for the different IrO_x pellets with the background fitted using an arctangent function and the white line fitted using a Lorentzian function. This fitting was used to extract the edge positions, areas, FWHMs shown in Table S2.

Table S3 Fitted XANES parameters for the IrO_x electrodes.

Parameter	Premion™	IrO_x (Ir^0 Free)	Premion™ (800 °C)	**Hydrated IrO_x**
Area	20.2	20.3	21.3	18.8
FWHM	6.1	5.9	6.5	6.1
Minimum in Second Derivative	11219.28	11219.44	11219.82	11219.21
Oxidation State	3.39	3.52	3.86	3.32
d hole count	4.39	4.52	4.86	4.32

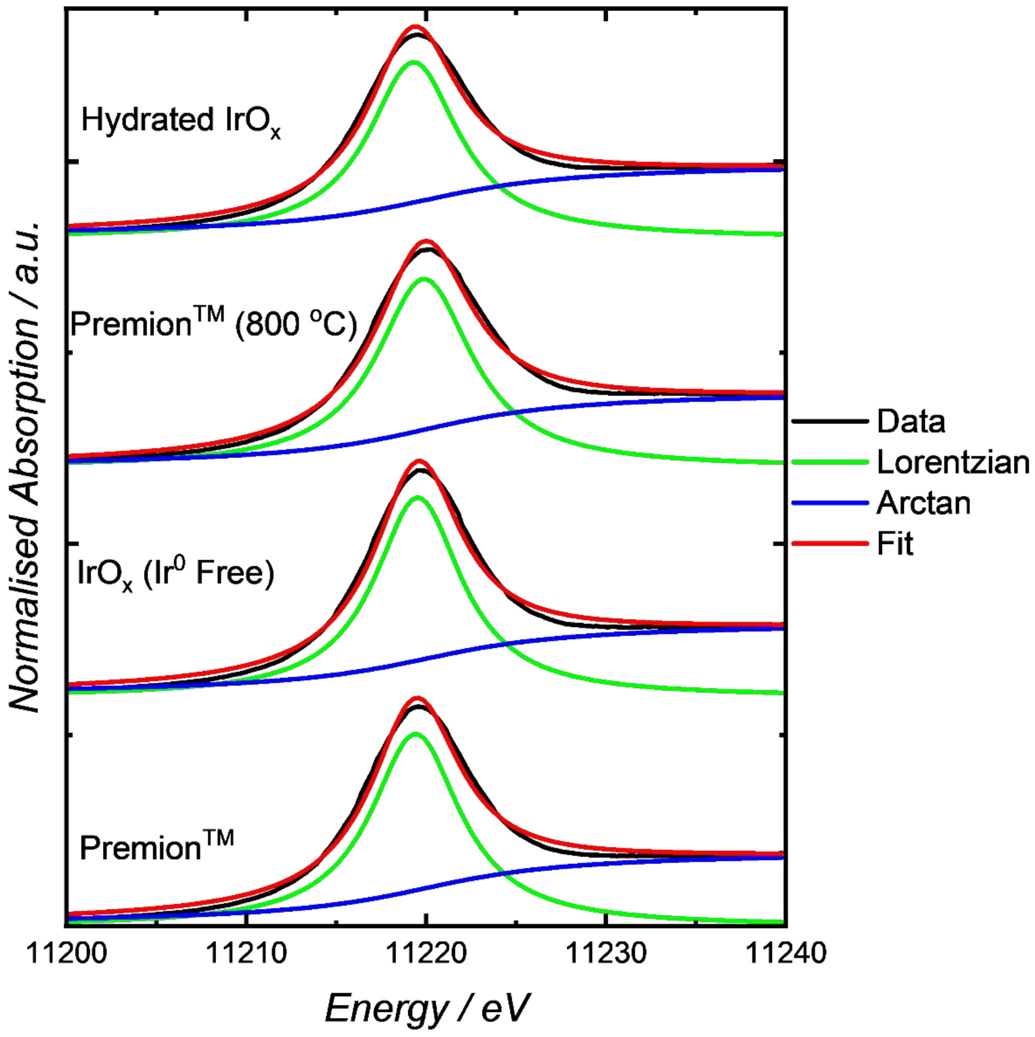

Figure S7 Normalized Ir L_{III} edge for the different IrO_x electrodes with the background fitted using an arctangent function and the white line fitted using a Lorentzian function. This fitting was used to extract the edge positions, areas, FWHMs shown in Table S3.

Table S4 Fitted EXAFS parameters for the IrO_x pellets.*

Parameter	Premion™	IrO_x (Ir^0 Free)	Premion™ (800 °C)
Energy Shift	17 ± 1.0	17 ± 1.0	16.9 ± 0.9
N_{O1}	7.2 ± 0.7	7.0 ± 0.6	5.7 ± 0.4
Ir_{abs}-O_1 / Å	1.982 ± 0.008	1.981 ± 0.006	1.975 ± 0.004
Ir_{abs}-Ir_1 / Å	3.08 ± 0.02	3.10 ± 0.02	3.138 ± 0.008
Ir_{abs}-Ir_2 / Å	3.8 ± 0.1	3.78 ± 0.08	3.546 ± 0.006
SS_{O1} / $Å^2$	0.007 ± 0.001	0.0070 ± 0.0009	0.0016 ± 0.0005
SS_{Ir1} / $Å^2$	0.007 ± 0.002	0.007 ± 0.001	0.0014 ± 0.0006
SS_{Ir2} / $Å^2$	0.03 ± 0.02	0.02 ± 0.01	0.0029 ± 0.0004

*The amplitude reduction factor was fixed at 0.78 for all fitting, calculated from fitting a crystalline iridium oxide. Fitting was performed in r space between 1- 4 Å, a fitting *k*-weight of 3, a hanning window with a tampering of 1 and a maximum *k* of 15.3 $Å^{-1}$.

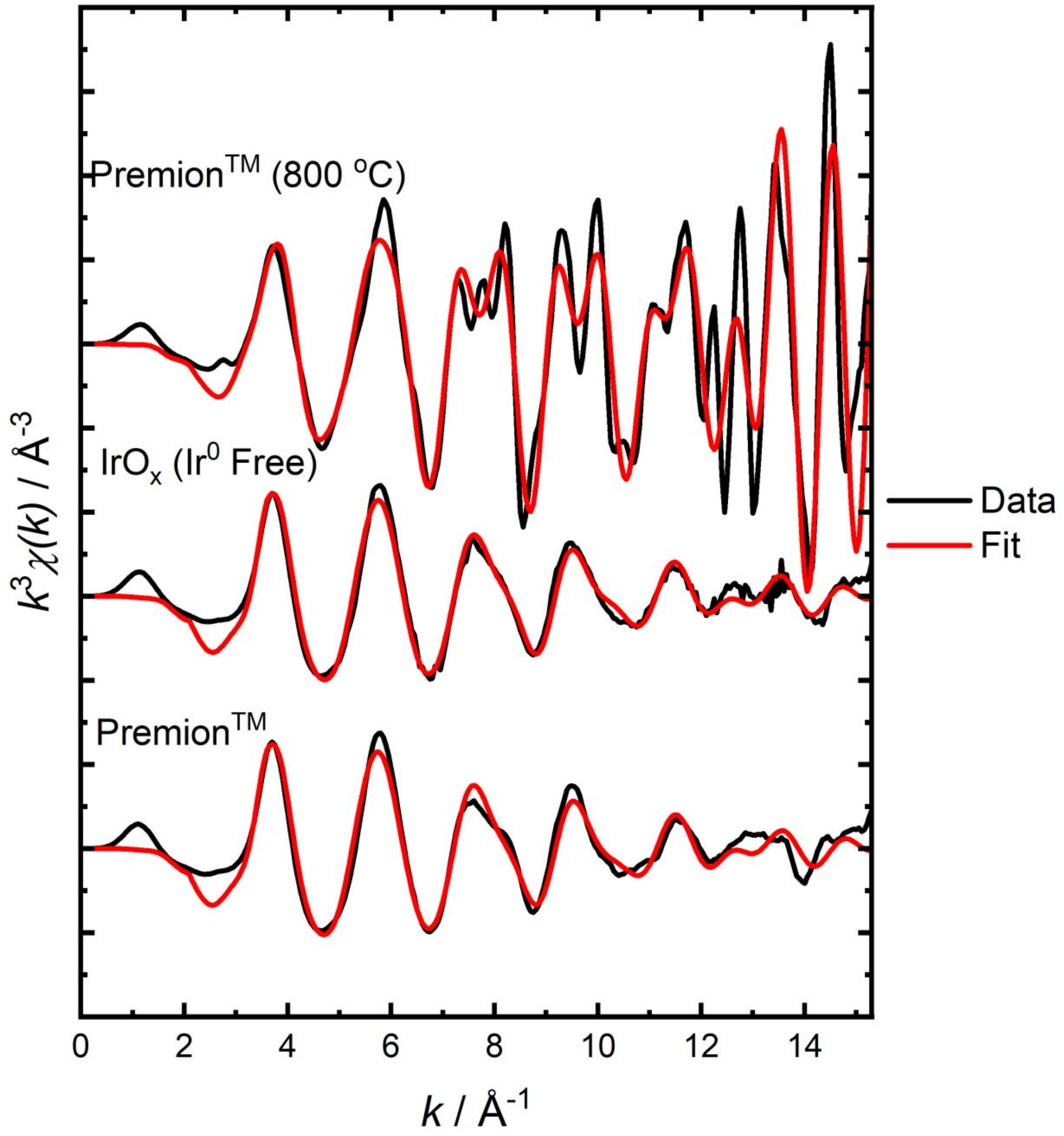

Figure S8 k^3-weighted $\chi(k)$ EXAFS fits of the IrO_x pellets fit using the model in Table S1 and the fitted parameters shown in Table S4. Fitting was performed in r space between 1- 4 Å, a fitting k-weight of 3, a hanning window with a tampering of 1 and a maximum k of 15.3 Å^{-1}.

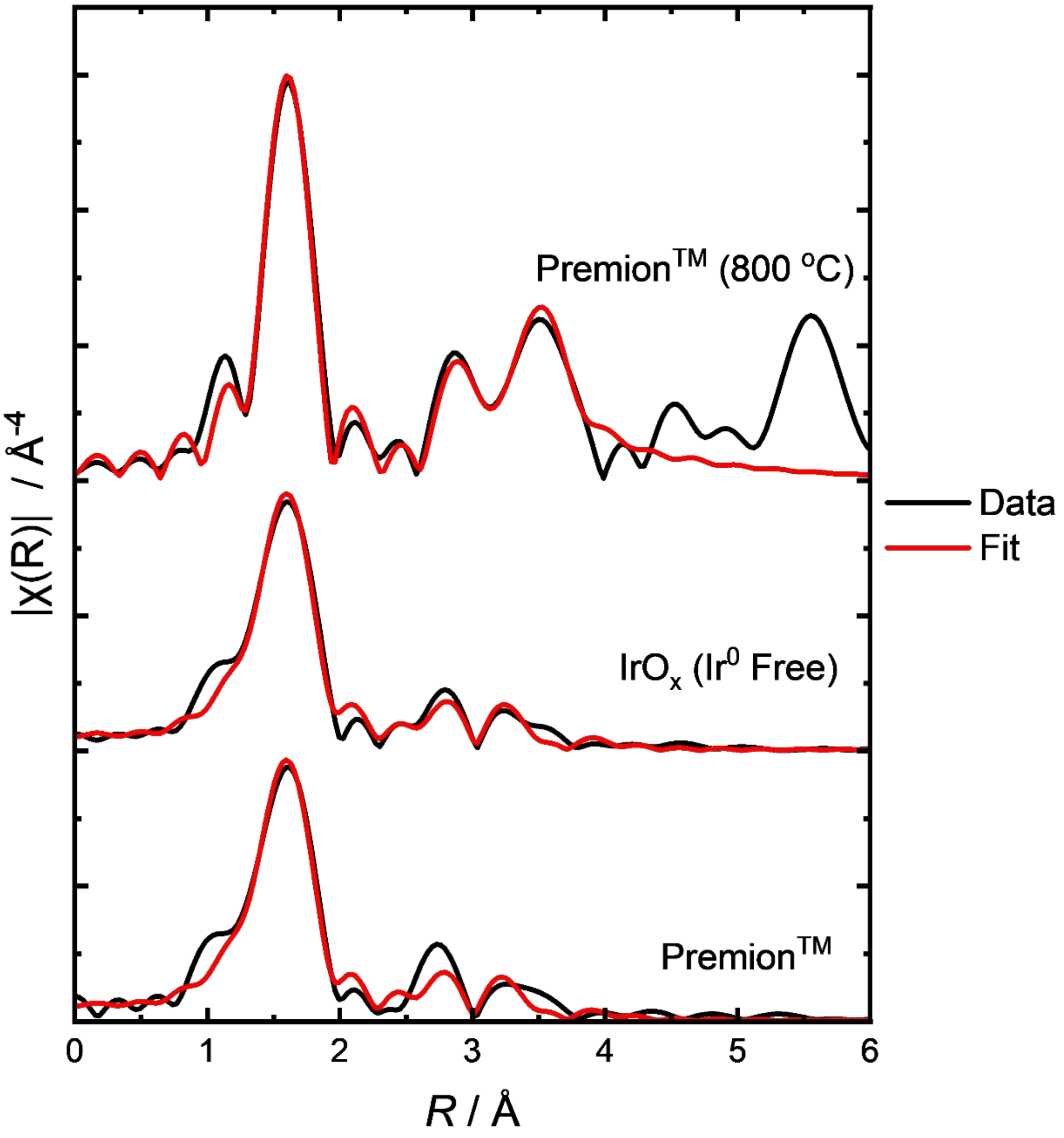

Figure S9 k^3-weighted Fourier transform EXAFS fits of the IrO_x pellets fit using the model in Table S1 and the fitted parameters shown in Table S4. Fitting was performed in r space between 1- 4 Å, a fitting k-weight of 3, a hanning window with a tampering of 1 and a maximum k of 15.3 $Å^{-1}$.

Table S5 Fitted EXAFS parameters for the IrO_x electrodes.*

Parameter	Premion™	IrO_x (Ir^0 Free)	Premion™ (800 °C)	Hydrated IrO_x
Energy Shift	16 ± 2.0	17 ± 1.0	17 ± 1.0	17 ± 1.0
N_{O1}	7 ± 1	7.1 ± 0.9	6.0 ± 0.7	6.4 ± 0.9
Ir_{abs}-O_1 / Å	2.00 ± 0.01	2.002 ± 0.009	1.970 ± 0.008	2.014 ± 0.009
Ir_{abs}-$Ir^{\cdot}_{1}$ / Å	3.48 ± 0.05	3.13 ± 0.03	3.15 ± 0.02	3.12 ± 0.02
Ir_{abs}-Ir_2 / Å	3.67 ± 0.08	3.8 ± 0.1	3.54 ± 0.02	3.8 ± 0.1
SS_{O1} / $Å^2$	0.005 ± 0.002	0.005 ± 0.001	0.001 ± 0.001	0.004 ± 0.001
SS_{Ir1} / $Å^2$	-0.001 ± 0.005	0.005 ± 0.003	-0.002 ± 0.002	0.004 ± 0.003
SS_{Ir2} / $Å^2$	0.01 ± 0.01	0.03 ± 0.02	0.003 ± 0.002	0.03 ± 0.02

*The amplitude reduction factor was fixed at 0.78 for all fitting, calculated from fitting a crystalline iridium oxide. Fitting was performed in r space between 1- 4 Å, a fitting *k*-weight of 3, a hanning window with a tampering of 1 and a maximum *k* of 12 $Å^{-1}$.

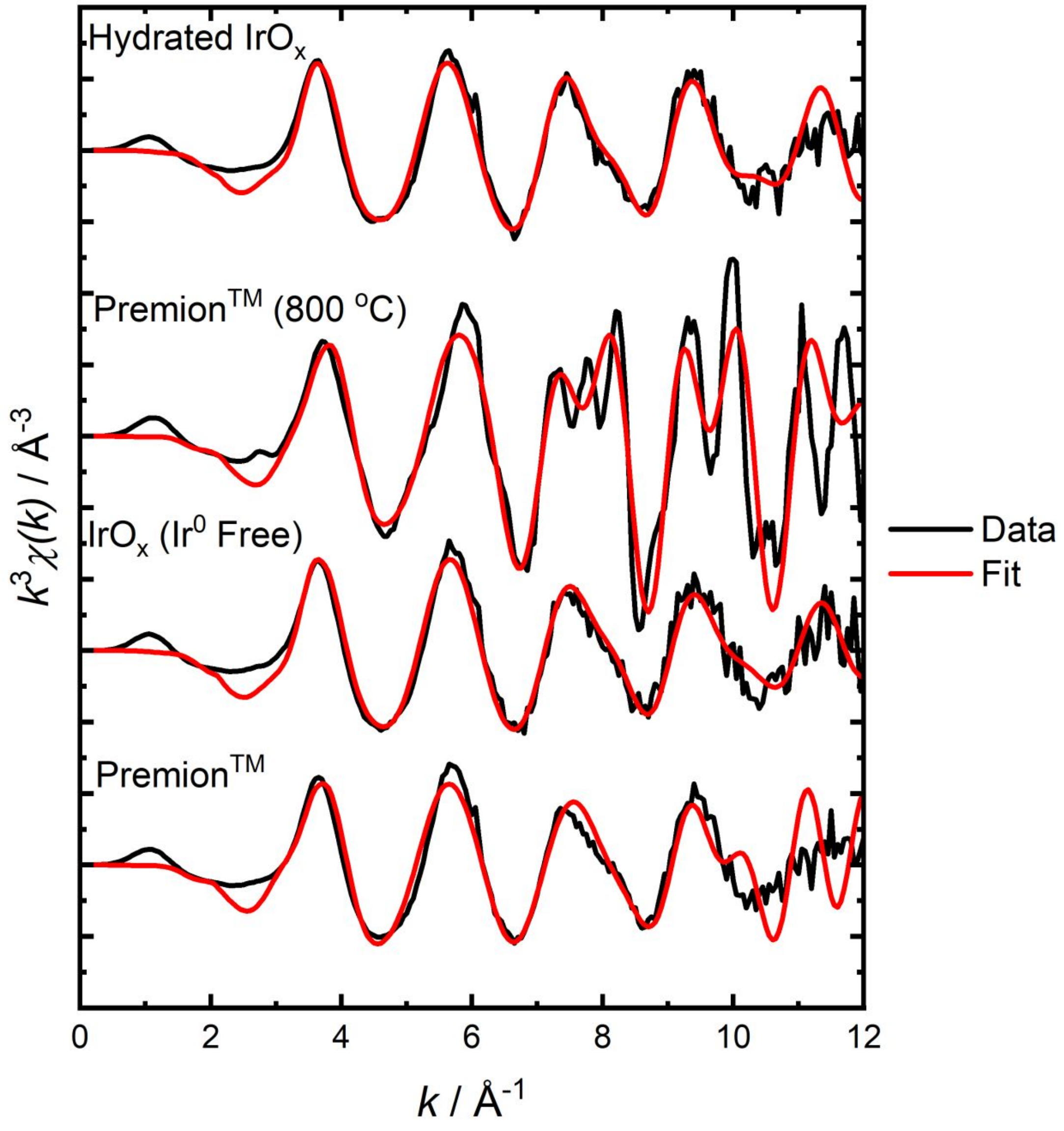

Figure S10 k^3-weighted $\chi(k)$ EXAFS fits of the IrO_x electrodes fit using the model in Table S1 and the fitted parameters shown in Table S5. Fitting was performed in r space between 1- 4 Å, a fitting k-weight of 3, a hanning window with a tampering of 1 and a maximum k of 12 Å^{-1}.

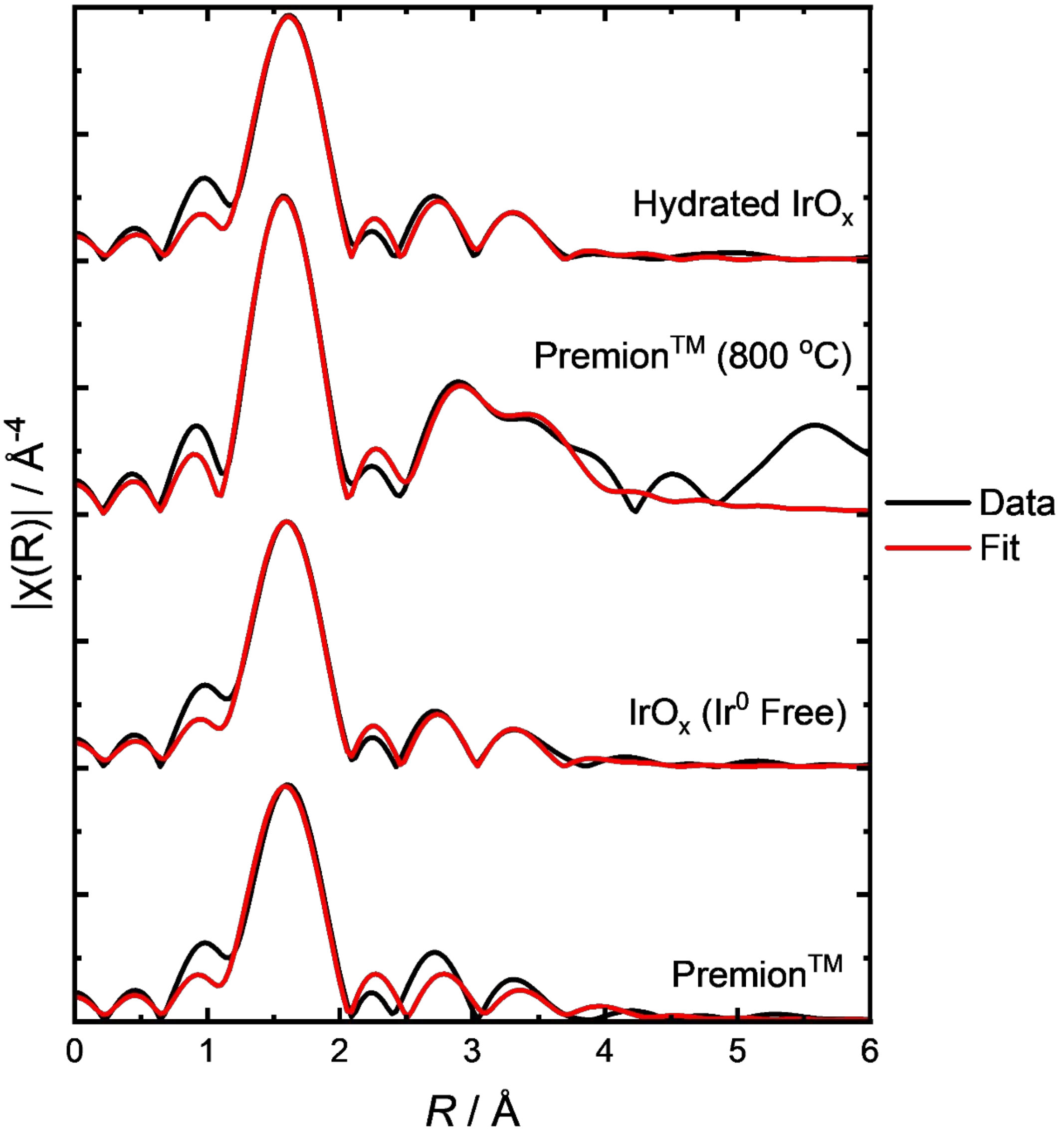

Figure S11 k^3-weighted Fourier transform EXAFS fits of the IrO_x electrodes fit using the model in Table S1 and the fitted parameters shown in Table S5. Fitting was performed in r space between 1- 4 Å, a fitting *k*-weight of 3, a hanning window with a tampering of 1 and a maximum *k* of 12 $Å^{-1}$.

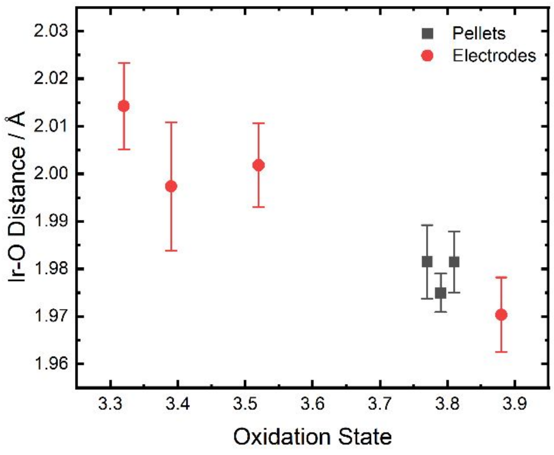

Figure S12 Variation of the first shell Ir-O distance with oxidation state. For the pellets, there is not much variation in the oxidation state or the Ir-O distance while for the electrodes, some reduction / oxidation has occurred during the electrode making process which has given with varying oxidation states and metal – oxygen distances.

ELECTROCHEMICAL PERFORMANCE

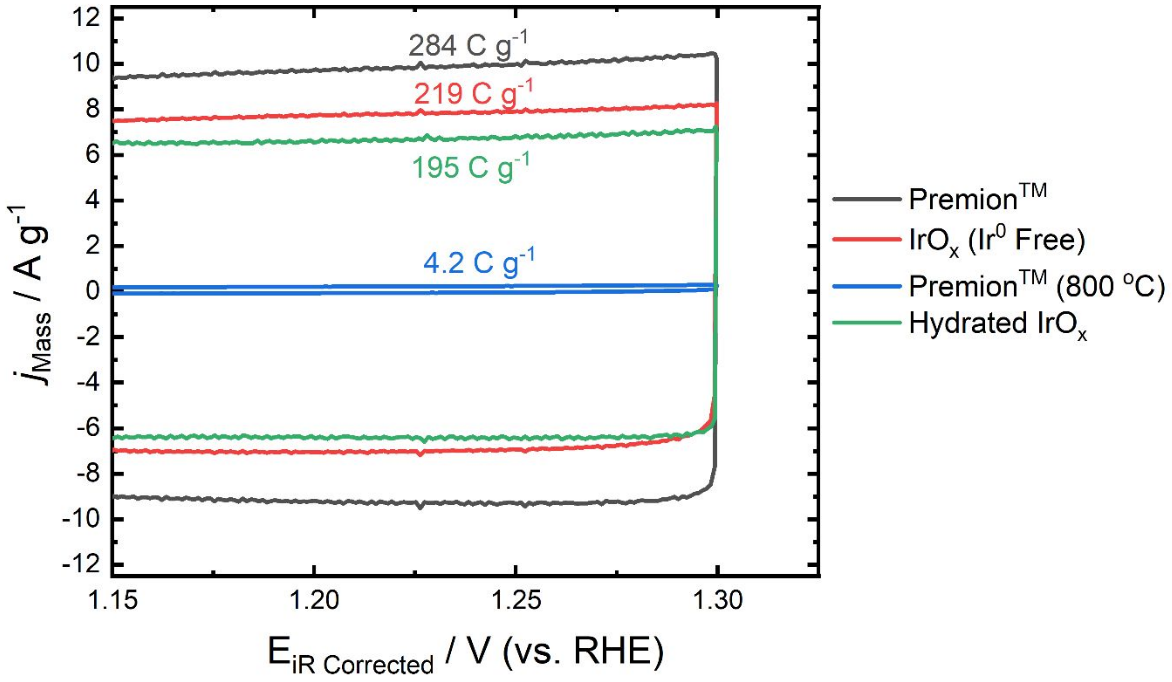

Figure S13 Comparison of CVs in the region used for calculating the mass-normalised capacitive charges. Measured by FE technique in N_2 purged 1 M H_2SO_4 at 10 mV s^{-1} and calculated in the potential range 1.15-1.30 V_{RHE}. Post-conditioned CVs were used for the Premion™, IrO_x (Ir^0 Free) and Premion™ (800 °C) while the pre-conditioned CV was used for the Hydrated IrO_x.

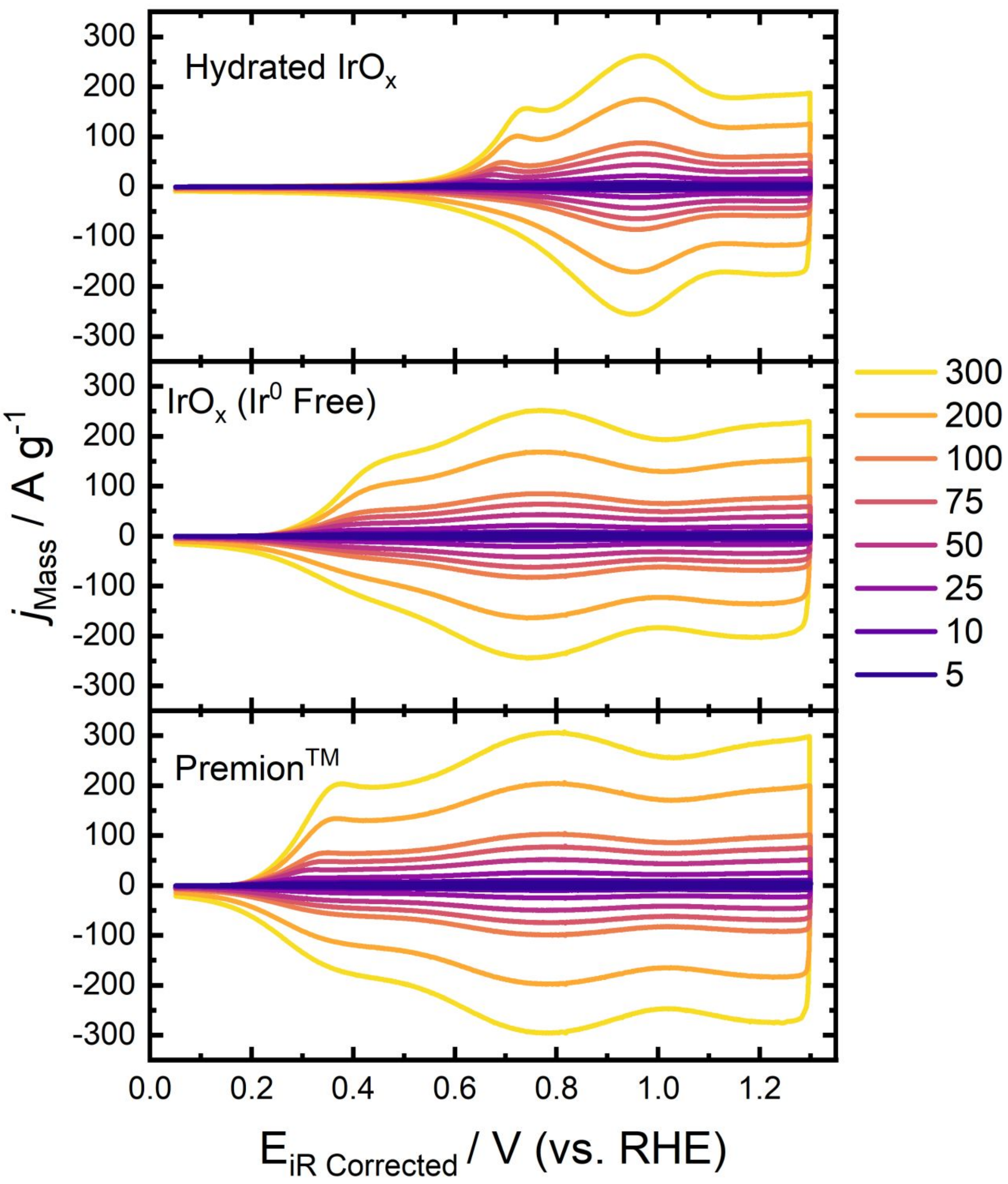

Figure S14 Cyclic voltammetry of the Premion™, IrO_x (Ir^0 Free) and Hydrated IrO_x catalysts at different scan rates. Measured using the floating electrode technique in N_2 purged 1 M H_2SO_4.

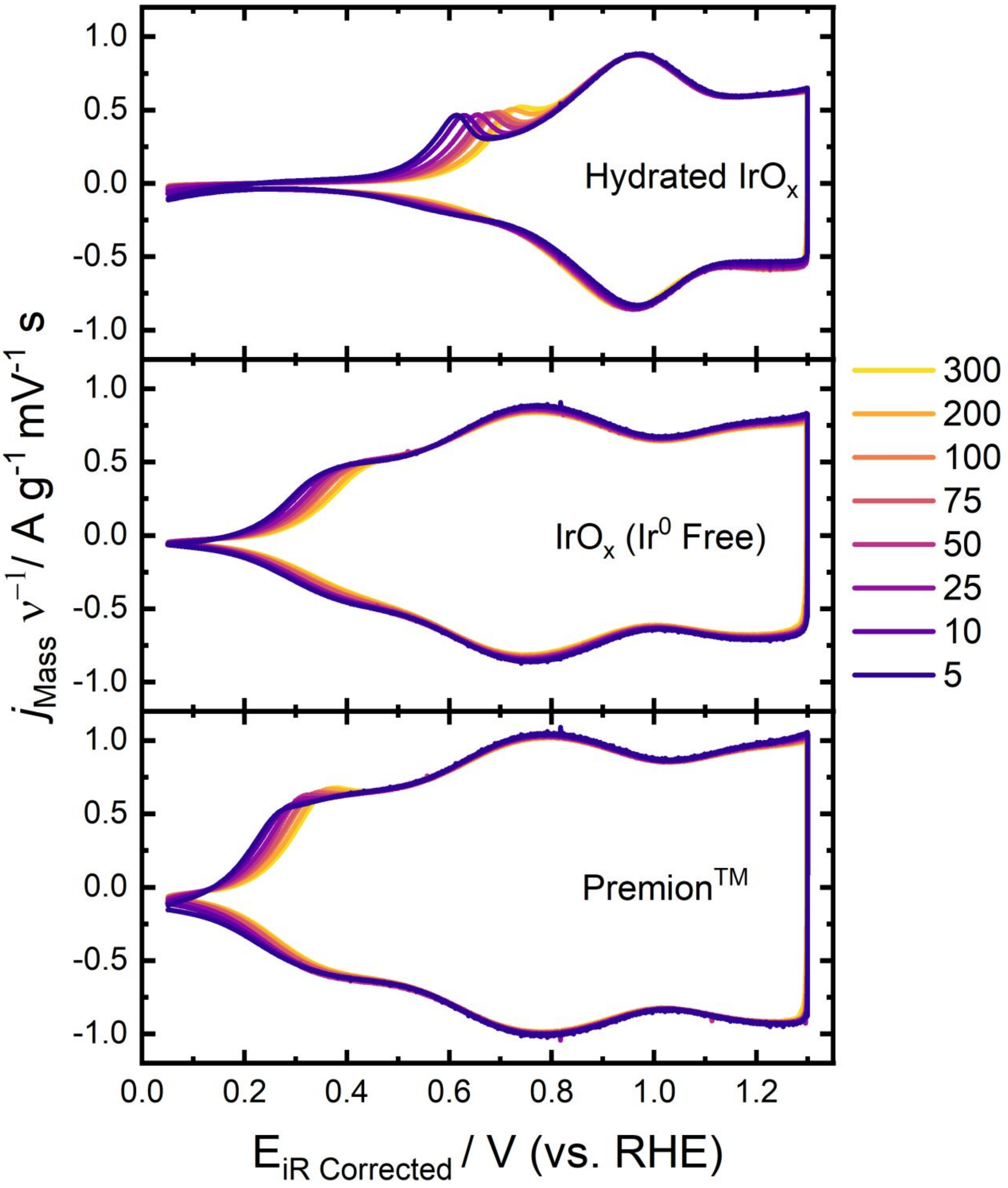

Figure S15 Scan rate normalised and HER Corrected CVs showing the CV shape change with varying scan rate. Measured using the floating electrode technique in N_2 purged 1 M H_2SO_4.

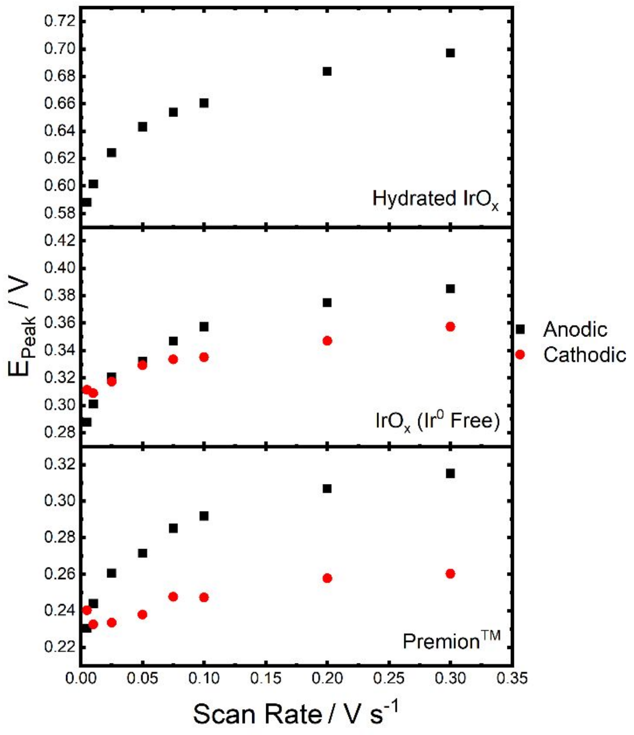

Figure S16 Scan rate dependence of the first anodic and cathodic peak for the Premion™, IrO_x (Ir^0 Free) and Hydrated IrO_x materials measured by FE Technique. The first redox peak position was determined through the maximum in the first derivative. This method gave a more accurate determination of the peak position without contribution from the second redox peak. No cathodic peak for the Hydrated IrO_x is shown because the peak is too small for its position to be accurately determined.

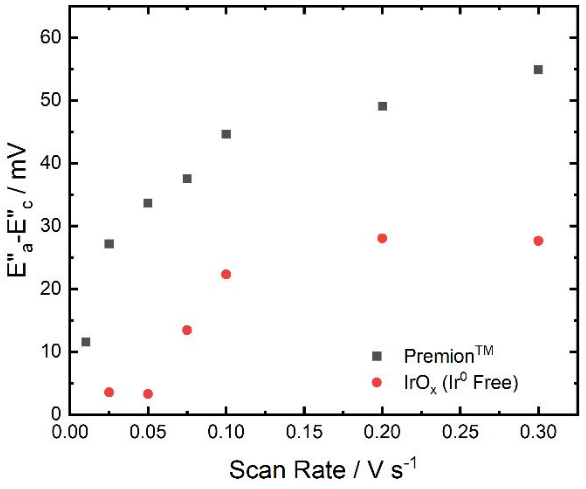

Figure S17 Potential separation of the first redox as a function of the scan rate calculated from Figure S16.

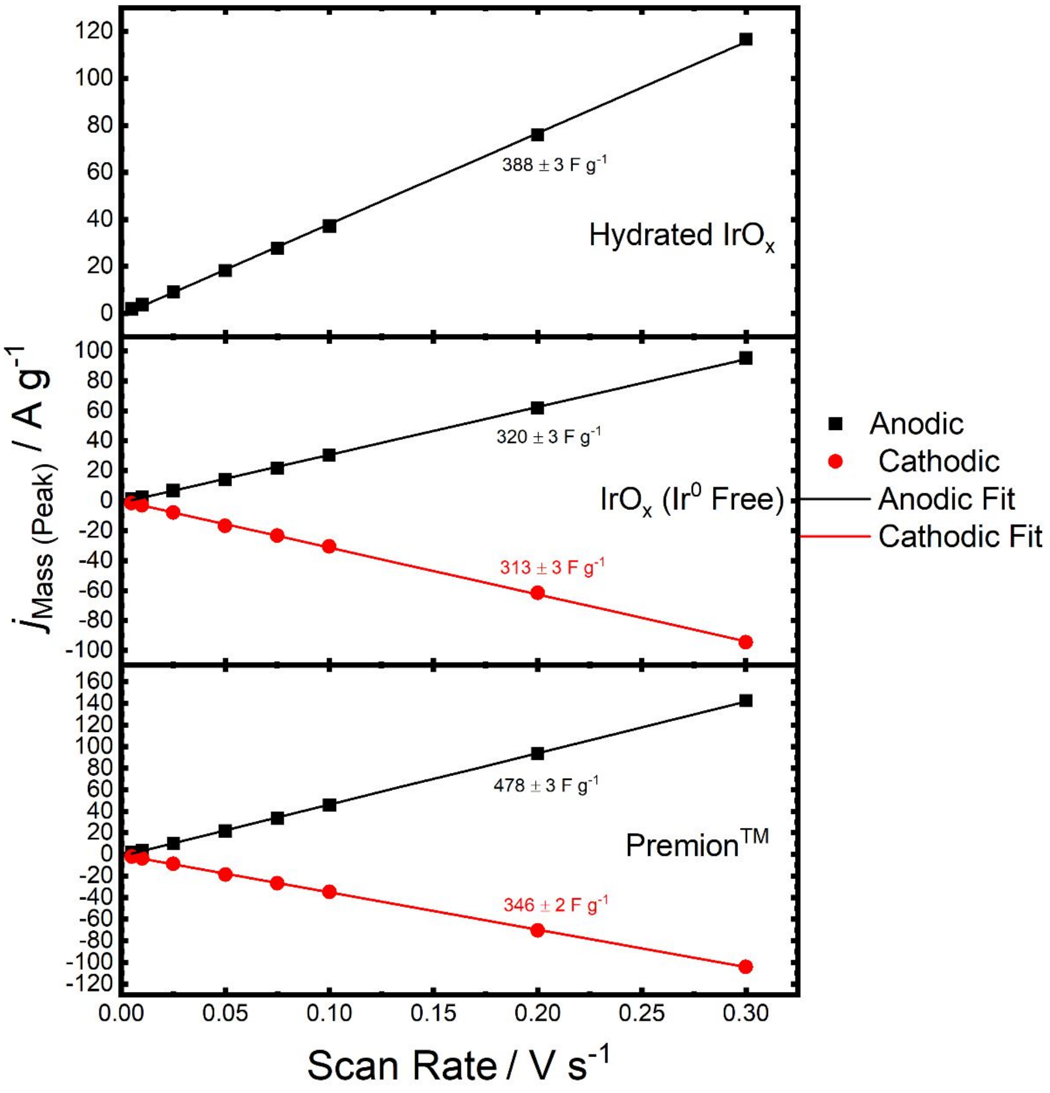

Figure S18 Peak currents for the anodic and cathodic peaks E_a'' as a function of scan rate. Measured using the floating electrode technique in N_2 purged 1 M H_2SO_4.

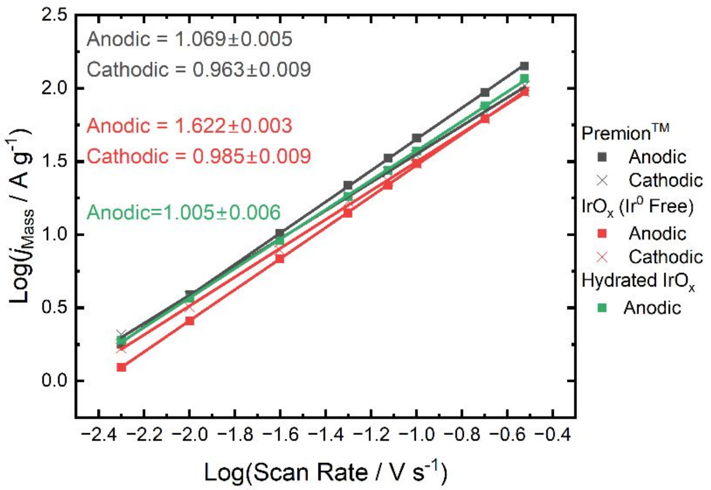

Figure S19 A plot of the *log(Scan Rate)* against the log of the mass activity with the respective slopes shown for the anodic and cathodic peaks. Measured using the floating electrode technique in N_2 purged 1 M H_2SO_4.

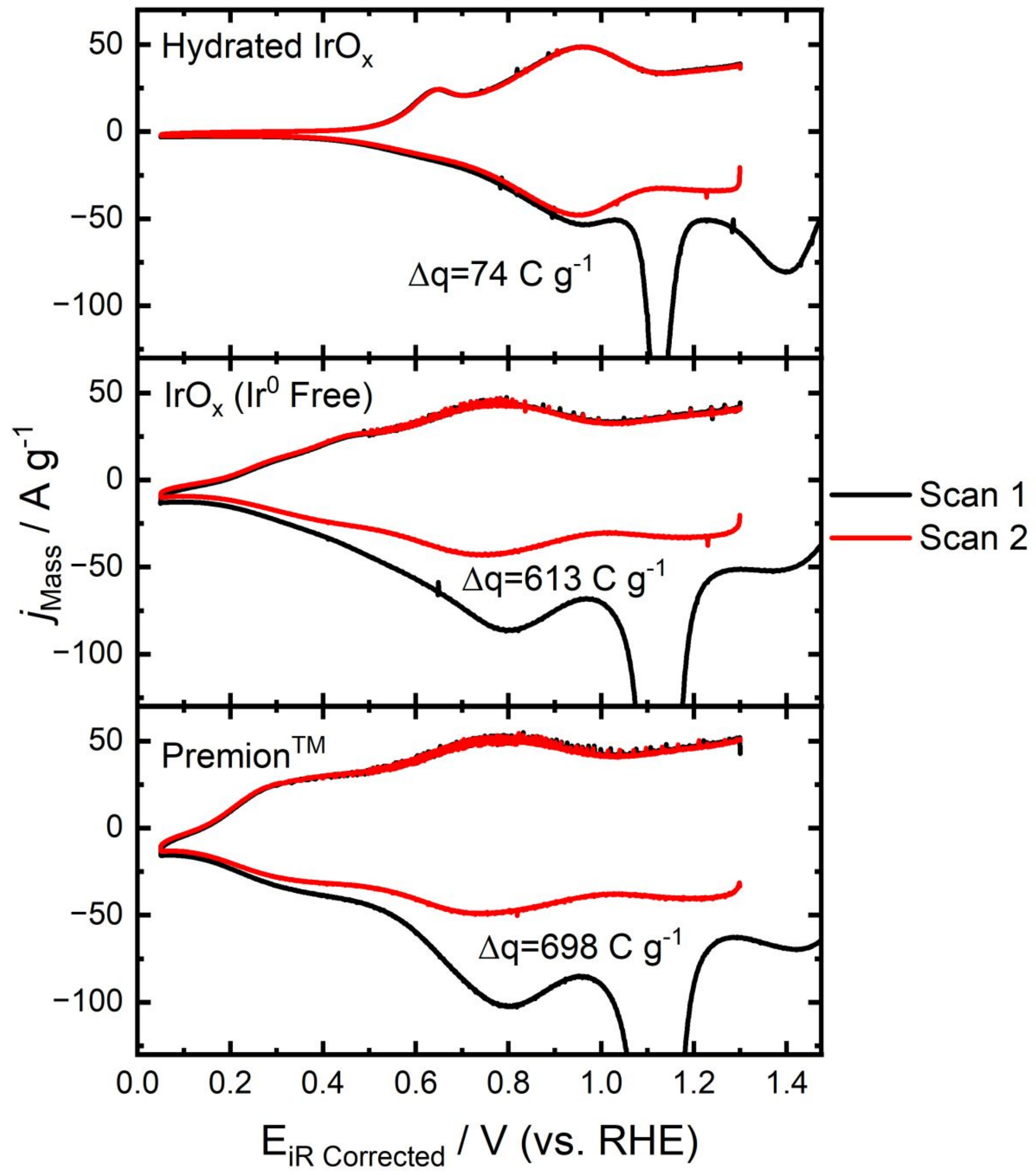

Figure S20 First CV cycles (50 mV s^{-1}) after interrupting the OER cycles measured using the floating electrode technique in N_2 purged 1 M H_2SO_4. The extra reductive charge (Δq) on the first cycle after subtraction of the normalized bare Au CV to remove contributions from the current collector. The CVs shown above are prior to Au subtraction and show the Au reduction peaks at around 1.125 V_{RHE}.

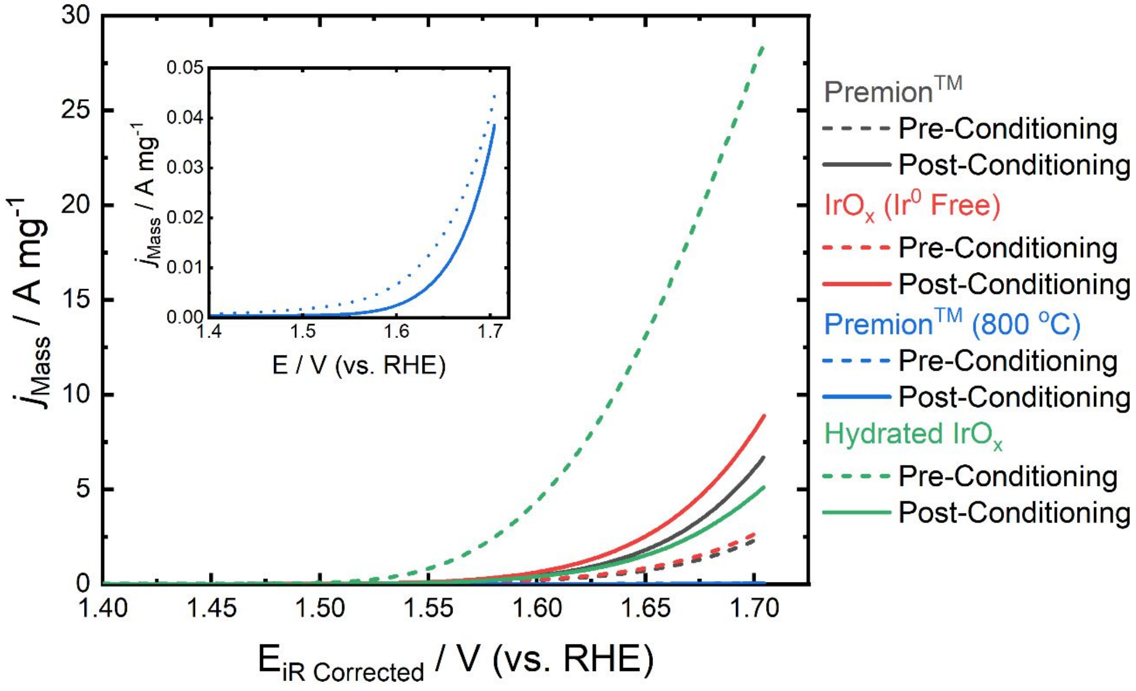

Figure S21 Comparison of the pre- and post- conditioning LSVs of the iridium oxide materials. Insert shows a zoomed in view of the Premion™ (800 °C) conditioning LSVs. Conditioning consists of 35 CVs at 50 mV s^{-1} between 1.3-1.7 V vs. RHE. Measured by the floating electrode technique in N_2 purged 1 M H_2SO_4 at 10 mV s^{-1}.

Table S6 Mass activity of the various iridium oxide materials at 1.6 V_{RHE} before and after conditioning cycles.

	Premion™	IrO_x (Ir^0 Free)	Premion™ (800 °C)	Hydrated IrO_x
j_{Mass} @ 1.6 V_{RHE} Pre-Conditioning / A mg^{-1}	0.21	0.24	0.0067	4.41
j_{Mass} @ 1.6 V_{RHE} Post-Conditioning / A mg^{-1}	0.46	0.64	0.0026	0.41
Change / %	119.04	166.67	-61.19	-90.70

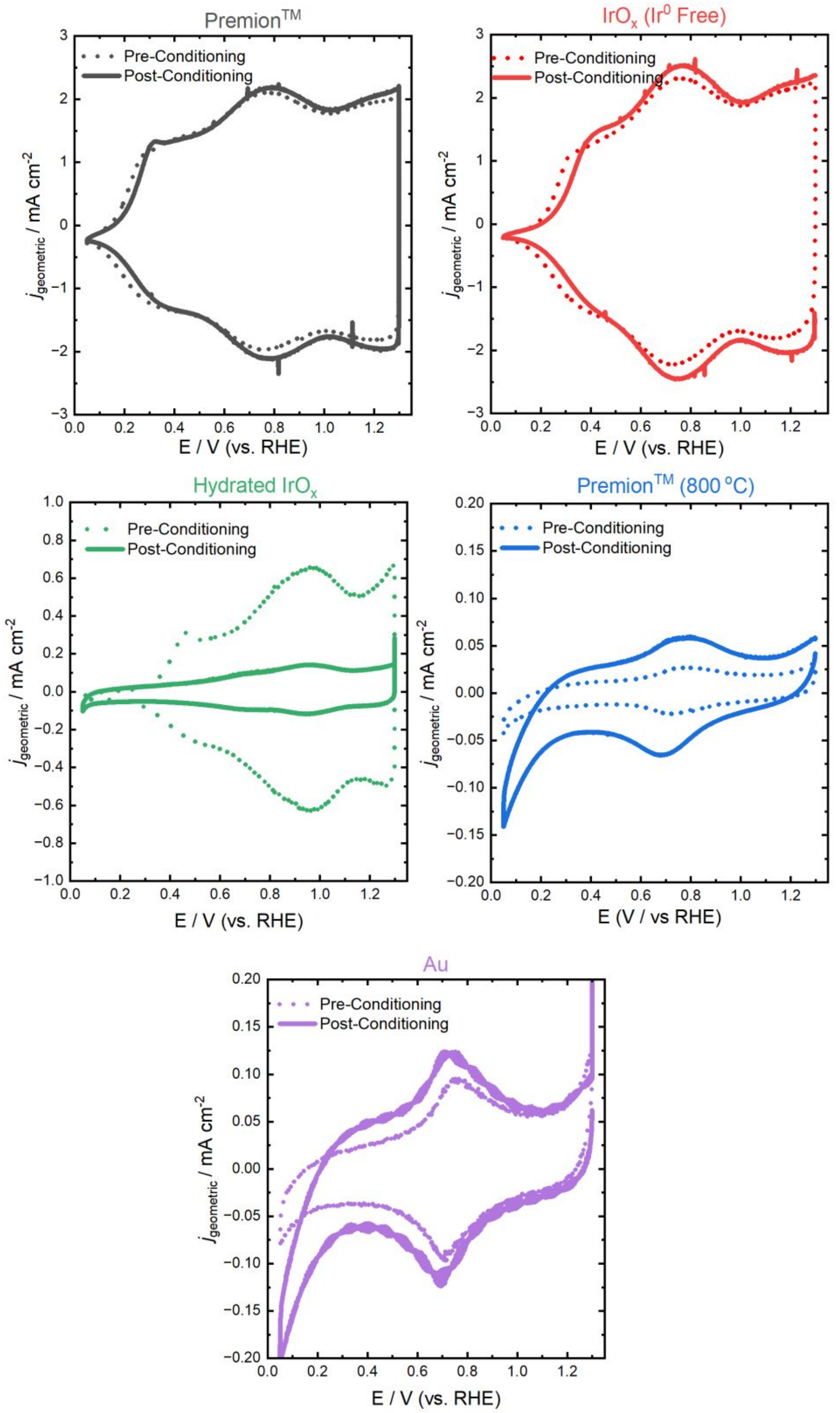

Figure S22 Comparison of the CVs before and after conditioning cycles. Measured by the floating electrode technique in N_2 purged 1 M H_2SO_4 at 50 mV s^{-1}.

Table S7 Mass normalised CV charge of the before and after conditioning cycles (Figure 13). This is calculated for the whole CV potential range between 0.05 – 1.30 V_{RHE}.

	Premion™	IrO_x (Ir^0 Free)	Premion™ (800 °C)	Hydrated IrO_x
Pre-conditioning CV Charge / C g^{-1}	1768	1307	62	1310
Post-conditioning CV Charge/ C g^{-1}	1798	1366	167	300
ΔCharge / C g^{-1}	30	59	105	-1010

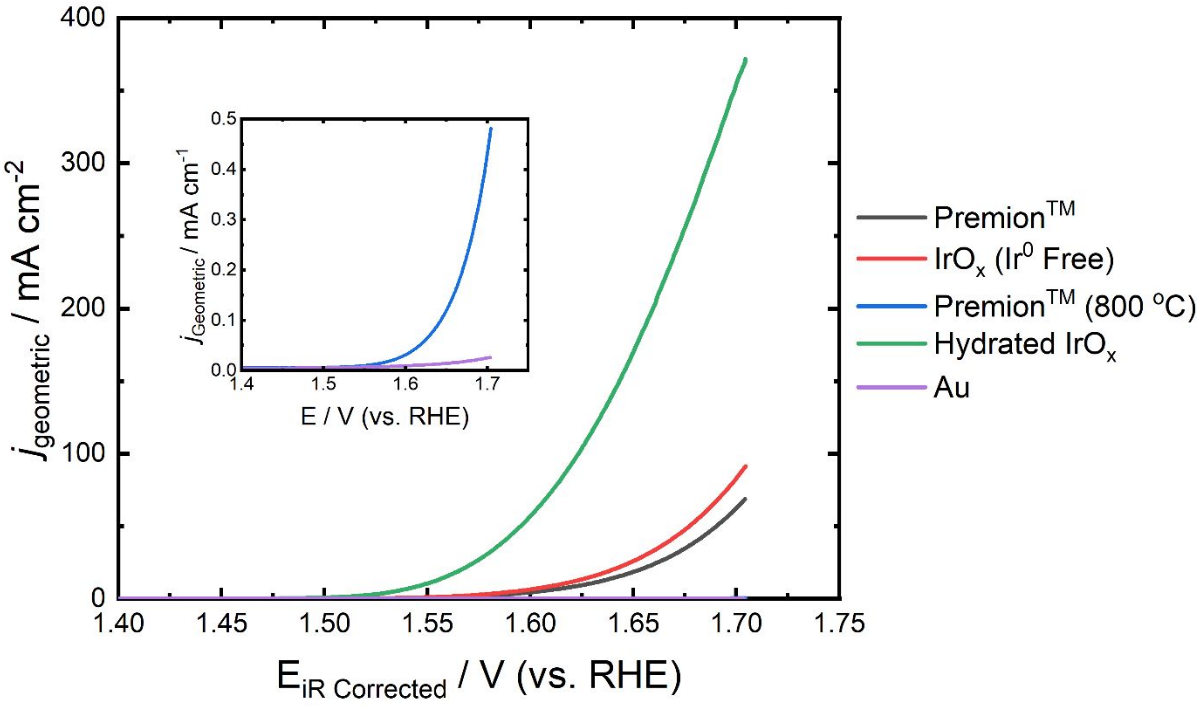

Figure S23 LSV comparison of the bare Au current collector to the geometric activity of the iridium oxide materials. Insert shows a zoomed in view of the Au and Premion™ (800 °C) activities. Measured by the floating electrode technique in N_2 purged 1 M H_2SO_4 at 10 mV s^{-1}.

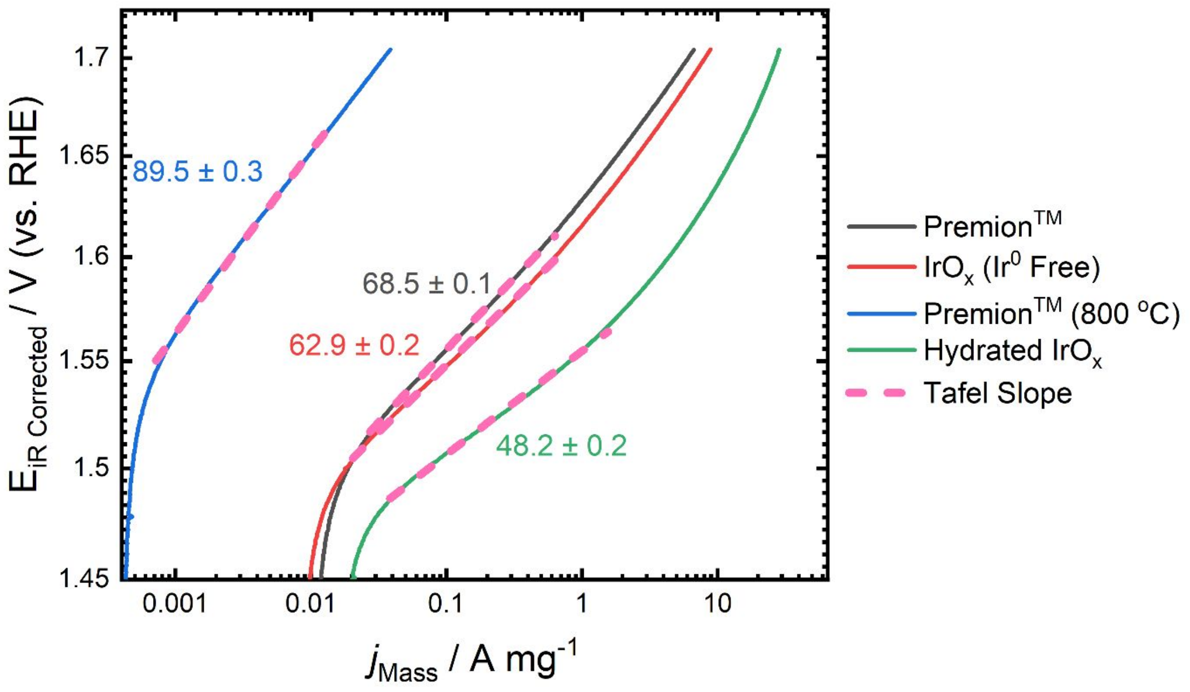

Figure S24 Tafel slopes for the iridium oxide catalysts. Measured using the FE technique in N_2 purged 1 M H_2SO_4 at 10 mV s^{-1}.

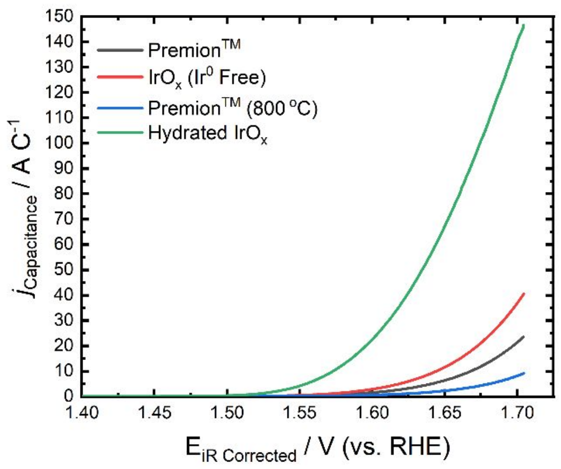

Figure S25 Capacitance normalised LSVs of the iridium oxide catalysts tested using the floating electrode technique. Measured in N_2 purged 1 M H_2SO_4 at 10 mV s^{-1}. Capacitance values taken from the integrated area of 10 mV s^{-1} CVs between 1.15 and 1.3 V_{RHE}.

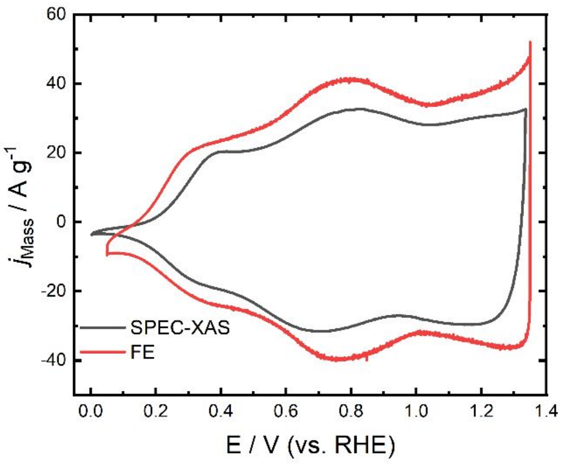

Figure S26 Comparison of the cyclic voltammograms of the Premion ™ on Au/PTFE electrodes in the SPEC-XAS cell compared to floating electrode (FE) measurements of the Premion ™ on Au/PCTE. All measurements were made in N_2 purged 1 M H_2SO_4 with a 50 mV s^{-1} scan rate. The loading of the SPEC-XAS cell electrode was 0.19 mg_{Ir} cm^{-2} while the loading of the FE is 10.3 μg_{Ir} cm^{-2}.

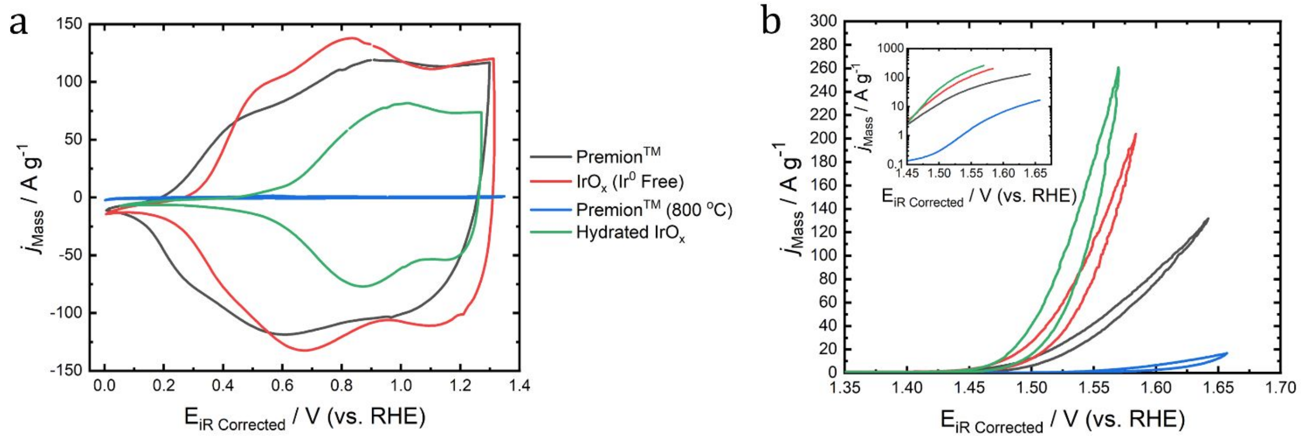

Figure S27 Mass-normalised cyclic voltammetry (a) recorded at 0.20 V s^{-1} and linear sweep voltammetry (b) recoded at 0.001 V s^{-1} with an inset showing the Tafel plot. Measured in the SPEC-XAS cell under N_2 purging (50 ml min^{-1}) and flowing (5 ml min^{-1}) 1 M H_2SO_4 electrolyte.

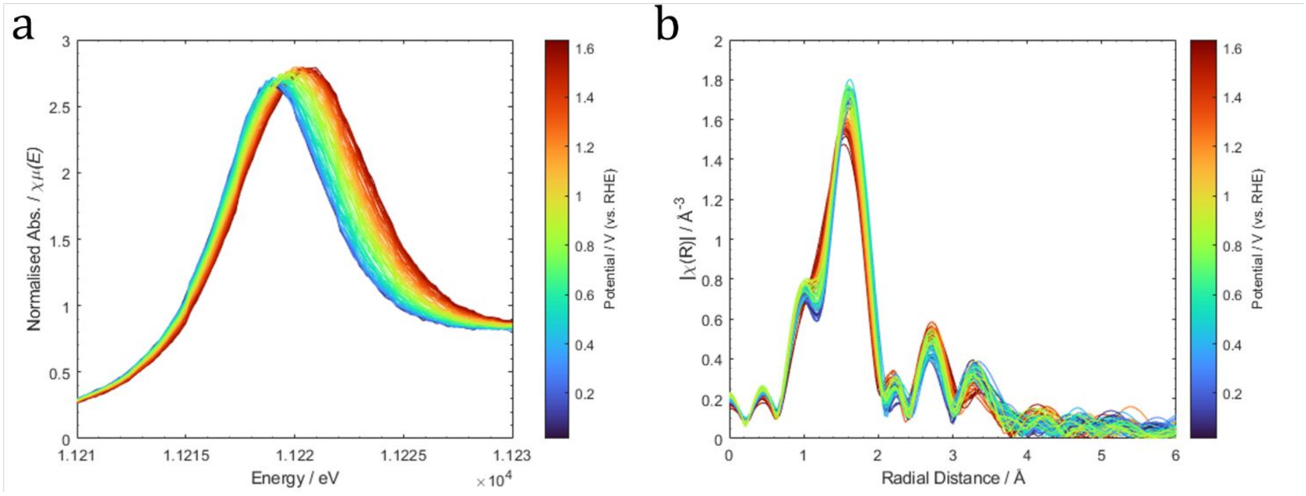

Figure S28 Potential resolved normalized XANES and Fourier transform EXAFS (k^2-weighted and a maximum k of 12 $Å^{-1}$) of the Premion™ during potentiodynamic XAS measurements between 0.0 – 1.70 V_{RHE}. Measurements were made in the SPEC-XAS cell in flowing (5 ml min^{-1}) 1 M H_2SO_4 electrolyte. Under constant N_2 purging.

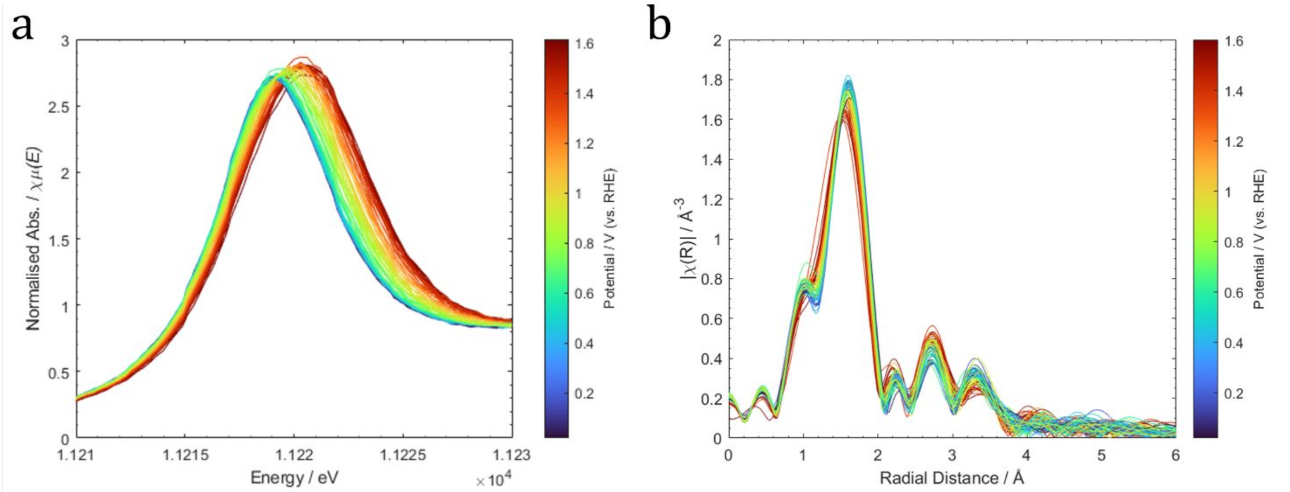

Figure S29 Potential resolved normalized XANES and Fourier transform EXAFS (k^2-weighted and a maximum k of 12 $Å^{-1}$) of the IrO_x (Ir^0 Free) during potentiodynamic XAS measurements between 0.0 – 1.70 V_{RHE}. Measurements were made in the SPEC-XAS cell in flowing (5 ml min^{-1}) 1 M H_2SO_4 electrolyte. Under constant N_2 purging.

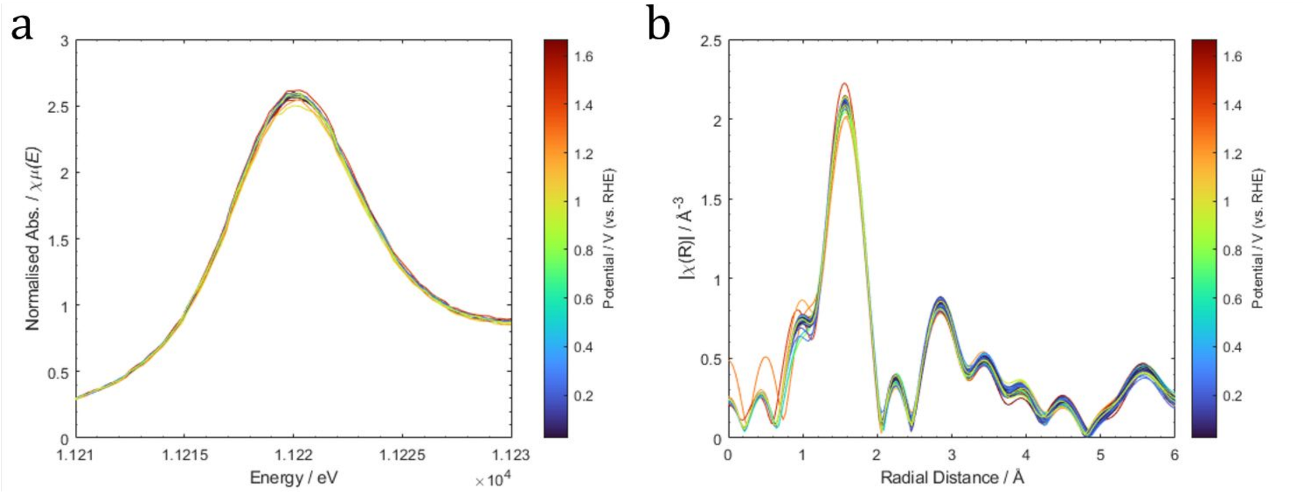

Figure S30 Potential resolved normalized XANES and Fourier transform EXAFS (k^2-weighted and a maximum k of 12 $Å^{-1}$) of the Premion™ (800 °C) during potentiodynamic XAS measurements between 0.0 – 1.70 V_{RHE}. Measurements were made in the SPEC-XAS cell in flowing (5 ml min^{-1}) 1 M H_2SO_4 electrolyte. Under constant N_2 purging.

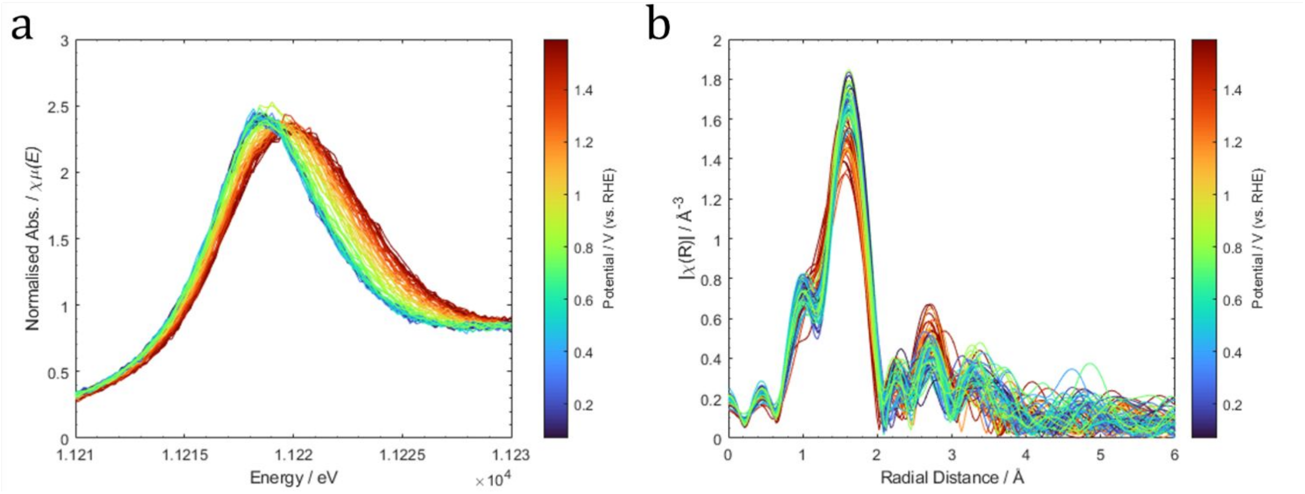

Figure S31 Potential resolved normalized XANES and Fourier transform EXAFS (k^2-weighted and a maximum k of 12 $Å^{-1}$) of the Hydrated IrO_x during potentiodynamic XAS measurements between 0.0 – 1.70 V_{RHE}. Measurements were made in the SPEC-XAS cell in flowing (5 ml min^{-1}) 1 M H_2SO_4 electrolyte. Under constant N_2 purging.

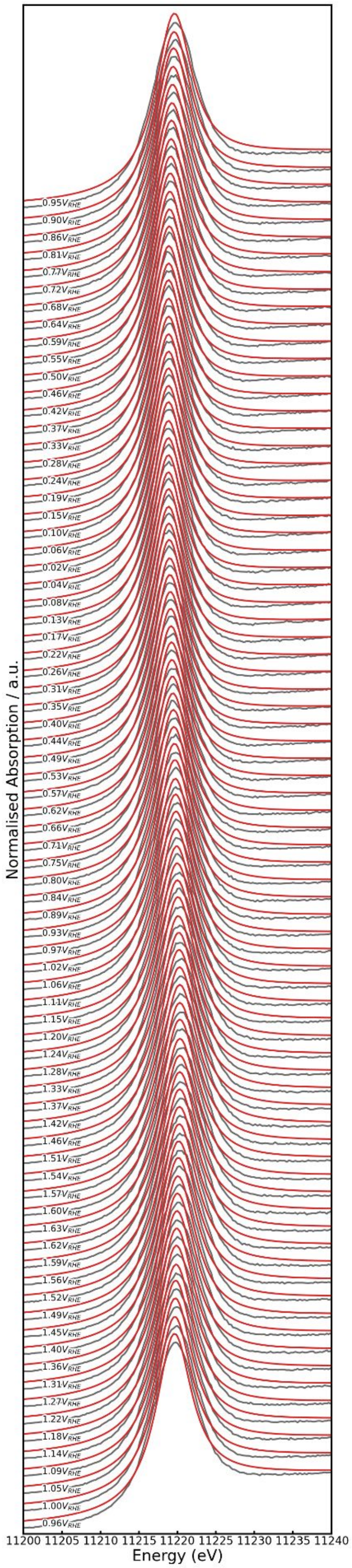

Figure S32 Normalized Ir L_{III} edge for the Premion™ electrodes as a function of the potential. The XANES were fit using an arctangent background function and the white line fit using a Lorentzian function. Above shows the overall fitted XANES for each potential measured. This fitting was used to extract the edge positions, areas, FWHMs.

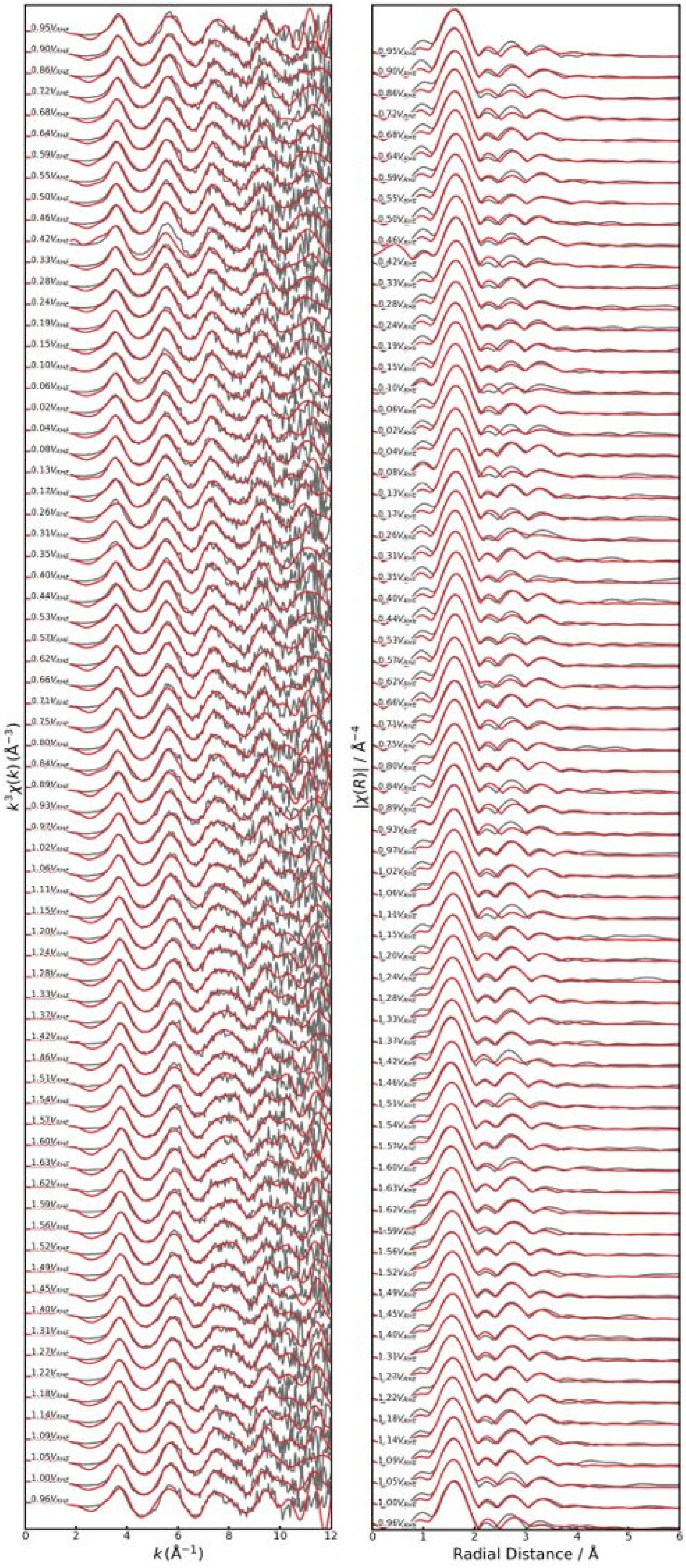

Figure S33 k^3-weighted $\chi(k)$ and Fourier transform EXAFS fits of the Premion™ *in situ* during a CV between 0-1.70 V_{RHE} fit using the model in Table S1 and the fitted parameters shown inTable S8 Fitting was performed in r space between 1- 4 Å, a fitting *k*-weight of 3, a hanning window with a tampering of 1 and a maximum *k* of 12 Å^{-1}.

Table S8 Fitted EXAFS parameters for the Premion™ *in situ* data.*

Potential / V_{RHE}	NO1	Irabs-O1 / Å	Irabs-Ir1 / Å	Irabs-Ir2 / Å	SSO1 / Å2	SSIr1 / Å2	SSIr2 / Å2	R Factor
0.96	6(1)	1.99(2)	3.43(4)	3.61(5)	0.005(2)	-0.004(5)	0.005(6)	0.05
1.00	6.9(7)	1.991(7)	3.13(2)	3.84(6)	0.005(1)	0.003(2)	0.021(9)	0.01
1.05	7(1)	1.99(1)	2.73(4)	3.82(6)	0.005(2)	0.008(5)	0.015(7)	0.04
1.09	7.0(9)	1.989(9)	3.09(2)	3.7(1)	0.006(1)	0.002(2)	0.03(2)	0.02
1.14	7.0(8)	1.982(8)	3.12(1)	3.7(1)	0.006(1)	0.001(1)	0.03(1)	0.01
1.18	6.6(8)	1.979(8)	3.08(2)	3.77(9)	0.006(1)	0.003(2)	0.02(1)	0.02
1.22	7.1(9)	1.977(9)	3.10(2)	3.72(7)	0.007(2)	0.002(2)	0.02(1)	0.02
1.27	6.7(7)	1.964(7)	3.08(1)	3.71(5)	0.006(1)	0.002(1)	0.021(7)	0.01
1.31	7.5(7)	1.963(7)	3.11(1)	3.73(5)	0.007(1)	0.001(1)	0.019(6)	0.01
1.36	6(2)	1.96(2)	4.7(2)	3.7(4)	0.006(3)	0.00(1)	0.03(6)	0.06
1.40	7.0(8)	1.968(8)	3.09(2)	3.65(4)	0.007(1)	0.003(2)	0.015(5)	0.02
1.45	7(1)	1.96(1)	3.09(1)	3.7(1)	0.008(2)	0.000(1)	0.03(1)	0.02
1.49	6.9(8)	1.960(8)	3.10(2)	3.68(4)	0.006(1)	0.001(2)	0.016(5)	0.02
1.52	8(1)	1.96(1)	3.12(2)	3.7(1)	0.008(2)	0.002(2)	0.02(2)	0.02
1.56	7.0(8)	1.956(9)	3.11(2)	3.71(4)	0.007(1)	0.002(2)	0.015(5)	0.02
1.59	8(1)	1.94(1)	3.11(2)	3.6(1)	0.009(2)	0.002(2)	0.03(2)	0.03
1.62	6.8(9)	1.950(9)	3.12(2)	3.7(1)	0.006(1)	0.002(2)	0.03(2)	0.02
1.63	6.9(8)	1.943(8)	3.10(2)	3.62(6)	0.006(1)	0.001(2)	0.019(8)	0.01
1.60	7(1)	1.95(1)	3.75(5)	3.54(4)	0.006(2)	-0.002(4)	0.008(4)	0.03
1.57	6.5(7)	1.945(8)	3.09(2)	3.66(6)	0.006(1)	0.001(2)	0.020(8)	0.01
1.54	6.5(7)	1.951(8)	3.11(1)	3.71(9)	0.006(1)	0.001(1)	0.02(1)	0.01
1.51	7(1)	1.94(1)	3.44(4)	3.62(7)	0.006(2)	-0.001(5)	0.011(9)	0.04
1.46	7.0(6)	1.955(6)	3.08(1)	3.67(3)	0.007(1)	0.002(1)	0.015(4)	0.009
1.42	6(1)	1.96(1)	2.32(6)	3.7(3)	0.004(2)	0.013(7)	0.04(5)	0.04

1.37	6.9(8)	1.960(9)	3.10(2)	3.66(4)	0.007(1)	0.004(2)	0.017(6)	0.02
1.33	7.2(9)	1.961(9)	3.10(2)	3.69(8)	0.007(1)	0.002(2)	0.02(1)	0.02
1.28	7.0(9)	1.970(9)	3.11(2)	3.71(7)	0.007(1)	0.001(2)	0.02(1)	0.02
1.24	6.8(8)	1.978(8)	3.12(2)	3.67(6)	0.006(1)	0.001(2)	0.020(9)	0.02
1.20	6.7(8)	1.978(8)	3.11(2)	3.7(1)	0.006(1)	0.002(2)	0.03(1)	0.02
1.15	7(1)	1.98(1)	3.09(2)	3.68(8)	0.007(2)	0.003(3)	0.02(1)	0.02
1.11	4(2)	2.01(2)	1.59(5)	4.0(2)	0.001(4)	0.015(8)	0.03(3)	0.05
1.06	7.4(8)	1.986(8)	3.11(2)	3.68(6)	0.007(1)	0.001(2)	0.019(8)	0.01
1.02	6.9(8)	1.999(9)	3.12(2)	3.8(1)	0.006(1)	0.004(2)	0.03(2)	0.02
0.97	7(1)	2.00(1)	3.11(2)	3.68(4)	0.006(2)	0.001(2)	0.012(4)	0.02
0.93	5(1)	2.01(2)	1.58(7)	3.8(3)	0.002(3)	0.02(1)	0.03(4)	0.04
0.89	6(1)	2.00(1)	3.48(4)	3.68(7)	0.004(2)	-0.002(5)	0.011(9)	0.04
0.84	6(1)	2.01(1)	4.11(9)	3.9(1)	0.004(2)	-0.002(9)	0.01(1)	0.05
0.80	7.3(9)	2.009(9)	3.13(2)	3.8(1)	0.006(1)	0.001(2)	0.02(2)	0.02
0.75	6.3(9)	2.013(9)	3.12(4)	3.8(3)	0.004(1)	0.006(4)	0.04(5)	0.02
0.71	7(1)	2.01(1)	3.49(5)	3.69(8)	0.005(2)	-0.001(6)	0.01(1)	0.04
0.66	6.5(7)	2.015(7)	3.11(2)	3.72(7)	0.004(1)	0.002(2)	0.019(9)	0.01
0.62	7(1)	2.02(1)	2.71(3)	3.87(6)	0.005(2)	0.008(4)	0.016(8)	0.03
0.57	6.2(7)	2.023(7)	3.14(2)	3.9(2)	0.003(1)	0.004(3)	0.03(2)	0.02
0.53	6(1)	2.03(1)	2.75(4)	3.9(1)	0.003(2)	0.009(5)	0.02(1)	0.03
0.49	6(1)	2.03(1)	5.05(8)	3.89(8)	0.002(2)	-0.010(7)	0.02(1)	0.03
0.44	6(1)	2.04(1)	2.77(4)	3.9(1)	0.002(2)	0.007(4)	0.02(1)	0.04
0.4	6.5(9)	2.038(9)	3.14(2)	3.9(7)	0.003(2)	0.003(3)	0.0(1)	0.02
0.35	5.9(9)	2.03(1)	2.73(5)	3.62(7)	0.002(2)	0.010(6)	0.02(1)	0.03
0.31	6.1(8)	2.031(8)	3.12(2)	3.9(8)	0.003(1)	0.003(2)	0.1(2)	0.02
0.26	6(1)	2.04(1)	2.71(4)	3.58(4)	0.003(2)	0.007(4)	0.011(4)	0.03

0.22	6(1)	2.04(1)	5.38(7)	3.92(8)	0.003(2)	-0.013(7)	0.02(1)	0.03
0.17	7(1)	2.04(1)	3.53(7)	3.7(1)	0.004(2)	0.003(9)	0.02(2)	0.04
0.13	6.4(7)	2.041(7)	3.12(2)	3.33(7)	0.004(1)	0.001(2)	0.03(1)	0.01
0.08	5(1)	2.05(2)	1.57(9)	3.9(2)	0.002(2)	0.02(1)	0.03(2)	0.04
0.04	6.7(8)	2.035(8)	3.12(2)	3.8(2)	0.005(1)	0.001(2)	0.03(3)	0.02
0.02	6(1)	2.04(1)	3.9(3)	3.56(8)	0.003(2)	0.01(4)	0.02(1)	0.04
0.06	6.6(9)	2.046(9)	3.14(3)	3.9(2)	0.004(1)	0.005(3)	0.03(3)	0.02
0.10	6(2)	2.04(2)	0.9(2)	3.57(6)	0.003(3)	0.05(5)	0.015(7)	0.04
0.15	6.0(8)	2.040(9)	2.74(3)	3.90(5)	0.003(1)	0.009(4)	0.015(6)	0.02
0.19	7(1)	2.04(1)	3.12(3)	3.9(5)	0.005(2)	0.006(4)	0.05(9)	0.02
0.24	6.1(9)	2.04(1)	2.73(3)	3.84(9)	0.003(2)	0.008(4)	0.02(1)	0.03
0.28	6(1)	2.04(1)	2.75(5)	3.93(9)	0.004(2)	0.011(6)	0.02(1)	0.03
0.33	6.7(9)	2.05(1)	3.13(3)	3.9(2)	0.005(2)	0.005(3)	0.03(2)	0.02
0.37	6(1)	2.04(1)	4.75(5)	3.93(7)	0.002(2)	-0.010(5)	0.015(9)	0.03
0.42	4(1)	2.04(1)	0.82(7)	4.0(3)	0.000(3)	0.03(1)	0.03(4)	0.03
0.46	6(1)	2.04(1)	2.76(3)	3.91(6)	0.003(2)	0.007(4)	0.015(7)	0.03
0.50	6(1)	2.03(1)	3.12(4)	3.9(3)	0.003(2)	0.007(5)	0.04(5)	0.03
0.55	6.9(9)	2.033(9)	3.15(5)	3.98(9)	0.005(1)	0.008(5)	0.02(1)	0.02
0.59	6(1)	2.03(1)	2.75(5)	3.86(6)	0.004(2)	0.010(7)	0.014(7)	0.04
0.64	6.4(8)	2.025(8)	3.14(3)	3.8(4)	0.003(1)	0.006(3)	0.04(6)	0.02
0.68	7.0(9)	2.017(9)	3.13(2)	3.8(2)	0.005(1)	0.003(3)	0.03(4)	0.02
0.72	6(1)	2.02(1)	2.4(1)	3.88(7)	0.003(2)	0.02(1)	0.016(9)	0.04
0.77		2.01(1)						0.05
0.81		2.01(1)						0.05
0.86	7(1)	2.01(2)	2.75(4)	3.88(7)	0.005(2)	0.007(5)	0.014(8)	0.05
0.90	6.2(9)	2.006(9)	3.14(3)	3.9(1)	0.004(1)	0.004(3)	0.03(2)	0.02

0.95	7(1)	2.00(1)	3.46(4)	3.66(6)	0.005(2)	-0.004(4)	0.009(8)	0.05

*The amplitude reduction factor was fixed at 0.78 for all fitting, calculated from fitting a crystalline iridium oxide. Fitting was performed in r space between 1- 4 Å, a fitting *k*-weight of 3, a hanning window with a tampering of 1 and a maximum *k* of 12 $Å^{-1}$.

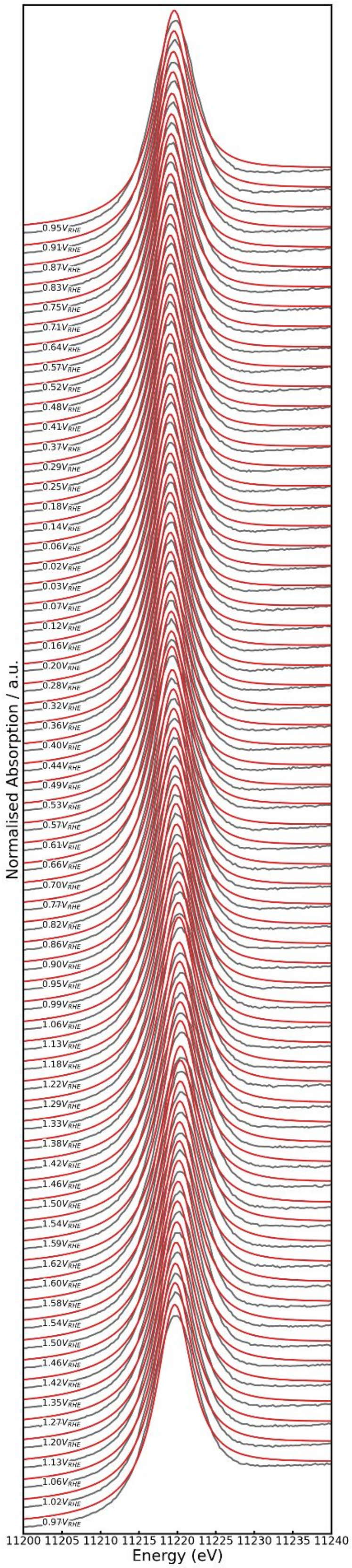

Figure S34 Normalized Ir L_{III} edge for the IrO_x (Ir^0 Free) electrodes as a function of the potential. The XANES were fit using an arctangent background function and the white line fit using a Lorentzian function. Above shows the overall fitted XANES for each potential measured. This fitting was used to extract the edge positions, areas, FWHMs.

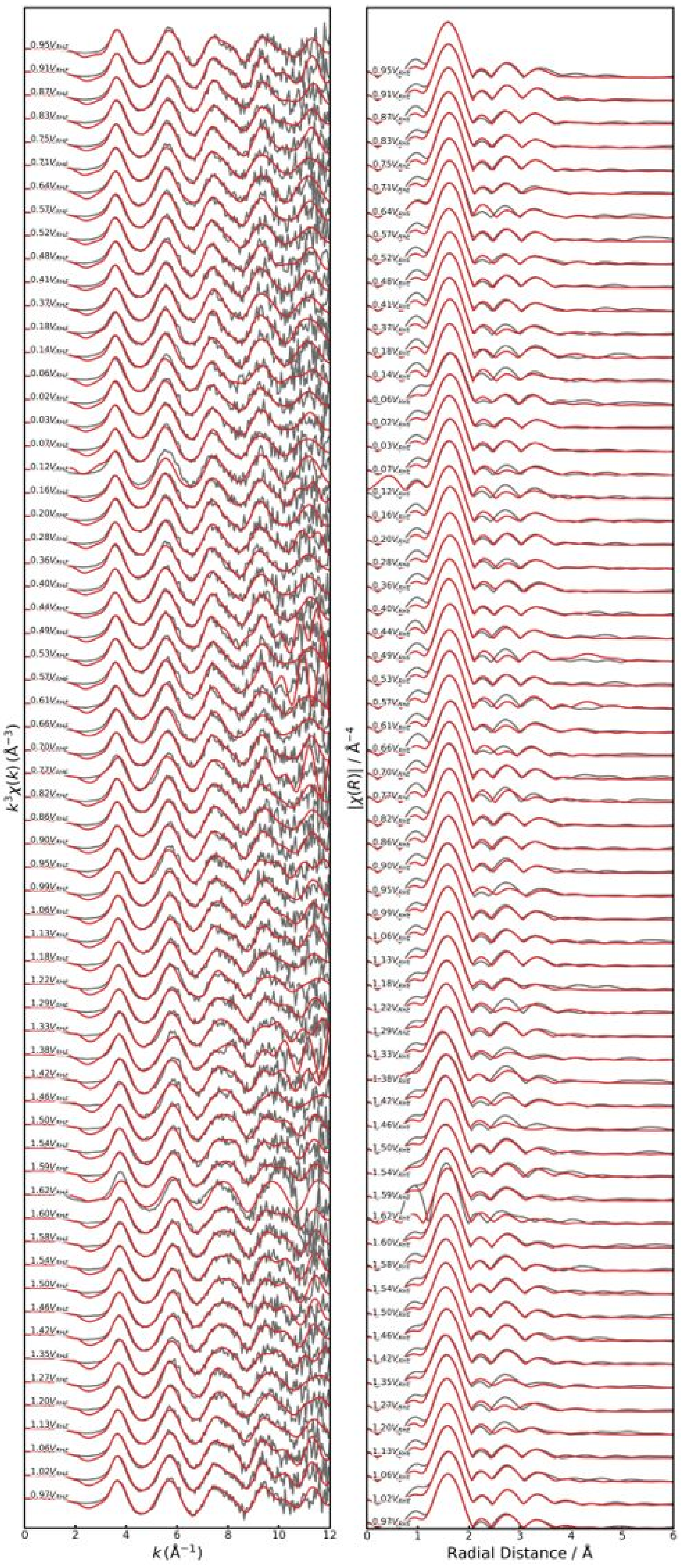

Figure S35 k^3-weighted *χ(k)* and Fourier transform EXAFS fits of the IrO_x (Ir^0 Free) *in situ* during a CV between 0-1.70 V_{RHE} fit using the model in Table S1 and the fitted parameters shown in Table S9 Fitting was performed in r space between 1- 4 Å, a fitting *k*-weight of 3, a hanning window with a tampering of 1 and a maximum *k* of 12 Å^{-1}.

Table S9 Fitted EXAFS parameters for the IrO_x (Ir^0 Free) *in situ* data.*

Potential / V_{RHE}	N_{O1}	Ir_{abs}-O_1 / Å	Ir_{abs}-Ir_1 / Å	Ir_{abs}-Ir_2 / Å	SS_{O1} / $Å^2$	SS_{Ir1} / $Å^2$	SS_{Ir2} / $Å^2$	R Factor
0.97	6.8(8)	1.998(8)	3.13(2)	3.76(9)	0.005(1)	0.003(2)	0.02(1)	0.01
1.02	7(1)	2.00(1)	3.14(2)	3.78(6)	0.006(2)	0.001(2)	0.017(8)	0.03
1.06	6.6(8)	1.987(9)	3.14(2)	3.8(2)	0.005(1)	0.001(2)	0.03(2)	0.02
1.13	6.8(8)	1.989(8)	3.13(3)	3.80(8)	0.005(1)	0.005(3)	0.02(1)	0.02
1.20	6.5(9)	1.98(1)	4.4(2)	3.8(2)	0.005(2)	0.01(2)	0.03(4)	0.03
1.27	7(1)	1.97(1)	4.4(3)	3.8(1)	0.005(2)	0.01(4)	0.02(1)	0.04
1.35	7(1)	1.96(1)	3.78(8)	3.57(6)	0.006(2)	0.001(7)	0.011(7)	0.03
1.42	6.9(8)	1.968(9)	3.13(2)	3.72(8)	0.005(1)	0.003(2)	0.02(1)	0.02
1.46	7.2(7)	1.957(7)	3.11(1)	3.72(5)	0.006(1)	0.001(1)	0.020(7)	0.01
1.50	6.4(6)	1.955(7)	3.11(1)	3.71(7)	0.005(1)	0.002(1)	0.02(1)	0.01
1.54	6.7(8)	1.953(8)	3.11(2)	3.7(1)	0.005(1)	0.001(2)	0.03(1)	0.02
1.58	6.2(7)	1.956(8)	3.11(2)	3.7(1)	0.004(1)	0.003(2)	0.03(2)	0.02
1.60	6.6(6)	1.958(7)	3.13(2)	3.70(5)	0.006(1)	0.004(2)	0.021(7)	0.01
1.62	4(1)	1.96(2)	3.2(1)	4.0(6)	0.000(3)	0.01(1)	0.0(1)	0.13
1.59	7.2(8)	1.954(9)	3.12(2)	3.7(1)	0.007(1)	0.004(3)	0.02(1)	0.02
1.54	7.6(1)	1.96(1)	3.12(3)	3.81(9)	0.007(2)	0.003(3)	0.02(1)	0.03
1.50	6.8(8)	1.957(9)	3.11(2)	3.7(1)	0.006(1)	0.003(2)	0.03(2)	0.02
1.46	6(2)	1.95(2)	2.3(9)	3.53(7)	0.006(3)	0.0(2)	0.016(8)	0.04
1.42	7(1)	1.96(1)	3.47(4)	3.68(9)	0.006(2)	0.000(5)	0.02(1)	0.03
1.38	8(1)	1.92(1)	3.69(5)	3.50(3)	0.008(2)	-0.003(4)	0.005(3)	0.03
1.33	7(1)	1.97(1)	3.82(7)	3.60(7)	0.006(2)	0.001(7)	0.012(8)	0.03
1.29	7.1(8)	1.967(8)	3.11(2)	3.7(1)	0.006(1)	0.003(2)	0.03(2)	0.02
1.22	6(2)	1.98(2)	2.4(3)	3.86(7)	0.006(3)	0.03(5)	0.016(8)	0.05
1.18	7(1)	1.99(1)	3.14(4)	3.8(2)	0.006(2)	0.007(5)	0.03(3)	0.03

1.13	7(1)	1.99(1)	3.12(2)	3.8(1)	0.006(2)	0.002(2)	0.03(2)	0.02
1.06	6.8(8)	1.993(8)	3.13(3)	3.73(6)	0.005(1)	0.004(3)	0.018(9)	0.02
0.99	6.9(8)	1.992(8)	3.13(2)	3.8(1)	0.005(1)	0.004(3)	0.02(1)	0.02
0.95	7(1)	2.00(1)	3.53(5)	3.8(2)	0.006(2)	0.002(6)	0.02(3)	0.04
0.90	6.7(9)	2.00(1)	3.15(3)	3.8(4)	0.004(2)	0.004(3)	0.04(6)	0.02
0.86	6.6(8)	2.005(8)	3.14(3)	3.8(2)	0.004(1)	0.007(4)	0.03(3)	0.02
0.82	6.4(9)	2.014(9)	3.18(3)	3.9(1)	0.004(1)	0.004(3)	0.03(2)	0.02
0.77	6(1)	2.01(1)	3.46(4)	3.65(5)	0.003(2)	-0.004(5)	0.005(6)	0.04
0.70	6.3(8)	2.018(8)	3.14(2)	3.8(2)	0.003(1)	0.003(3)	0.03(3)	0.02
0.66	7(1)	2.04(2)	3.43(9)	3.9(6)	0.004(2)	0.01(1)	0.0(1)	0.06
0.61	6(1)	2.03(1)	2.73(7)	3.90(7)	0.004(2)	0.013(9)	0.016(8)	0.03
0.57	7(1)	2.02(1)	4.09(5)	3.91(6)	0.003(2)	-0.007(5)	0.006(7)	0.04
0.53	6.9(8)	2.026(8)	3.14(3)	3.9(3)	0.005(1)	0.007(4)	0.04(5)	0.02
0.49	6(1)	2.03(1)	4.44(6)	3.91(8)	0.003(2)	-0.005(7)	0.02(1)	0.04
0.44	6.2(6)	2.022(7)	3.14(3)	3.9(2)	0.003(1)	0.007(3)	0.03(3)	0.01
0.40	6.7(8)	2.031(8)	3.16(5)	4.0(1)	0.004(1)	0.009(6)	0.02(2)	0.02
0.36	7(1)	2.040(9)	2.56(5)	3.18(3)	0.005(2)	0.014(7)	0.017(4)	0.02
0.32	6(2)	2.03(2)	2.5(9)	4.82(9)	0.003(2)	0.0(3)	-0.001(9)	0.04
0.28	6.5(9)	2.04(1)	4.1(1)	3.93(8)	0.004(2)	0.00(1)	0.01(1)	0.03
0.20	6.6(8)	2.036(8)	3.15(3)	3.85(9)	0.003(1)	0.005(3)	0.02(1)	0.02
0.16	6(1)	2.03(1)	4.5(1)	3.91(8)	0.004(2)	0.00(2)	0.02(1)	0.03
0.12	4(1)	2.03(1)	0.81(7)	3.9(2)	0.000(2)	0.03(1)	0.03(3)	0.03
0.07	7(1)	2.04(1)	2.6(2)	3.91(7)	0.005(2)	0.02(3)	0.018(9)	0.03
0.03	6.9(7)	2.028(7)	3.15(3)	3.9(2)	0.004(1)	0.006(3)	0.03(3)	0.01
0.02	7(1)	2.04(1)	3.16(4)	3.9(1)	0.005(2)	0.008(5)	0.02(2)	0.02
0.06	8(2)	2.04(1)	3.11(4)	3.9(3)	0.007(2)	0.006(5)	0.03(5)	0.05

0.14	6(1)	2.03(1)	2.4(2)	4.0(2)	0.002(2)	0.02(2)	0.03(3)	0.04
0.18	6(1)	2.03(1)	2.72(9)	3.88(5)	0.002(2)	0.02(1)	0.014(6)	0.03
0.25	5.1(8)	2.03(1)	1.57(5)	4.7(2)	0.001(2)	0.020(6)	0.02(3)	0.02
0.29	7(1)	2.03(1)	5.36(7)	3.9(1)	0.003(2)	-0.013(7)	0.02(1)	0.04
0.37	6.5(9)	2.03(1)	2.6(2)	3.88(8)	0.003(1)	0.03(3)	0.02(1)	0.02
0.41	6.2(8)	2.027(8)	3.16(4)	3.9(2)	0.003(1)	0.007(5)	0.03(3)	0.02
0.48	6.7(8)	2.033(8)	3.14(2)	3.9(1)	0.004(1)	0.004(3)	0.03(2)	0.02
0.52	6.9(8)	2.026(8)	3.13(4)	3.95(9)	0.005(1)	0.009(5)	0.02(1)	0.02
0.57	6.7(9)	2.025(8)	3.15(3)	3.8(1)	0.004(1)	0.006(4)	0.03(2)	0.02
0.64	5(1)	2.03(1)	1.60(5)	3.9(1)	0.001(2)	0.018(6)	0.03(2)	0.02
0.71	7(1)	2.025(9)	2.6(2)	3.19(4)	0.005(1)	0.02(2)	0.021(6)	0.02
0.75	6.4(8)	2.020(8)	3.13(2)	3.8(2)	0.003(1)	0.003(2)	0.03(2)	0.02
0.83	7.3(8)	2.011(8)	3.14(3)	3.8(1)	0.006(1)	0.005(3)	0.03(2)	0.02
0.87	6.6(7)	2.010(7)	3.11(2)	3.83(9)	0.004(1)	0.004(2)	0.02(1)	0.01
0.91	6.6(7)	2.004(7)	3.14(2)	3.84(7)	0.004(1)	0.001(2)	0.020(9)	0.01
0.95	6.9(8)	2.002(8)	3.15(3)	3.2(1)	0.005(1)	0.005(4)	0.03(2)	0.02

*The amplitude reduction factor was fixed at 0.78 for all fitting, calculated from fitting a crystalline iridium oxide. Fitting was performed in r space between 1- 4 Å, a fitting *k*-weight of 3, a hanning window with a tampering of 1 and a maximum *k* of 12 $Å^{-1}$.

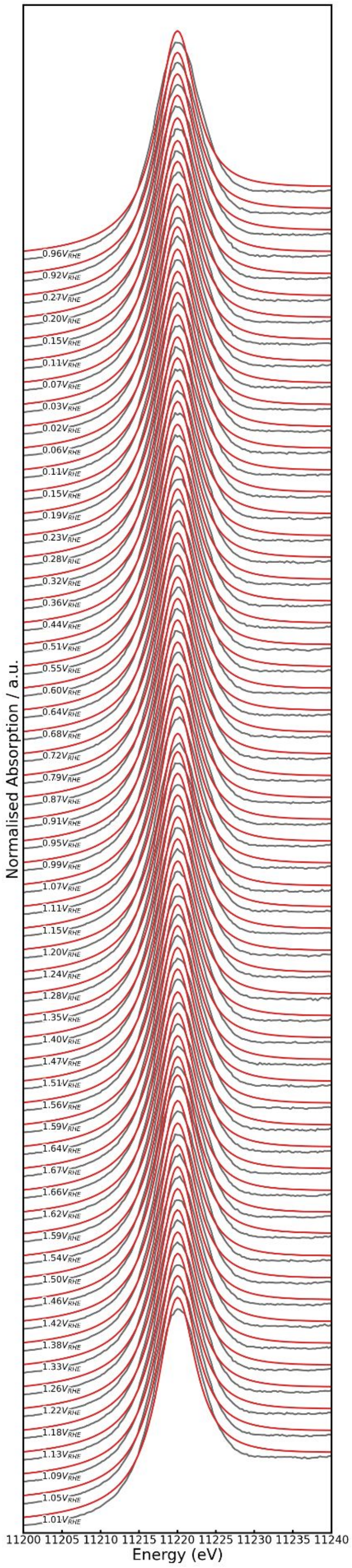

Figure S36 Normalized Ir L_{III} edge for the Premion™ (800 °C) electrodes as a function of the potential. The XANES were fit using an arctangent background function and the white line fit using a Lorentzian function. Above shows the overall fitted XANES for each potential measured. This fitting was used to extract the edge positions, areas, FWHMs.

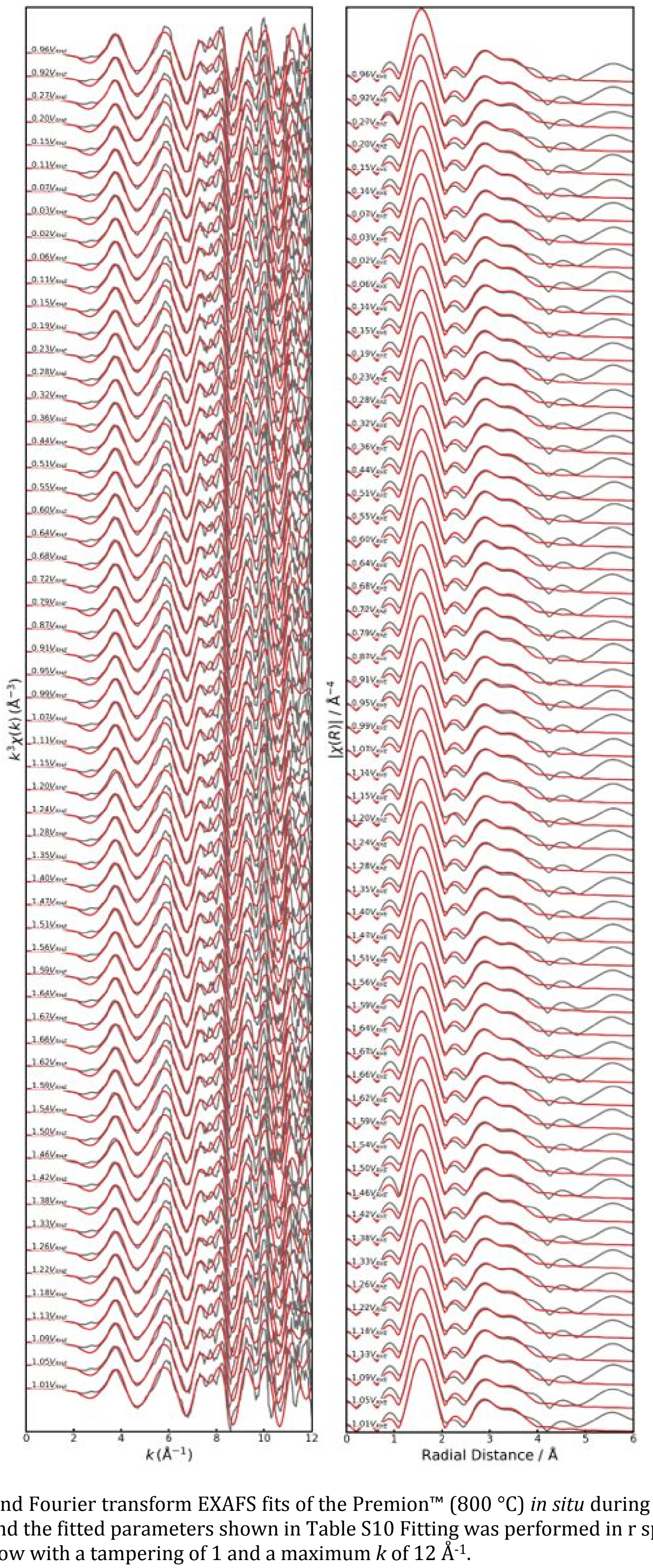

Figure S37 k^3-weighted *χ(k)* and Fourier transform EXAFS fits of the Premion™ (800 °C) *in situ* during a CV between 0-1.70 V_{RHE} fit using the model in Table S1 and the fitted parameters shown in Table S10 Fitting was performed in r space between 1- 4 Å, a fitting *k*-weight of 3, a hanning window with a tampering of 1 and a maximum *k* of 12 Å^{-1}.

Table S10 Fitted EXAFS parameters for the Premion™ (800 °C) *in situ* data.*

Potential / V_{RHE}	N_{O1}	Ir_{abs}-O_1 / Å	Ir_{abs}-Ir_1 / Å	Ir_{abs}-Ir_2 / Å	SS_{O1} / $Å^2$	SS_{Ir1} / $Å^2$	SS_{Ir2} / $Å^2$	R Factor
1.01	6.0(8)	1.971(8)	3.15(2)	3.54(2)	0.001(1)	0.004(2)	0.003(2)	0.02
1.05	6.0(8)	1.971(9)	3.16(2)	3.54(2)	0.001(1)	0.002(3)	0.003(2)	0.02
1.09	6.3(9)	1.970(9)	3.15(3)	3.54(2)	0.001(1)	0.001(3)	0.002(2)	0.02
1.13	6.2(8)	1.967(8)	3.14(2)	3.54(2)	0.001(1)	0.002(2)	0.002(2)	0.01
1.18	5.9(8)	1.968(8)	3.16(2)	3.53(2)	0.001(1)	0.003(2)	0.003(2)	0.02
1.22	6.2(8)	1.969(8)	3.15(2)	3.54(2)	0.001(1)	0.003(2)	0.003(2)	0.02
1.26	6.0(7)	1.969(8)	3.17(2)	3.53(2)	0.001(1)	0.001(3)	0.002(2)	0.01
1.33	6.2(8)	1.967(8)	3.15(2)	3.53(2)	0.001(1)	0.003(2)	0.003(2)	0.02
1.38	6.2(7)	1.971(8)	3.16(2)	3.54(2)	0.001(1)	0.002(2)	0.003(2)	0.01
1.42	6.0(8)	1.970(9)	3.16(2)	3.53(2)	0.001(1)	0.002(3)	0.003(2)	0.02
1.46	5.6(7)	1.967(7)	3.17(2)	3.53(2)	0.000(1)	0.003(2)	0.002(2)	0.01
1.50	6.3(9)	1.971(9)	3.15(2)	3.54(2)	0.001(1)	0.003(2)	0.003(2)	0.02
1.54	6.0(8)	1.968(8)	3.16(2)	3.53(2)	0.001(1)	0.003(2)	0.003(2)	0.02
1.59	6.2(8)	1.972(8)	3.16(2)	3.54(2)	0.001(1)	0.004(2)	0.004(2)	0.01
1.62	6.0(8)	1.970(9)	3.16(2)	3.53(2)	0.001(1)	0.003(2)	0.003(2)	0.02
1.66	6.0(8)	1.973(9)	3.16(2)	3.54(2)	0.001(1)	0.002(3)	0.002(2)	0.02
1.67	6.0(7)	1.971(7)	3.15(2)	3.55(2)	0.001(1)	0.003(2)	0.004(2)	0.01
1.64	6.2(7)	1.968(7)	3.15(2)	3.54(2)	0.001(1)	0.003(2)	0.002(2)	0.01
1.59	6.5(8)	1.970(8)	3.15(2)	3.54(2)	0.002(1)	0.002(2)	0.002(2)	0.01
1.56	6.3(8)	1.968(9)	3.15(2)	3.53(2)	0.002(1)	0.001(3)	0.003(2)	0.02
1.51	6.1(8)	1.969(8)	3.15(2)	3.53(2)	0.001(1)	0.001(3)	0.002(2)	0.01
1.47	6.2(8)	1.967(9)	3.15(2)	3.53(2)	0.001(1)	0.003(2)	0.003(2)	0.02
1.40	5.9(8)	1.971(8)	3.16(2)	3.54(2)	0.001(1)	0.003(2)	0.003(2)	0.01
1.35	6.2(8)	1.969(8)	3.16(2)	3.53(2)	0.001(1)	0.003(3)	0.003(2)	0.02

1.28	6.2(8)	1.969(8)	3.16(2)	3.54(2)	0.001(1)	0.002(2)	0.002(2)	0.01
1.24	6.2(8)	1.969(9)	3.15(2)	3.54(2)	0.001(1)	0.002(2)	0.003(2)	0.02
1.20	6(1)	1.97(1)	3.15(3)	3.54(2)	0.001(2)	0.002(3)	0.002(2)	0.02
1.15	6.2(8)	1.968(8)	3.15(2)	3.54(2)	0.001(1)	0.002(2)	0.002(2)	0.02
1.11	6.1(8)	1.968(8)	3.15(2)	3.54(2)	0.001(1)	0.002(2)	0.002(2)	0.02
1.07	6.2(8)	1.970(9)	3.15(2)	3.54(2)	0.001(1)	0.002(3)	0.002(2)	0.02
0.99	6.4(8)	1.967(8)	3.15(2)	3.53(2)	0.001(1)	0.001(3)	0.003(2)	0.02
0.95	6.1(8)	1.970(8)	3.16(2)	3.53(2)	0.001(1)	0.003(2)	0.002(2)	0.02
0.91	6.1(8)	1.971(8)	3.15(2)	3.54(2)	0.001(1)	0.003(2)	0.003(2)	0.01
0.87	6.3(9)	1.968(9)	3.16(2)	3.54(2)	0.001(1)	0.003(2)	0.003(2)	0.02
0.79	6.1(8)	1.967(8)	3.15(2)	3.54(2)	0.001(1)	0.004(2)	0.003(2)	0.01
0.72	6.0(8)	1.969(8)	3.16(2)	3.53(2)	0.001(1)	0.002(2)	0.003(2)	0.02
0.68	6.0(8)	1.974(9)	3.17(2)	3.54(2)	0.001(1)	0.003(2)	0.003(2)	0.02
0.64	6.1(8)	1.971(8)	3.16(2)	3.54(2)	0.001(1)	0.002(2)	0.003(2)	0.01
0.60	6.1(8)	1.970(8)	3.15(2)	3.54(2)	0.001(1)	0.003(2)	0.003(2)	0.02
0.55	6.1(8)	1.970(8)	3.15(2)	3.54(2)	0.001(1)	0.003(2)	0.002(2)	0.01
0.51	6.1(8)	1.972(9)	3.16(2)	3.54(2)	0.001(1)	0.002(3)	0.003(2)	0.02
0.44	6.1(7)	1.972(8)	3.15(2)	3.55(2)	0.001(1)	0.003(2)	0.003(2)	0.01
0.36	6.2(8)	1.970(8)	3.16(2)	3.53(2)	0.001(1)	0.002(3)	0.002(2)	0.02
0.32	6.3(8)	1.970(8)	3.15(2)	3.54(2)	0.001(1)	0.003(2)	0.002(2)	0.01
0.28	6.1(9)	1.973(9)	3.16(2)	3.54(2)	0.001(1)	0.003(3)	0.003(2)	0.02
0.23	6.1(8)	1.966(8)	3.14(2)	3.54(2)	0.001(1)	0.003(2)	0.003(2)	0.01
0.19	6.1(8)	1.972(9)	3.16(2)	3.54(2)	0.001(1)	0.003(2)	0.004(3)	0.02
0.15	6.1(8)	1.969(8)	3.16(2)	3.54(2)	0.001(1)	0.003(2)	0.003(2)	0.02
0.11	6.0(8)	1.971(8)	3.15(3)	3.54(2)	0.001(1)	0.001(3)	0.002(2)	0.02
0.06	6.0(7)	1.968(8)	3.16(2)	3.53(2)	0.001(1)	0.003(2)	0.003(2)	0.01

0.02	6.1(8)	1.972(8)	3.16(2)	3.55(2)	0.001(1)	0.003(2)	0.003(2)	0.01
0.03	6.2(8)	1.969(8)	3.15(2)	3.54(2)	0.001(1)	0.002(2)	0.002(2)	0.01
0.07	6.2(8)	1.970(8)	3.16(2)	3.54(2)	0.001(1)	0.003(2)	0.003(2)	0.01
0.11	6.0(7)	1.971(7)	3.15(2)	3.54(2)	0.001(1)	0.004(2)	0.004(2)	0.01
0.15	6.0(8)	1.965(8)	3.15(2)	3.53(2)	0.001(1)	0.003(2)	0.003(2)	0.02
0.20	6.0(8)	1.971(9)	3.15(2)	3.54(2)	0.001(1)	0.002(2)	0.003(2)	0.02
0.27	6.2(6)	1.962(6)	3.14(2)	3.53(1)	0.0009(9)	-0.001(3)	0.001(1)	0.01
0.92	6.1(7)	1.969(7)	3.15(2)	3.54(2)	0.001(1)	0.002(2)	0.003(2)	0.01
0.96	5.9(7)	1.970(8)	3.15(2)	3.54(2)	0.000(1)	0.003(2)	0.003(2)	0.01

*The amplitude reduction factor was fixed at 0.78 for all fitting, calculated from fitting a crystalline iridium oxide. Fitting was performed in r space between 1- 4 Å, a fitting *k*-weight of 3, a hanning window with a tampering of 1 and a maximum *k* of 12 $Å^{-1}$.

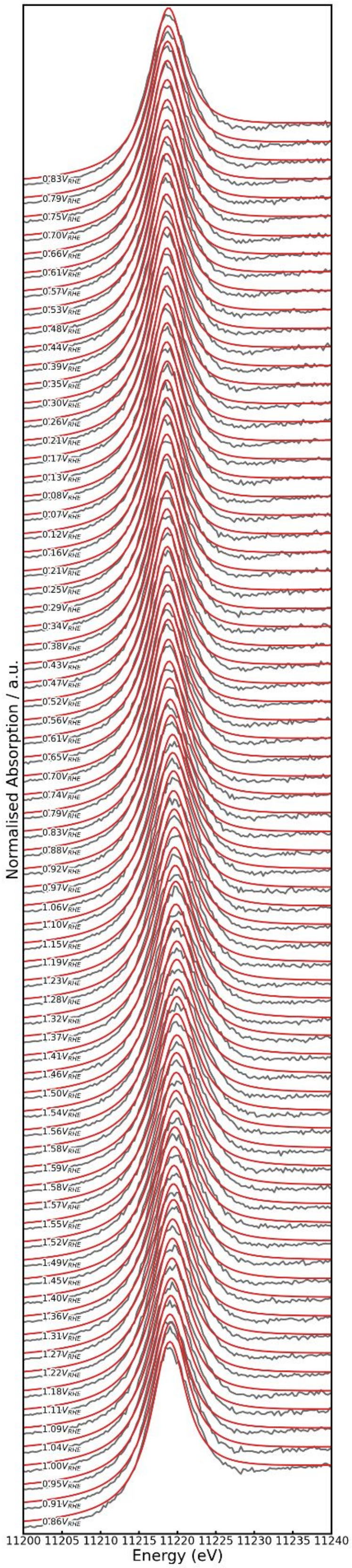

Figure S38 Normalized Ir L_{III} edge for the Hydrated IrO_x electrodes as a function of the potential. The XANES were fit using an arctangent background function and the white line fit using a Lorentzian function. Above shows the overall fitted XANES for each potential measured. This fitting was used to extract the edge positions, areas, FWHMs.

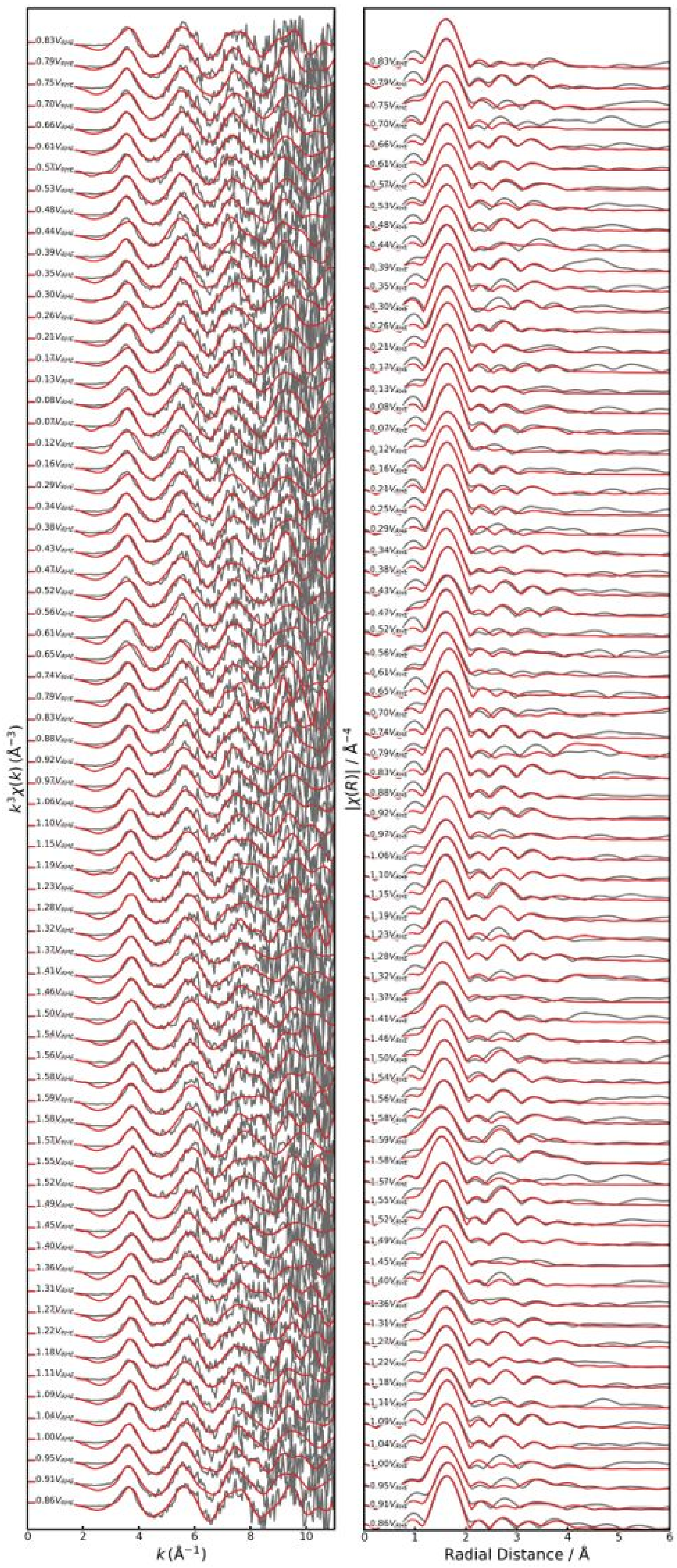

Figure S39 k^3-weighted *χ(k)* and Fourier transform EXAFS fits of the Hydrated IrO_x *in situ* during a CV between 0-1.70 V_{RHE} fit using the model in Table S1 and the fitted parameters shown inTable S11 Fitting was performed in r space between 1- 4 Å, a fitting *k*-weight of 3, a hanning window with a tampering of 1 and a maximum *k* of 12 Å^{-1}.

Table S11 Fitted EXAFS parameters for the Hydrated IrO_x *in situ* data.*

Potential / V_{RHE}	N_{O1}	Ir_{abs}-O_1 / Å	Ir_{abs}-Ir_1 / Å	Ir_{abs}-Ir_2 / Å	SS_{O1} / Å^2	SS_{Ir1} / Å^2	SS_{Ir2} / Å^2	R Factor
0.86	5.7(6)	2.02(1)	3.13(4)	3.70(3)	0.001(2)	0.005(5)	0.006(3)	0.03
0.91	6(2)	2.04(2)	2.6(1)	4.00(9)	0.004(3)	0.02(2)	0.02(1)	0.06
0.95	7(1)	2.01(2)		3.89(3)	0.007(2)		0.009(4)	0.05
1	6(2)	2.00(3)	1.7(2)	3.9(1)	0.004(4)	0.03(4)	0.02(1)	0.05
1.04	6(1)	2.02(1)	3.15(3)	3.7(1)	0.004(2)	0.003(4)	0.02(2)	0.04
1.09	5(1)	2.02(1)	3.11(3)	3.76(5)	0.002(2)	0.002(3)	0.011(6)	0.05
1.11	8(2)	2.01(2)	3.5(1)	3.7(1)	0.008(3)	0.00(1)	0.02(2)	0.07
1.18	6(1)	2.00(1)	3.15(2)	3.78(6)	0.005(2)	-0.002(2)	0.015(8)	0.03
1.22	6(2)	2.00(2)	3.14(5)	3.70(4)	0.006(3)	0.005(7)	0.007(4)	0.07
1.27	6(1)	1.99(1)	3.13(2)	3.74(4)	0.007(2)	0.000(2)	0.013(5)	0.03
1.31	8(2)	1.99(2)	3.48(6)	3.73(8)	0.010(3)	0.000(6)	0.01(1)	0.06
1.36	8(2)	1.96(2)	3.49(6)	3.7(1)	0.008(3)	0.002(7)	0.02(2)	0.05
1.4	6(1)	1.97(2)	2.66(8)	3.7(1)	0.004(2)	0.01(1)	0.02(2)	0.06
1.45	7(1)	1.98(2)	2.57(7)	3.8(2)	0.007(2)	0.02(1)	0.03(2)	0.04
1.49	8(2)	1.98(2)	3.13(4)	3.71(4)	0.009(3)	0.002(4)	0.010(5)	0.07
1.52	6(1)	1.95(1)	3.06(3)	3.65(7)	0.004(2)	0.001(3)	0.02(1)	0.04
1.55	7(1)	1.95(1)	3.08(2)	3.66(7)	0.007(2)	-0.001(2)	0.018(9)	0.03
1.57	6(1)	1.94(1)	2.6(1)	3.7(5)	0.003(2)	0.02(2)	0.04(7)	0.05
1.58	6(2)	1.95(2)	3.46(4)	3.7(1)	0.008(4)	-0.003(3)	0.02(2)	0.09
1.59	8(2)	1.93(2)	3.06(2)	3.67(6)	0.011(3)	0.000(2)	0.014(8)	0.06
1.58	5(1)	1.96(2)	3.48(6)	3.8(1)	0.004(2)	0.002(6)	0.02(2)	0.06
1.56	6(1)	1.96(1)	3.11(3)	3.7(2)	0.005(2)	0.002(3)	0.02(2)	0.05
1.54	5(1)	1.97(2)	3.20(3)	3.8(2)	0.003(2)	0.000(3)	0.03(4)	0.06
1.5	6(1)	1.95(1)	3.08(3)	3.65(8)	0.006(2)	0.003(3)	0.02(1)	0.04

1.46	6(2)	1.96(2)	4.4(2)	3.80(5)	0.008(3)	0.00(2)	0.011(5)	0.06
1.41	8(1)	1.96(1)	3.08(3)	3.66(5)	0.009(2)	0.005(4)	0.014(6)	0.03
1.37	7(1)	1.97(1)	3.1(1)	3.7(1)	0.009(2)	0.02(2)	0.02(1)	0.03
1.32	6(1)	1.99(2)	4.63(9)	3.8(1)	0.007(3)	-0.005(9)	0.02(2)	0.06
1.28	7(1)	1.98(1)	3.10(2)	3.69(3)	0.007(2)	-0.001(2)	0.010(3)	0.03
1.23	6(2)	1.98(2)	4.35(8)	3.75(6)	0.006(3)	-0.003(8)	0.012(7)	0.06
1.19	6.0(7)	1.978(8)	3.30(3)	3.12(2)	0.006(1)	0.000(3)	0.008(2)	0.02
1.15	6(1)	1.99(1)	3.11(3)	3.65(4)	0.005(2)	0.000(3)	0.009(5)	0.05
1.1	6(1)	2.00(1)	3.15(4)	3.77(5)	0.005(2)	0.003(4)	0.012(6)	0.05
1.06	6(1)	2.00(1)	3.12(2)	3.8(1)	0.005(2)	0.002(3)	0.02(2)	0.04
0.97	6(1)	2.01(1)	4.3(3)	3.9(1)	0.005(2)	0.01(4)	0.02(2)	0.04
0.92	7(1)	2.03(1)	2.5(1)	3.12(3)	0.005(2)	0.02(1)	0.014(4)	0.04
0.88	5.4(8)	2.018(9)	3.16(4)	3.73(6)	0.002(2)	0.005(5)	0.014(7)	0.03
0.83	5.5(8)	2.027(8)	3.13(3)	3.78(3)	0.002(1)	0.003(3)	0.009(3)	0.02
0.79	6(1)	2.03(2)		4.56(5)	0.002(2)		-0.001(4)	0.07
0.74	5.6(9)	2.03(1)	3.14(3)	3.72(5)	0.002(2)	0.003(4)	0.012(6)	0.03
0.7	6(1)	2.02(2)	9.53(8)	3.58(6)	0.004(2)	-0.038(6)	0.012(6)	0.06
0.65	6(2)	2.06(2)	3.4(1)	3.63(9)	0.002(3)	0.00(1)	0.01(1)	0.11
0.61	8(2)	2.02(2)	3.36(7)	3.58(6)	0.007(3)	0.000(7)	0.008(7)	0.06
0.56	6(1)	2.03(1)	3.7(3)	3.9(2)	0.004(2)	0.01(3)	0.02(3)	0.05
0.52	6(1)	2.03(1)	3.39(6)	3.59(8)	0.003(2)	0.000(8)	0.01(1)	0.05
0.47	8(2)	2.04(2)	3.13(4)	3.72(4)	0.008(3)	0.003(4)	0.009(4)	0.05
0.43	6(1)	2.04(1)	3.17(2)	3.76(4)	0.002(2)	0.000(2)	0.010(5)	0.03
0.38	6.1(7)	2.029(8)	3.02(4)	3.22(5)	0.003(1)	0.006(5)	0.019(7)	0.02
0.34	6(1)	2.05(1)	3.44(4)	4.04(6)	0.004(2)	0.001(4)	0.011(8)	0.05
0.29	6(2)	2.05(2)		3.6(1)	0.003(3)		0.01(1)	0.1

0.25	6(1)	2.04(1)		3.59(7)	0.002(2)		0.014(8)	0.05
0.21	5.0(8)	2.034(9)	18.0(2)	3.9(1)	0.000(1)	-0.06(1)	0.02(1)	0.03
0.16	5.7(9)	2.03(1)	2.4(1)	3.62(5)	0.002(1)	0.02(2)	0.015(6)	0.02
0.12	8(2)	2.05(2)	3.40(7)	3.60(6)	0.007(3)	-0.002(8)	0.005(8)	0.1
0.07	5(1)	2.05(1)	3.0(2)	3.2(1)	0.001(2)	0.01(2)	0.02(1)	0.05
0.08	5.0(8)	2.034(9)	3.09(7)	3.63(3)	0.001(1)	0.01(1)	0.008(3)	0.03
0.13	7(1)	2.04(1)	3.2(1)	3.63(8)	0.005(2)	0.01(2)	0.02(1)	0.03
0.17	5.1(8)	2.03(1)	3.39(8)	3.67(5)	0.001(2)	0.004(8)	0.009(7)	0.03
0.21	6(1)	2.04(1)	3.2(2)	3.65(8)	0.003(2)	0.02(3)	0.01(1)	0.04
0.26	5.1(9)	2.04(1)	3.16(4)	3.70(5)	0.002(2)	0.006(5)	0.012(7)	0.03
0.3	6(1)	2.00(3)	1.90(5)	3.70(6)	-0.001(4)	0.003(6)	0.009(5)	0.07
0.35	6(1)	2.04(2)	2.74(9)	3.90(5)	0.002(2)	0.01(1)	0.011(6)	0.07
0.39	7(1)	2.03(1)	3.18(3)	3.80(8)	0.004(2)	0.002(3)	0.02(1)	0.03
0.44	6(1)	2.04(2)	2.68(6)	3.58(3)	0.003(2)	0.011(7)	0.008(3)	0.05
0.48	7(1)	2.04(1)	3.15(2)	3.9(1)	0.005(2)	0.002(2)	0.02(2)	0.02
0.53	6(1)	2.03(1)	2.80(4)	3.93(8)	0.003(2)	0.008(5)	0.02(1)	0.04
0.57	5.1(8)	2.048(9)	3.3(2)	3.2(1)	0.001(1)	0.01(2)	0.02(2)	0.03
0.61	5.7(7)	2.038(8)	3.15(4)	3.8(2)	0.003(1)	0.008(5)	0.03(2)	0.02
0.66	5.9(7)	2.024(7)	3.13(2)	3.8(2)	0.003(1)	0.001(2)	0.03(2)	0.02
0.7	6(1)	2.01(2)		3.58(9)	0.003(2)		0.02(1)	0.06
0.75	8(2)	2.04(2)	2.56(3)	3.94(7)	0.008(2)	0.008(4)	0.016(9)	0.05
0.79	5.4(8)	2.022(9)	3.12(2)	3.80(8)	0.002(2)	0.001(2)	0.02(1)	0.03
0.83	6(1)	2.02(1)	3.4(1)	3.64(5)	0.003(2)	0.00(1)	0.005(5)	0.04

*The amplitude reduction factor was fixed at 0.78 for all fitting, calculated from fitting a crystalline iridium oxide. Fitting was performed in r space between 1- 4 Å, a fitting *k*-weight of 3, a hanning window with a tampering of 1 and a maximum *k* of 12 $Å^{-1}$.

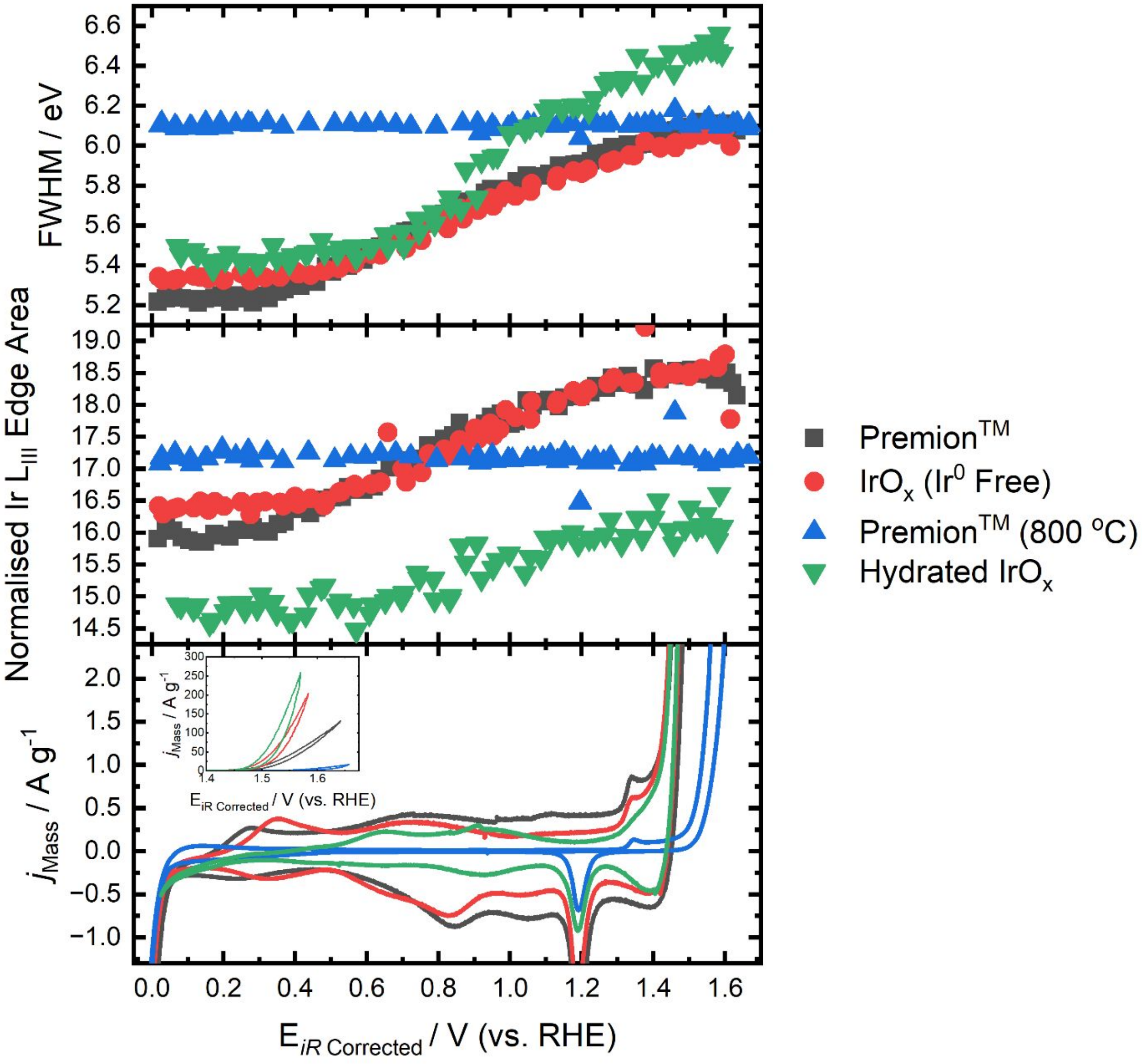

Figure S40 White line area and FWHM of the Ir L_{III} edge as a function of the applied potential. White line area is calculated by integrating the Ir L_{III} edge with a fitted arctangent baseline and the full width at half maximum (FWHM) of a Lorentzian function fitted to the Ir L_{III} edge using an arctangent baseline function. Data was collected during a CV (0.001 V s^{-1}) in the SPEC-XAS cell with a 1 M H_2SO_4 under constant N_2 purging. The iridium metal loadings determined by XRF of the electrodes prior to testing are 0.19 mg_{Ir} cm^{-2} for the Premion™, 0.17 mg_{Ir} cm^{-2} for the IrO_x (Ir^0 Free), 0.41 mg_{Ir} cm^{-2} for the Premion™ (800 °C) and 0.17 mg_{Ir} cm^{-2} for the Hydrated IrO_x.

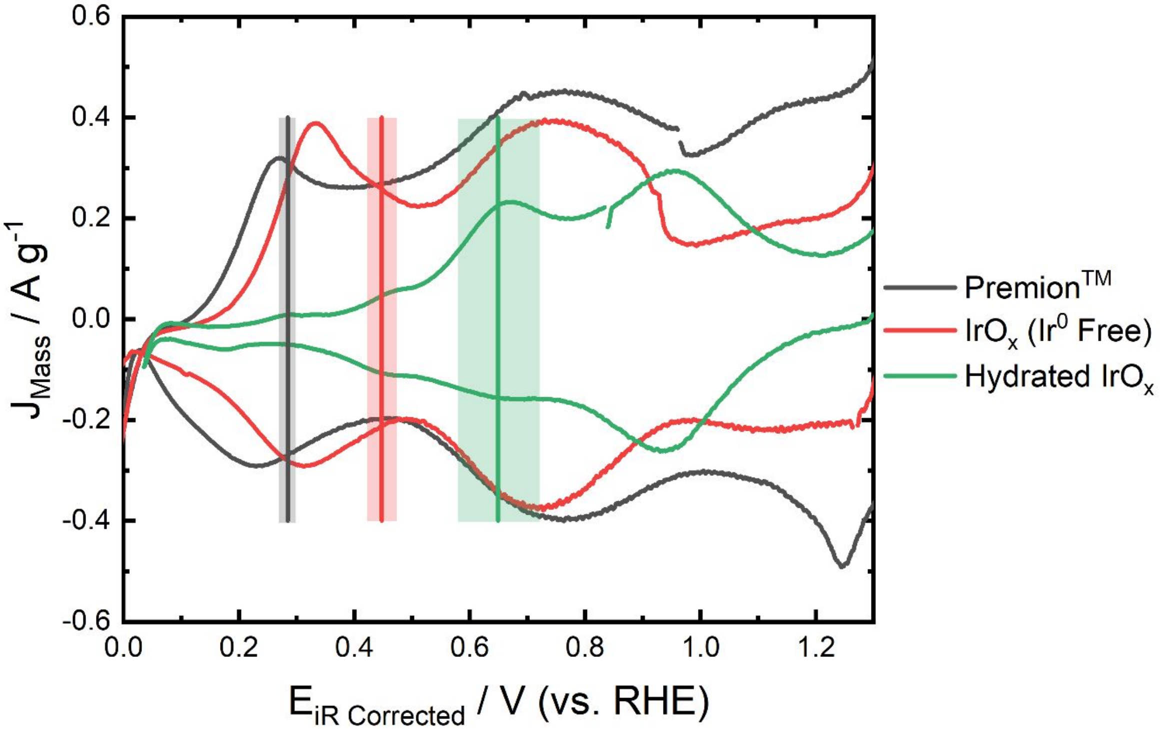

Figure S41 Start of the plateau in oxidation state for each of the iridium overlaid onto the HER corrected 1 mV s^{-1} *in situ* SPEC-XAS cell CVs. The vertical lines represent the start of the plateau region of the oxidation state with the shaded region indicating the associated error. The CVs where corrected by subtracting a fitted Tafel from the raw data to generate an approximate HER corrected CV. This allows better visualisation of there where the plateau region is occurring in the CVs.

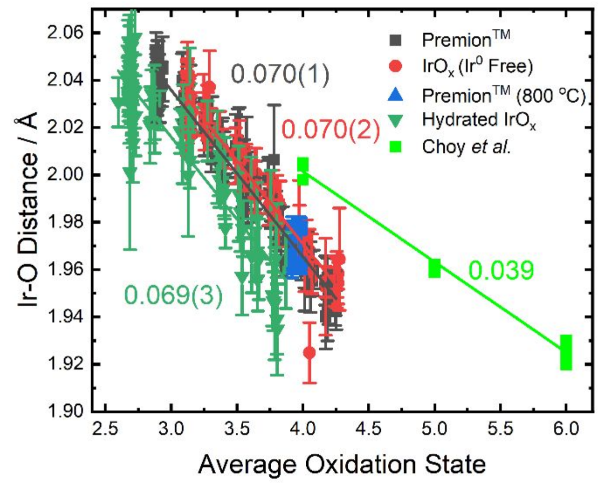

Figure S42 Comparison of the measured Ir-O bond length contraction as a function of the oxidation state to literature values for Perovskite iridium oxides from Choy *et al.*[11]

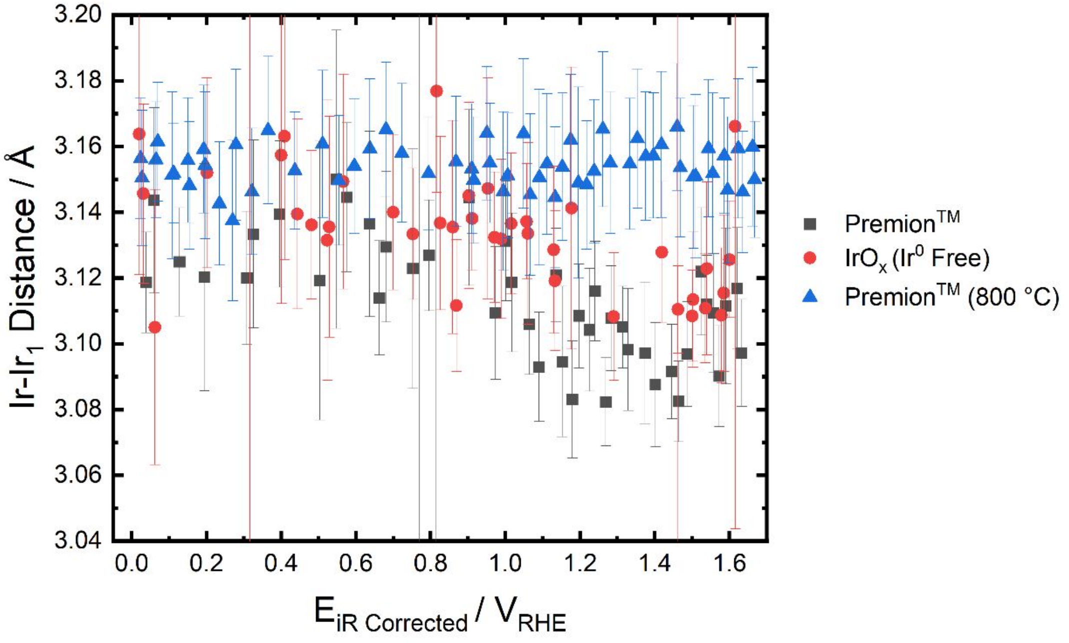

Figure S43 Comparison of the Ir-Ir$_1$ Distance as a function of the *iR* corrected potential for the Premion™, IrO_x (Ir^0 Free) and Premion™ (800 °C) materials. The Hydrated IrO_x was not included in the comparison as there is very little structure in the second and third shells.

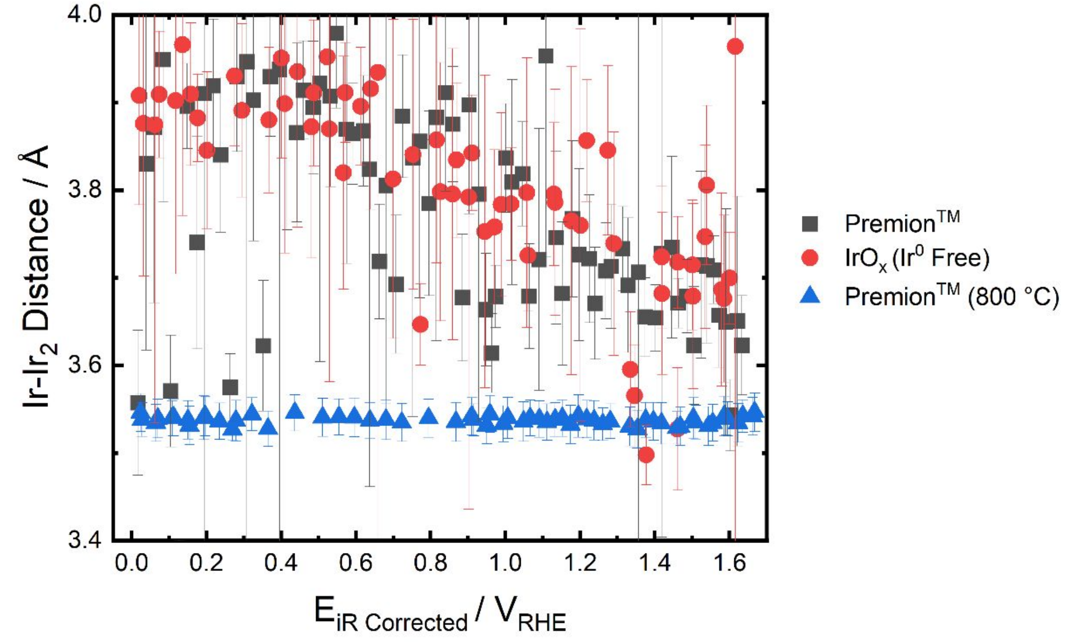

Figure S44 Comparison of the Ir-Ir$_2$ Distance as a function of the *iR* corrected potential for the Premion™, IrO_x (Ir^0 Free) and Premion™ (800 °C) materials. The Hydrated IrO_x was not included in the comparison as the structure shows very little structure in the second and third shells.

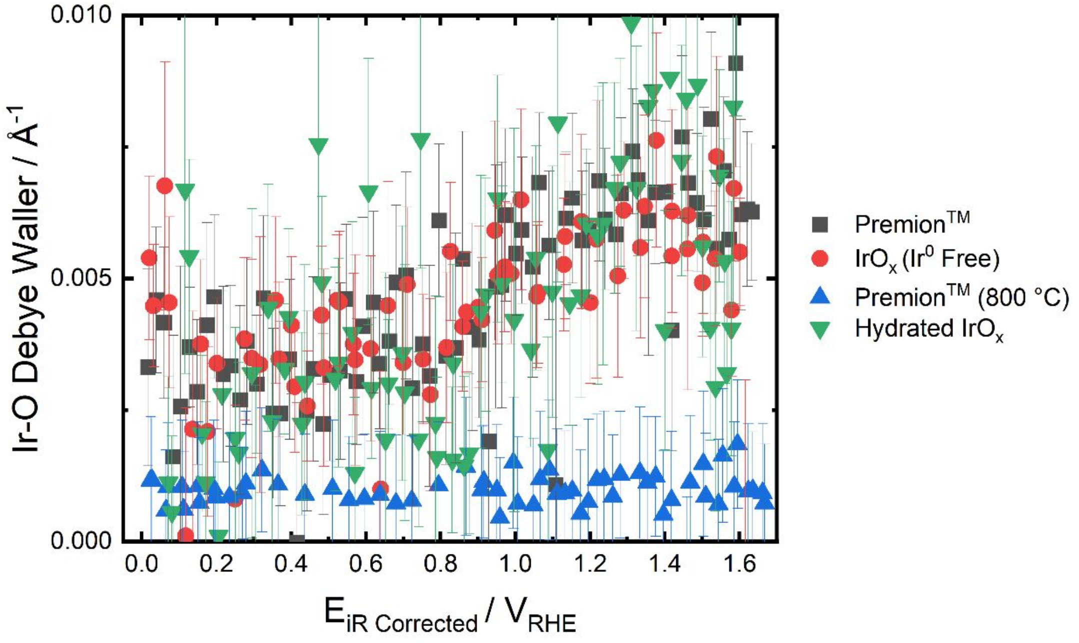

Figure S45 Comparison of the Ir-O Debye Waller factors as a function of the *iR* corrected potential.

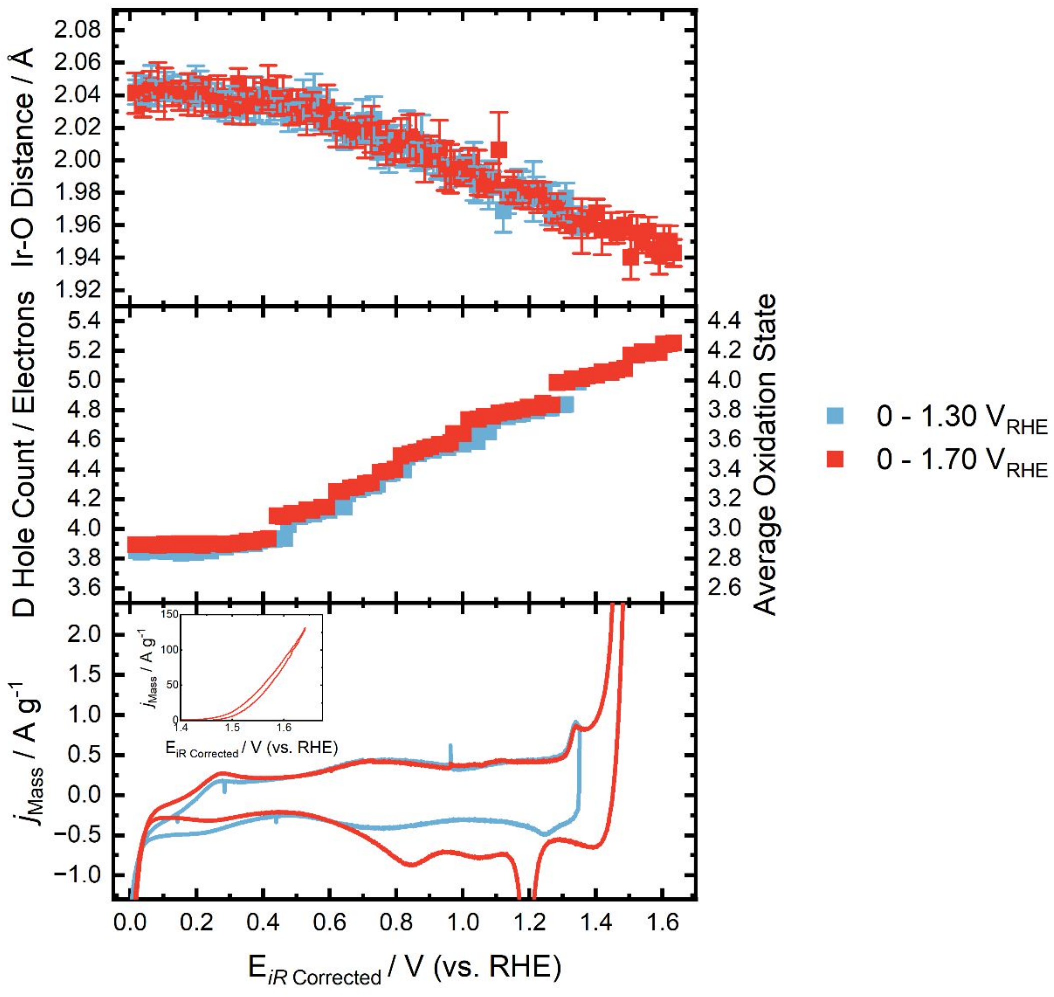

Figure S46 Comparison of CVs and oxidation states with upper potential limits of 1.64 V and 1.35 V_{RHE} (*iR* corrected) for the Premion™ catalyst. Measurements were made in the SPEC-XAS cell in flowing (5 ml min^{-1}) 1 M H_2SO_4 electrolyte. Under constant N_2 purging.

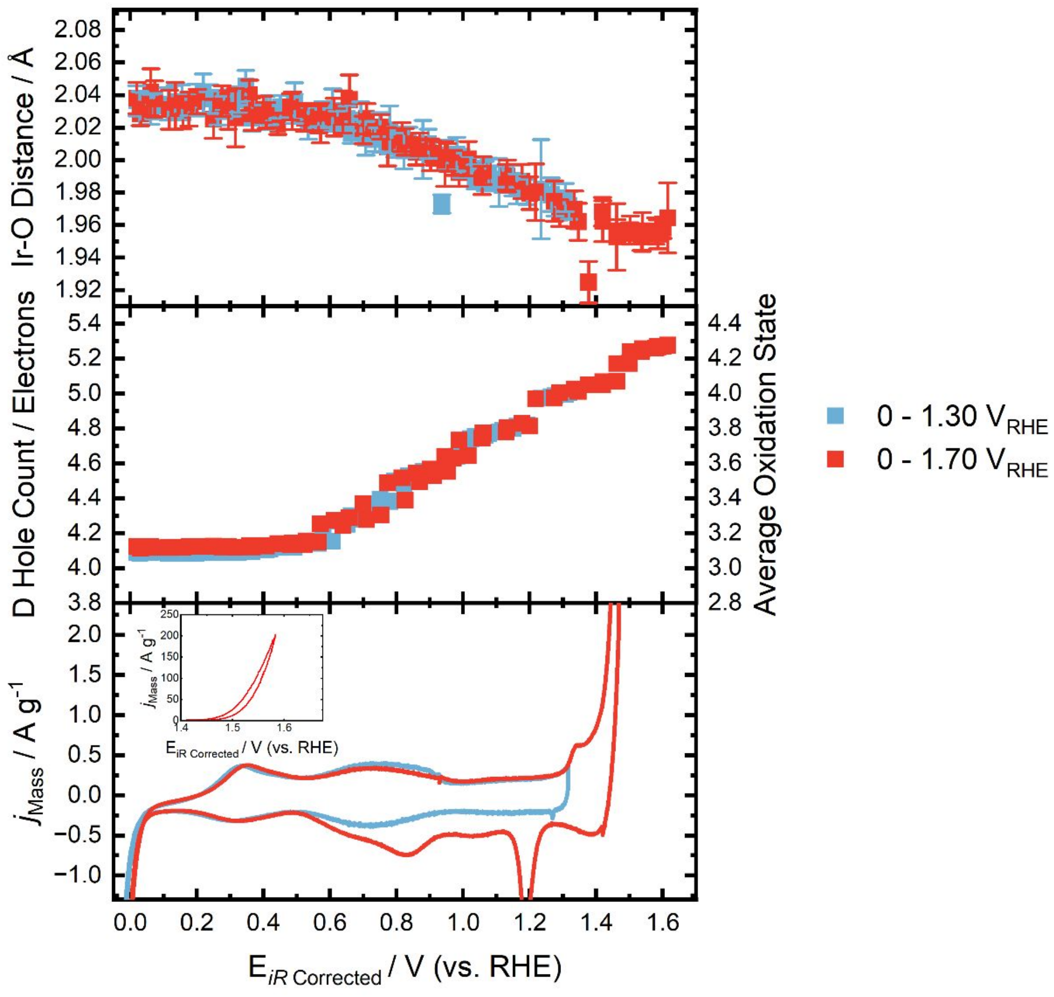

Figure S47 Comparison of CVs and oxidation states with upper potential limits of 1.64 V and 1.35 V_{RHE} (*iR* corrected) for the IrO_x (Ir^0 Free) catalyst. Measurements were made in the SPEC-XAS cell in flowing (5 ml min^{-1}) 1 M H_2SO_4 electrolyte. Under constant N_2 purging.

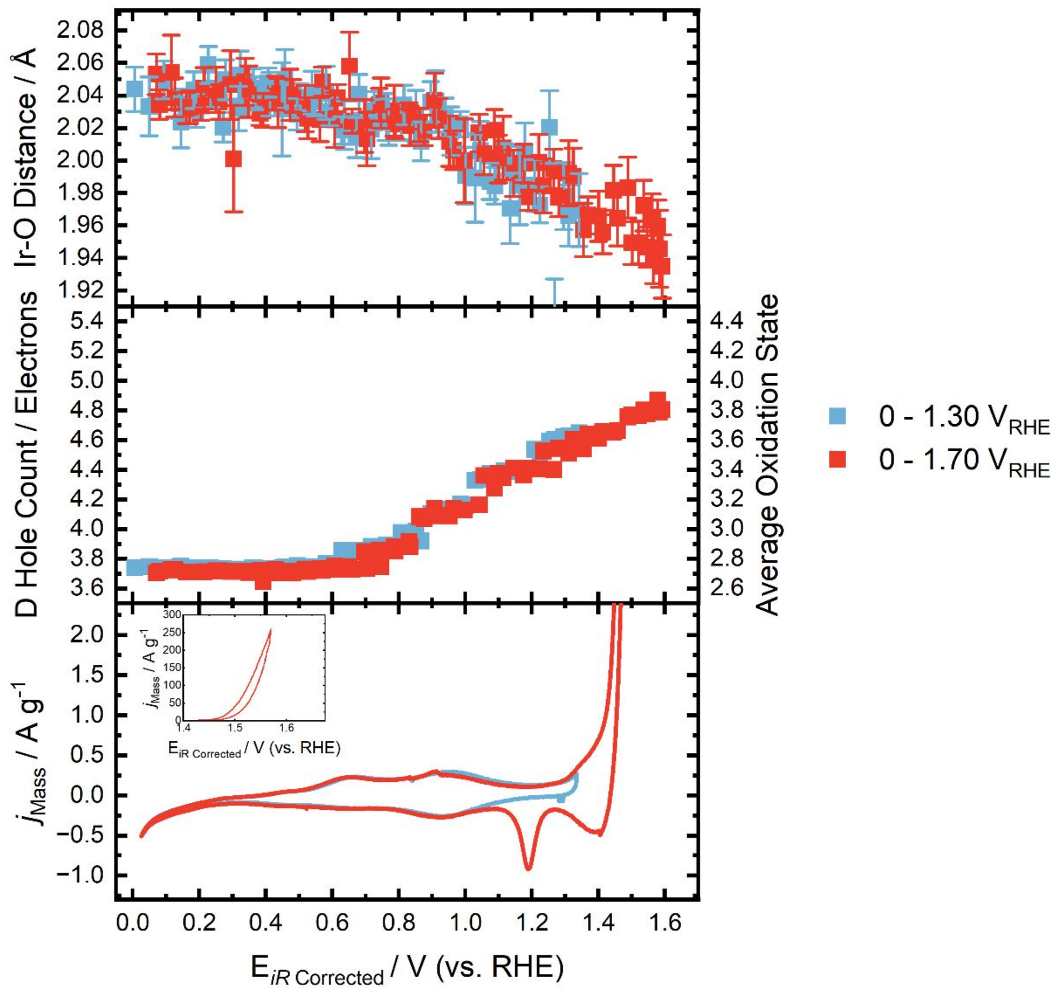

Figure S48 Comparison of CVs and oxidation states with upper potential limits of 1.64 V and 1.35 V_{RHE} (*iR* corrected) for the Hydrated IrO_x catalyst. Measurements were made in the SPEC-XAS cell in flowing (5 ml min^{-1}) 1 M H_2SO_4 electrolyte. Under constant N_2 purging.

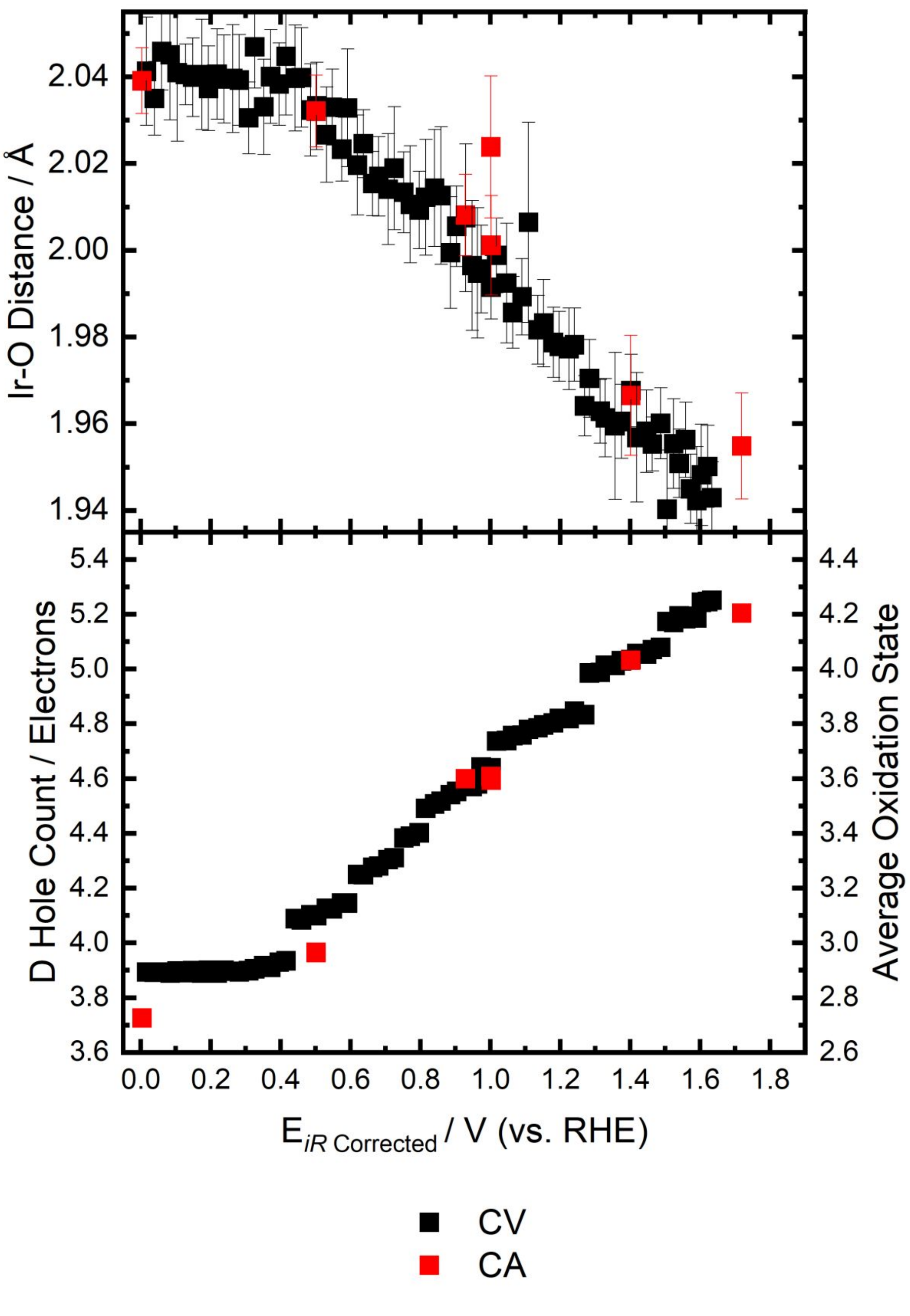

Figure S49 Comparison of oxidation state and iridium oxygen distance measured during a cyclic voltammogram (1 mV s^{-1}) and from the merged spectra from a 30-minute chronoamperometric hold of the Premion™ catalyst. Both experiments were conducted in the SPEC-XAS cell with a 1M H_2SO_4 electrolyte flowing at 5 ml min^{-1} and with constant N_2 purging.

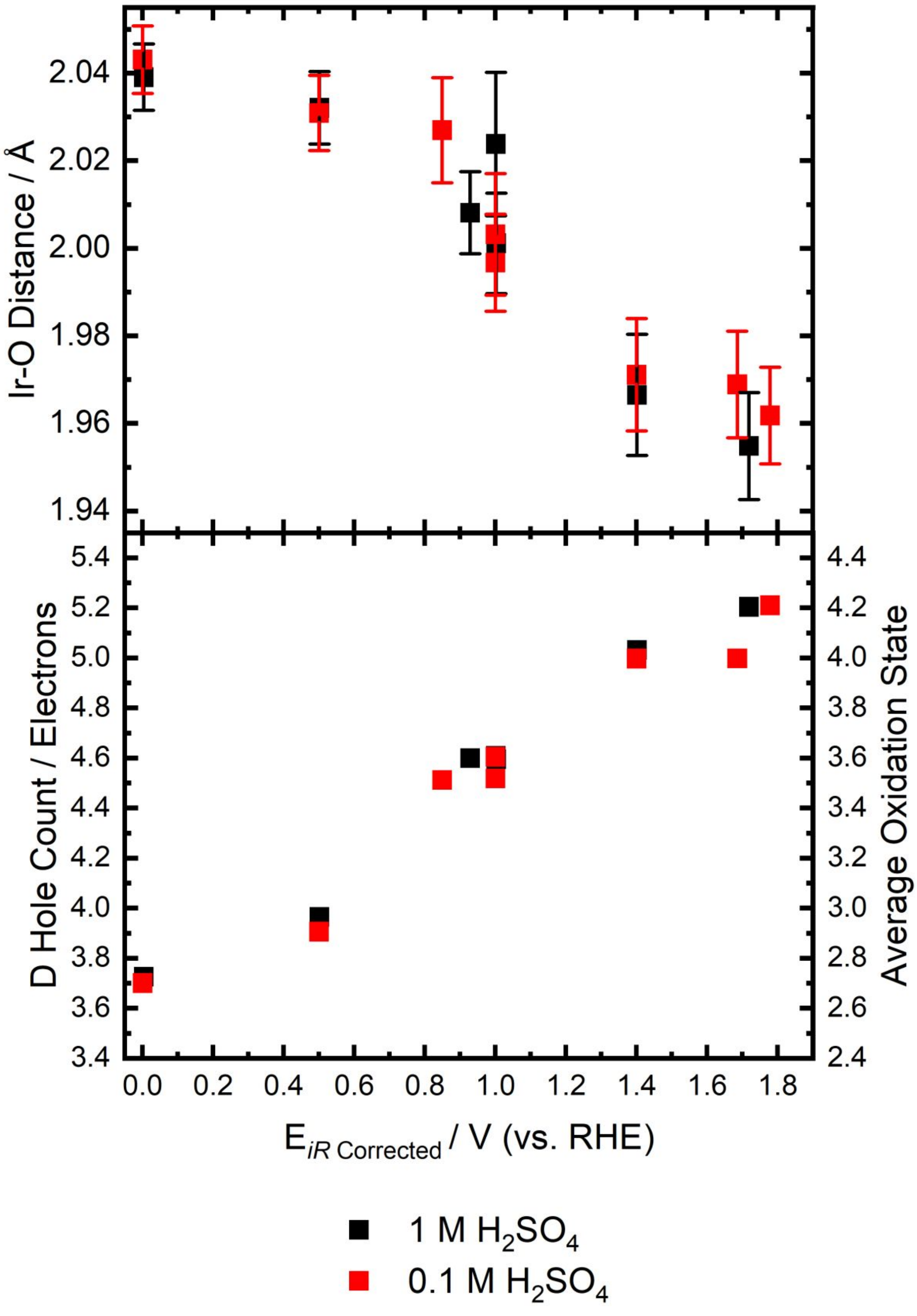

Figure S50 Comparison between the oxidation state and the iridium-oxygen distance of the Premion™ iridium oxide in 1 M and 0.1 M H_2SO_4 solutions. Both experiments were conducted in the SPEC-XAS cell with the respective electrolyte flowing at 5 ml min^{-1} and with constant N_2 purging.

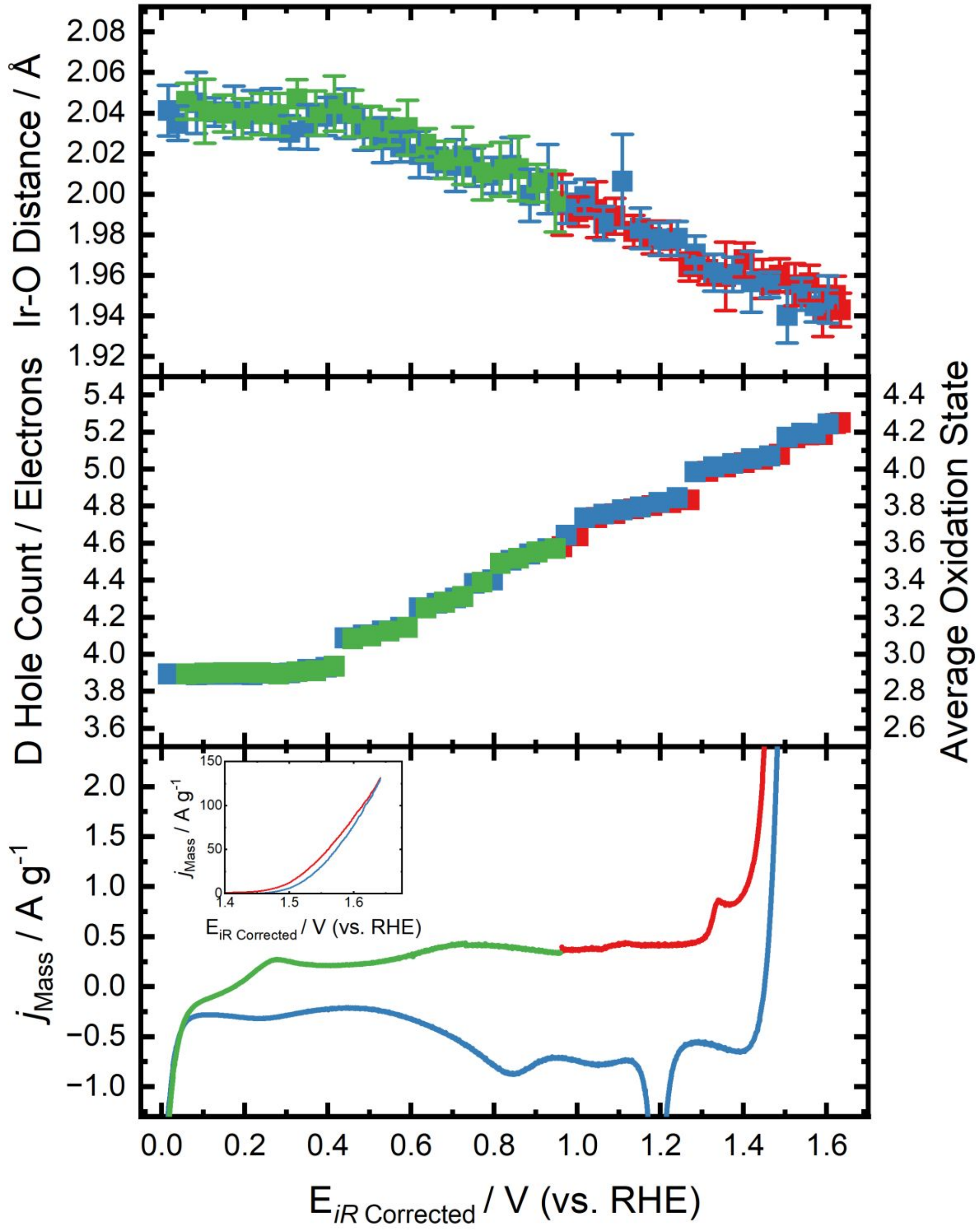

Figure S51 Potential resolved oxidation states and Ir-O distances with corresponding mass-normalised cyclic voltammetry for the Premion™. The oxidation states and CV have been split in to the corresponding cycles to see deviation between forward and reverse scans.

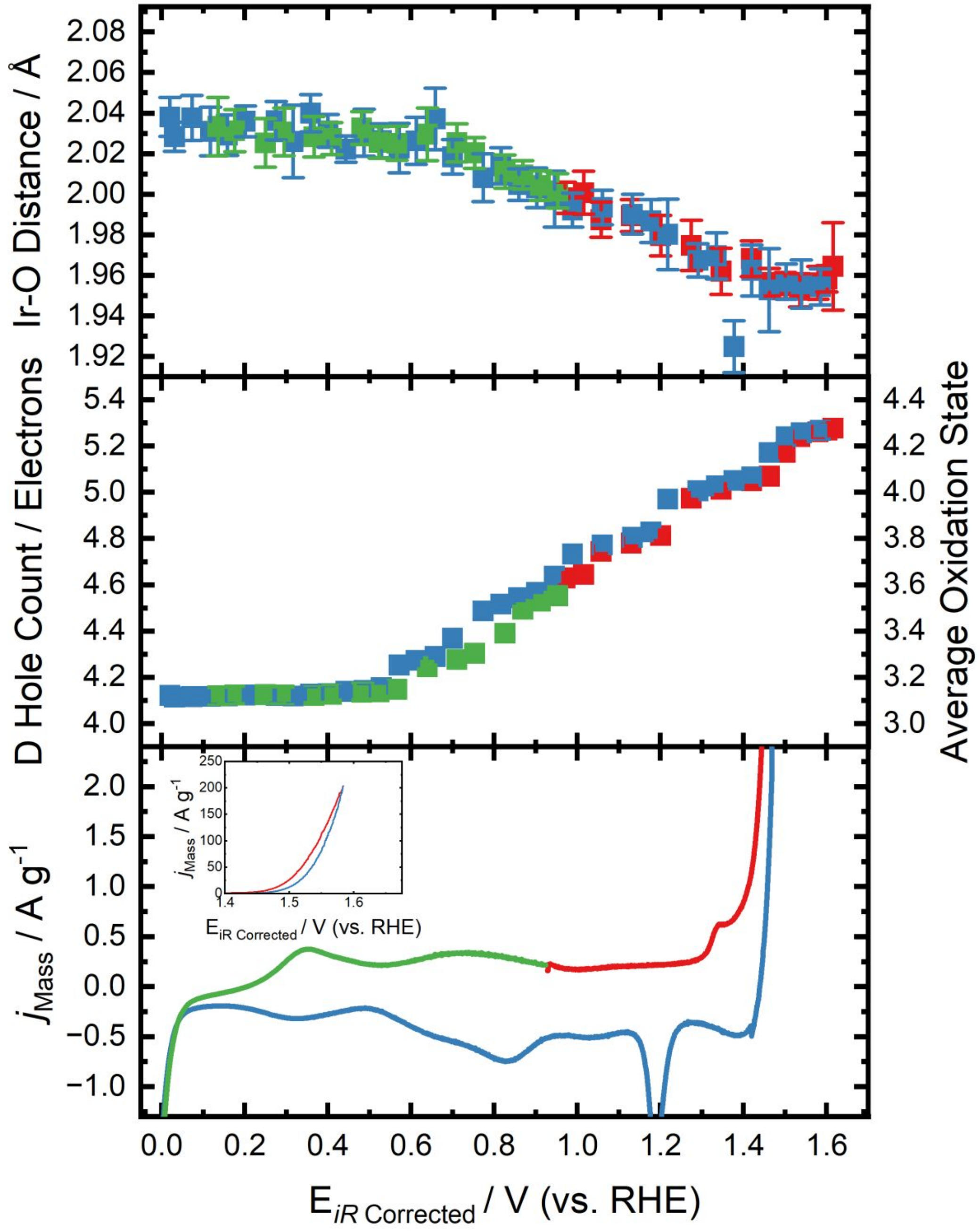

Figure S52 Potential resolved oxidation states and Ir-O distances with corresponding mass-normalised cyclic voltammetry for the IrO_x (Ir^0 Free). The oxidation states and CV have been split in to the corresponding cycles to see deviation between forward and reverse scans.

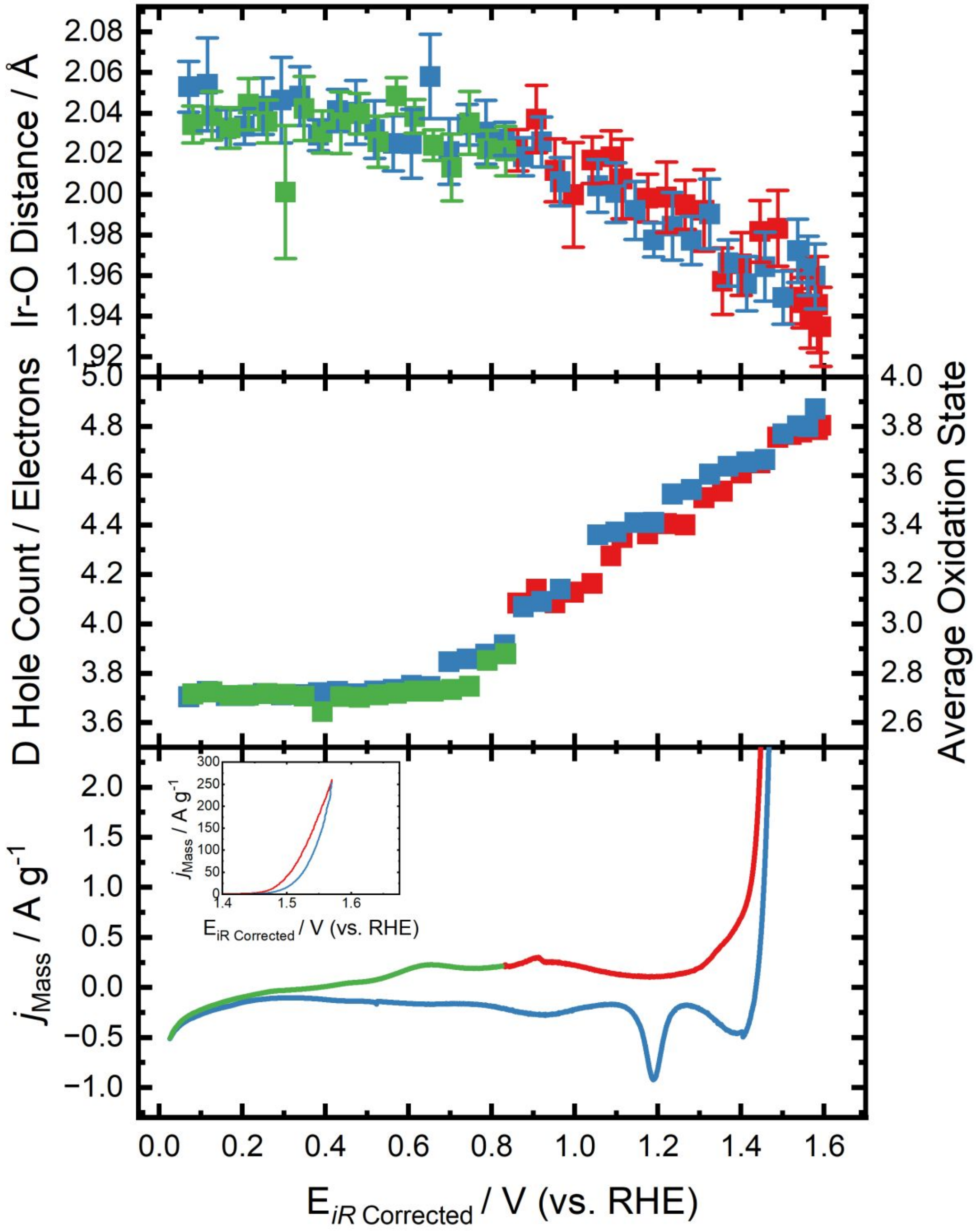

Figure S53 Potential resolved oxidation states and Ir-O distances with corresponding mass-normalised cyclic voltammetry for the Hydrated IrO_x. The oxidation states and CV have been split in to the corresponding cycles to see deviation between forward and reverse scans.

REFERENCES

(1) MDR XAFS DB. National Institute for Materials Science.
(2) Ishii, M.; Tanabe, K.; Matsuda, A.; Ofuchi, H.; Matsumoto, T.; Yaji, T.; Inada, Y.; Nitani, H.; Kimura, M.; Asakura, K. Integration of X-ray absorption fine structure databases for data-driven materials science. *Science and Technology of Advanced Materials: Methods* **2023**, *3* (1), 2197518.
(3) XAFS spectrum of Iridium. Science, N. I. f. M., Ed.; https://mdr.nims.go.jp/concern/datasets/8s45qc21m.
(4) XAFS spectrum of Iridium(III) acetylacetonate. Science, N. I. f. M., Ed.; https://mdr.nims.go.jp/concern/datasets/5712m942h.
(5) XAFS spectrum of Iridium(III) chloride, anhydrous. Science, N. I. f. M., Ed.; https://mdr.nims.go.jp/concern/datasets/m900nx089.
(6) XAFS spectrum of Iridium(III) chloride, hydrous. Science, N. I. f. M., Ed.; https://mdr.nims.go.jp/concern/datasets/m326m4675.
(7) XAFS spectrum of Iridium(IV) chloride, anhydrous. Science, N. I. f. M., Ed.; https://mdr.nims.go.jp/concern/datasets/1831cn75x.
(8) XAFS spectrum of Iridium(IV) oxide. Science, N. I. f. M., Ed.; https://mdr.nims.go.jp/concern/datasets/zc77ss51m.
(9) Tensi, L.; Yakimov, A. V.; Trotta, C.; Domestici, C.; De Jesus Silva, J.; Docherty, S. R.; Zuccaccia, C.; Copéret, C.; Macchioni, A. Single-Site Iridium Picolinamide Catalyst Immobilized onto Silica for the Hydrogenation of CO2 and the Dehydrogenation of Formic Acid. *Inorganic Chemistry* **2022**, *61* (27), 10575-10586. DOI: 10.1021/acs.inorgchem.2c01640.
(10) Bolzan, A. A.; Fong, C.; Kennedy, B. J.; Howard, C. J. Structural Studies of Rutile-Type Metal Dioxides. *Acta Crystallographica Section B* **1997**, *53* (3), 373-380. DOI: https://doi.org/10.1107/S0108768197001468 (accessed 2025/03/11).
(11) Choy, J.-H.; Kim, D.-K.; Hwang, S.-H.; Demazeau, G.; Jung, D.-Y. XANES and EXAFS Studies on the Ir-O Bond Covalency in Ionic Iridium Perovskites. *Journal of the American Chemical Society* **1995**, *117* (33), 8557-8566. DOI: 10.1021/ja00138a010.
